# Health effects associated with consumption of unprocessed red meat: a Burden of Proof study

**DOI:** 10.1038/s41591-022-01968-z

**Published:** 2022-10-10

**Authors:** Haley Lescinsky, Ashkan Afshin, Charlie Ashbaugh, Catherine Bisignano, Michael Brauer, Giannina Ferrara, Simon I. Hay, Jiawei He, Vincent Iannucci, Laurie B. Marczak, Susan A. McLaughlin, Erin C. Mullany, Marie C. Parent, Audrey L. Serfes, Reed J. D. Sorensen, Aleksandr Y. Aravkin, Peng Zheng, Christopher J. L. Murray

**Affiliations:** 1 Institute for Health Metrics and Evaluation, University of Washington, Seattle, WA USA; 2 Department of Health Metrics Sciences, School of Medicine, University of Washington, Seattle, WA USA; 3 School of Population and Public Health, The University of British Columbia, Vancouver, British Columbia Canada; 4 Department of Applied Mathematics, University of Washington, Seattle, WA USA

**Keywords:** Risk factors, Cardiovascular diseases, Cancer

## Abstract

Characterizing the potential health effects of exposure to risk factors such as red meat consumption is essential to inform health policy and practice. Previous meta-analyses evaluating the effects of red meat intake have generated mixed findings and do not formally assess evidence strength. Here, we conducted a systematic review and implemented a meta-regression—relaxing conventional log-linearity assumptions and incorporating between-study heterogeneity—to evaluate the relationships between unprocessed red meat consumption and six potential health outcomes. We found weak evidence of association between unprocessed red meat consumption and colorectal cancer, breast cancer, type 2 diabetes and ischemic heart disease. Moreover, we found no evidence of an association between unprocessed red meat and ischemic stroke or hemorrhagic stroke. We also found that while risk for the six outcomes in our analysis combined was minimized at 0 g unprocessed red meat intake per day, the 95% uncertainty interval that incorporated between-study heterogeneity was very wide: from 0–200 g d^−1^. While there is some evidence that eating unprocessed red meat is associated with increased risk of disease incidence and mortality, it is weak and insufficient to make stronger or more conclusive recommendations. More rigorous, well-powered research is needed to better understand and quantify the relationship between consumption of unprocessed red meat and chronic disease.

## Abstract (web summary)

Using the burden of proof analytical tool, a meta-analysis found weak or no evidence of associations between unprocessed red meat consumption and increased risk of six cardiometabolic disease and cancer outcomes.

## Main

Previous research has broadly shown an association between red meat consumption and increased risks to human health^1–4^. The Global Burden of Diseases, Injuries and Risk Factors study (GBD) 2019 estimated that 896,000 (95% uncertainty interval (UI) 536,000–1,250,000) deaths and 23.9 million (15.6–32.0) disability-adjusted life years were attributable to unprocessed red meat consumption globally in 2019 (ref. ^5^). These and other findings have led institutions such as the World Health Organization, the World Cancer Research Fund (WCRF), the EAT-Lancet Commission and the US Departments of Health and Human Services and Agriculture to recommend limiting red meat intake^6–9^. Recommended consumption targets are inconsistent, however, ranging from 14 g d^−1^ (EAT^8^), to 50–70 g d^−1^ (WCRF^7^), to unrestricted amounts (Nutritional Recommendations Consortium^10^). Adding to this ambiguity, several studies have found no significant relationship between red meat consumption and risk of death^11,12^, which has led to further questioning of the strength of evidence in these risk pair associations^13,14^. To resolve conflicting data and recommendations, a quantitative and objective strategy for assessing the strength of the evidence relating red meat consumption to health outcomes is needed.

Evidence on the health effects of red meat consumption comes primarily from prospective observational cohort studies in which individuals are grouped into categories based on their level of red meat consumption^1,15^ and estimates of relative risks (RRs) comparing different levels of consumption are reported. Meta-analyses synthesize results from these studies either by pooling effect sizes that compare the extreme categories reported in each study, or by estimating the dose–response hazard ratio per unit of exposure—typically assuming a log-linear relationship—and pooling study-specific results^4,16^. These methods rely on a number of premises that may limit their utility to capture the effects of risk exposure on health outcomes. One issue involves the assumption of log-linearity, which requires that the hazard ratio for a fixed increment of red meat consumption (for example, 100 g d^−1^) remains constant across all levels of intake (an increase in consumption from 0 to 100 g d^−1^ would have the same effect as an increase from 200 to 300 g d^−^^1^). Yet evidence indicates that the dose–response relationship for many risk factors attenuates at higher doses^17,18^ (not log linear). Based on such evidence and in light of limited existing information on the shape of the risk curves for red meat and different health outcomes, it is plausible that the health effects of red meat consumption may not be well characterized by a log-linear function and should be investigated more closely. Another notable issue is that meta-analyses attempting to synthesize findings from cohort studies typically do not account for between-study heterogeneity, which can be a prominent source of bias in epidemiological meta-analyses^19^.

In this paper we examined the relationship between unprocessed red meat and six health outcomes: breast cancer, colorectal cancer, type 2 diabetes, ischemic heart disease (IHD), ischemic stroke and hemorrhagic stroke. These outcomes were selected using the WCRF criteria for convincing or probable evidence^7^. To rigorously quantify the dose–response relationship between unprocessed red meat consumption and the selected health outcomes—in addition to the strength of the evidence underlying the results—we systematically evaluated all available prospective epidemiological evidence on the association between unprocessed red meat consumption and each selected health outcome following the burden of proof risk function (BPRF) methodology developed by Zheng et al.^20^. Specifically, for each risk–outcome pair, we generated a mean risk function by implementing a flexible meta-regression framework that relaxed the conventional assumption of a log-linear dose–response relationship, instead using a data-driven approach to determine the shape of the function using a quadratic spline. The approach accounts for exposure ranges reported in the data and different comparator groups^21^. We automatically detected outliers using robust statistical trimming methods, tested and corrected for bias related to study design and evaluated publishing and reporting bias. We computed 95% UIs that account for mean effects uncertainty as well as between-study heterogeneity, adjusting for the number of studies. We used the information to calculate uncertainty inclusive of between-study heterogeneity to generate a BPRF^20^. The BPRF is a conservative risk function (offering an alternative to a mean risk function) that is defined as either the fifth (for harmful risks) or 95th (for protective risks) quantile curve closest to the line of RR equal to one (the null). Thus, the BPRF can be interpreted as the smallest harmful or protective effect at each level of exposure consistent with the available evidence.

We then converted the BPRF for each risk–outcome pair into a risk–outcome score (ROS)^20^. The ROS summarizes the magnitude and certainty of evidence about a risk or protective factor, with higher positive ROS always corresponding to a stronger relationship and negative ROS corresponding to a failure to reject the null. We further converted the ROS for each risk–outcome pair into a star rating: for both harmful and protective risks, an ROS of <0 received one star, >0–0.14 received two stars, >0.14–0.41 received three stars, >0.41–0.62 received four stars and >0.62 received five stars. Star ratings are designed to offer individuals and policy makers a way to understand the strength of evidence about a risk in a way that is comparable across risk–outcome pairs. The thresholds for each star rating were developed in consultation with collaborators, stakeholders and other audiences and can broadly be interpreted as indicating ‘no evidence of association,’ ‘weak evidence of association,’ ‘moderate evidence of association,’ ‘strong evidence of association’ and ‘very strong evidence of association.’ The main findings and implications for policy of this work are summarized in Table 1.

**Table 1:** Policy summary

Background	Characterizing the health effects of red meat intake is essential for making informed policy and diet recommendations. Previous meta-analyses on the effects of red meat have generated mixed findings and do not formally assess evidence strength.
Main findings and limitations	When between-study heterogeneity and other forms of uncertainty were incorporated into our analysis, unprocessed red meat was weakly associated with an increased risk of colorectal cancer, breast cancer, IHD and type 2 diabetes, by at least 6%, 3%, 1% and 1%, respectively. On a five-star scale with one star suggesting no evidence of association and five stars suggesting very strong evidence of association, these pairs each received two stars. Under this same interpretation of the data, there was no evidence of an association between unprocessed red meat and ischemic stroke or hemorrhagic stroke. These pairs received one star. RR for the six causes combined was minimized at 0 g d^−1^, but with wide uncertainty. Limitations of this study include sparse data of varied quality, potential for recall bias and other reporting or measurement errors in the data and difficulty with evaluating publication and reporting biases other than those related to the association of reported effect sizes and s.d. Further, for some risk–outcome pairs, there may be smaller ranges of exposure within which the magnitude of the association was larger and significant, but we chose to assign risk–outcome scores based on an average of a wide range of exposure levels (15th to 85th percentiles), which could obscure such effects.
Policy implications	The available evidence suggests that elevated unprocessed red meat has a weak association with the risk of colorectal cancer, breast cancer, IHD and type 2 diabetes. It may have an effect on ischemic stroke or hemorrhagic stroke, but there is currently insufficient evidence to draw this conclusion. The available evidence suggests that eating no unprocessed red meat may minimize the risk of disease incidence and mortality compared to consuming any, but there is insufficient evidence to make stronger or more conclusive recommendations. More rigorous, well-powered research is needed to better understand and quantify the relationship between unprocessed red meat intake and chronic disease.

## Results

### Overview

**Extended Data Fig. 1:**
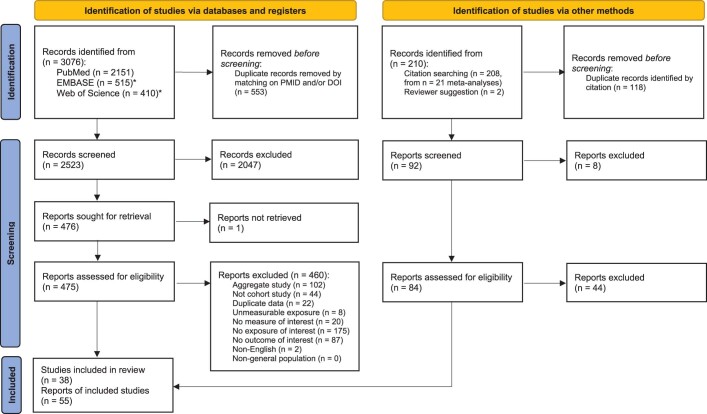
PRISMA flow diagram of unprocessed red meat data seeking approach. The PRISMA flow diagram covering unprocessed red meat and all 6 outcomes. Template is from: Page MJ, McKenzie JE, Bossuyt PM, Boutron I, Hoffmann TC, Mulrow CD, et al. The PRISMA 2020 statement: an updated guideline for reporting systematic reviews. BMJ 2021;372:n71. doi: 10.1136/bmj.n71. For more information, visit: http://www.prisma-statement.org/. * EMBASE and Web of Science were searched for recent records (from January 1, 2020, to May 10, 2022) to augment the date-unrestricted PubMed search (records up to May 10, 2022).

Following Preferred Reporting Items for Systematic Reviews and Meta-Analyses (PRISMA) guidelines^22^, we systematically searched for literature on the RR of unprocessed red meat consumption, screened identified records and extracted data from reports meeting our inclusion criteria (Methods). Of the 3,286 records identified, 55 reports^1,11,23–75^ met our inclusion criteria, providing information on 37 prospective cohorts and one nested case–control study (Extended Data Fig. 1). These included 18 cohorts and one nested case–control from Europe, 13 cohorts from North America, five cohorts from Asia and one from Australia. In most studies, dietary consumption of unprocessed red meat was assessed using food frequency questionnaires (42 of 55). The number of participants in each study ranged from 639 to 536,969 and the length of follow-up ranged from 4.1 to 32 years. In most studies (45 of 55), RRs were adjusted for major confounders including age, sex and smoking. Supplementary Table 1 (section 1) contains a summary of the main characteristics of all studies included in this analysis, including study design, end point, location, exposure assessment and other characteristics.

We found that removing trimming (including outliers) and removing the imposed model shape constraints did not significantly change the risk curve results. The Supplementary Information provides sensitivity results of these assumptions (section 2.1 and 2.2).

Risk–outcome scores; star ratings; risk curves with all data points, trimmed data points and conventional and conservative UIs; and an interpretation of the findings are available for all risk–outcome pairs at http://vizhub.healthdata.org/burden-of-proof/.

### Unprocessed red meat consumption and colorectal cancer

We identified 20 prospective cohorts^27,28,35,38,39,42,45,49,50,54,57,58,60,61,63,72,73,75^ and one nested case–control study^67^ to assess the relationship between unprocessed red meat consumption and colorectal cancer among 2,413,032 individuals in total (sample size was calculated as the number of unique data source-location pairs with observations of risk exposure and outcome) over an average of 8.0 years per individual (range of mean/median follow-up per cohort, 4.1–32 years). Ten cohorts were carried out in Europe, eight in North America, two in Asia and one in Australia. Overall, 16,202 colorectal cancer events were recorded across all cohorts, where 14,672 were incident cases and 1,530 were mortality cases. In addition to adjustments for major confounders including age, sex and smoking, the RRs in most cohorts were additionally adjusted for body mass index (BMI) (*n* = 14) and dietary components such as energy intake and fruit and vegetables (*n* = 16). Most cohorts reported the RR of colorectal cancer incidence (*n* = 18); no significant difference between incidence and mortality RRs was detected in covariate selection and no other bias covariates were identified by the covariate selection algorithm of the meta-regression.

**Fig. 1:**
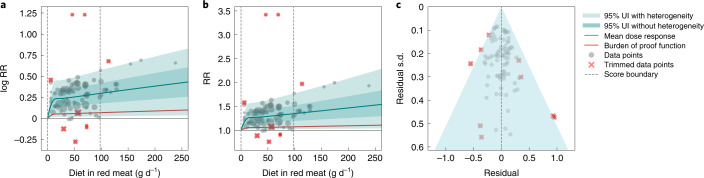
Unprocessed red meat consumption and colorectal cancer. **a**, Log-RR function. **b**, RR function. **c**, A modified funnel plot showing the residuals (relative to 0) on the *x* axis and the estimated s.d. that includes reported s.d. and between-study heterogeneity on the *y* axis.

We found weak evidence of harmful associations between unprocessed red meat consumption and risk of colorectal cancer; the mean RR at 50 g d^−1^ relative to no intake was 1.30 (95% UI inclusive of between-study heterogeneity of 1.01–1.64), while the mean RR at 100 g d^−1^ was 1.37 (1.01–1.78) (Table 2 and Fig. 1), where the UIs account for between-study heterogeneity and other forms of uncertainty. We estimated the exposure-averaged burden of proof RR to be 1.06, indicating that consuming unprocessed red meat in the range of 15th to 85th percentiles of exposure (0 g d^−1^ to 98 g d^−1^) was associated with at least a 6% higher risk of colorectal cancer. This corresponds to an ROS of 0.06 and a two-star rating, consistent with weak evidence.

**Table 2:** Strength of the evidence for the relationship between unprocessed red meat consumption and the six health outcomes analyzed

Health outcome	ROS	Average BPRF	Star rating	RR at 50 g d^−1^ (conservative 95% UI)	RR at 100 g d^−1^ (conservative 95% UI)
Colorectal cancer	0.06	1.06	2 stars	1.3 (1.01, 1.64)	1.37 (1.01, 1.78)
Breast cancer	0.03	1.03	2 stars	1.26 (0.98, 1.56)	1.26 (0.98, 1.56)
IHD	0.01	1.01	2 stars	1.09 (0.99, 1.18)	1.12 (0.99, 1.25)
Type 2 diabetes	0.01	1.01	2 stars	1.14 (0.97, 1.32)	1.23 (0.96, 1.52)
Ischemic stroke	−0.02	0.98	1 star	1.05 (0.97, 1.12)	1.15 (0.93, 1.4)
Hemorrhagic stroke	−0.13	1.14	1 star	0.9 (0.64, 1.26)	0.87 (0.56, 1.35)

The ROS represents the signed value of the log BPRF averaged across the 15th to 85th percentiles of exposure: the lower (if harmful) or higher (if protective) uncertainty interval—inclusive of between-study heterogeneity—for the RR curve for each risk–outcome pair. ROSs are directly comparable across outcomes and each risk–outcome pair receives an ROS based on the final formulation of the risk curve. For hemorrhagic stroke, the ROS reflects a protective effect of red meat consumption, whereas for the other outcomes it reflects a harmful effect. Negative ROSs indicate that a conservative interpretation of the available evidence suggests there may be no association between risk and outcome. For ease of interpretation, we have transformed the ROS and BPRF into a star rating (1–5), with a higher rating representing a larger effect and stronger evidence.

### Unprocessed red meat consumption and breast cancer

Six cohorts from Europe and four cohorts from North America were used to evaluate the association between unprocessed red meat intake and incidence of breast cancer^26,33,34,37,40,42,51,55,59,65^. The total number of participants across all cohorts was 999,428 individuals; the follow-up period ranged from 4.1 to 20.3 years (mean, 8.4 years); and the total number of incident cases was 25,732. In seven cohorts, the RR was adjusted for BMI and in eight cohorts, it was additionally adjusted for dietary components. All studies reported the RR of breast cancer incidence. There were no bias covariates identified by our algorithm as statistically relevant for inclusion in the model.

**Fig. 2:**
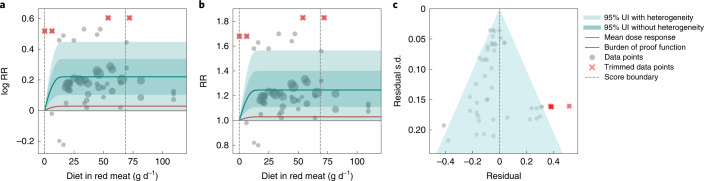
Unprocessed red meat consumption and breast cancer. **a**, Log-RR function. **b**, RR function. **c**, A modified funnel plot showing the residuals (relative to 0) on the *x* axis and the estimated s.d. that includes reported s.d. and between-study heterogeneity on the *y* axis.

We found weak evidence of a harmful association between unprocessed red meat intake and risk of breast cancer. The BPRF value (averaged across the 15th to 85th percentiles of red meat consumption, 0–69 g d^−1^) was 1.03, which was substantially lower than the mean RR of 1.26 (0.98–1.56) and 1.26 (0.98–1.56) at 50 g d^−1^ and 100 g d^−1^, respectively. The corresponding ROS is 0.03 (Table 2 and Fig. 2), which translates to a two-star risk and means that unprocessed red meat intake is associated with at least a 3% higher risk of colorectal cancer. When accounting for between-study heterogeneity, the mean RR UI at different exposure levels spanned 1 (Table 2).

### Unprocessed red meat consumption and ischemic heart disease

We included 11 prospective cohorts in the analysis of unprocessed red meat consumption and IHD^11,23,25,31,36,41,52,56,63,74^. The cohorts were from North America (*n* = 4), Europe (*n* = 4) and Asia (*n* = 3). The total number of participants across cohorts was 1,219,288 individuals and the mean duration of follow-up was 11.6 years (range, 5.5–30 years). Overall, 33,490 cases of IHD were recorded across all cohorts. These included 25,222 nonfatal cases, 3,959 fatal cases and 4,309 unspecified nonfatal/fatal cases. In most cohorts, the RRs were adjusted for BMI (*n* = 8) and dietary components (*n* = 10). Five studies reported the RR of IHD mortality, four reported for incidence and two reported a combination of incidence and mortality. We evaluated the effect of potential bias covariates, including an indicator that we assessed for differentiating between the RR of incidence and the RR of mortality and found that no bias covariates in our analysis had a significant effect on RR. We therefore did not include any in our model.

**Fig. 3:**
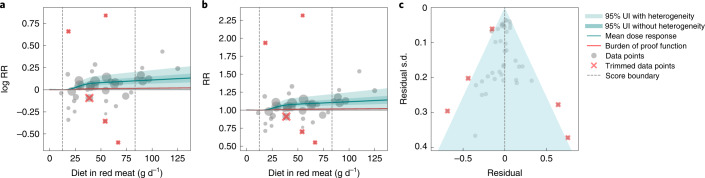
Unprocessed red meat consumption and ischemic heart disease. **a**, Log-RR function. **b**, RR function. **c**, A modified funnel plot showing the residuals (relative to 0) on the *x* axis and the estimated s.d. that includes reported s.d. and between-study heterogeneity on the *y* axis.

We found weak evidence of a harmful association between unprocessed red meat consumption and risk of IHD. The RR was 1.09 (0.99–1.18) at 50 g d^−1^ and 1.12 (0.99–1.25) at 100 g d^−1^ (Table 2 and Fig. 3). The corresponding exposure-averaged BPRF was 1.01, which translates to a ROS of 0.01 and a two-star rating at the lower threshold of two-star pairs (at the boundary between weak evidence and no evidence of an association between consumption of unprocessed red meat and increased risk of IHD incidence and mortality).

### Unprocessed red meat consumption and type 2 diabetes

We identified 17 prospective cohorts evaluating the relationship between unprocessed red meat consumption and type 2 diabetes among 1,619,574 participants^1,29,30,32,43,44,48,53,56,62,64,66,69–71^. Eight cohorts were from Europe, six from North America and three from Asia. Follow-up time ranged from 4.6 to 28 years (mean 11.6 years). Overall, 58,364 new cases of diabetes and 3,717 deaths from diabetes were recorded. In all cohorts but one, the RR was adjusted for BMI and in 15 cohorts, the RR was adjusted for dietary components. Only one cohort reported the RR of diabetes mortality and the rest reported incidence. No bias covariates were found to be statistically relevant and therefore none were included in the model.

**Extended Data Fig. 2:**
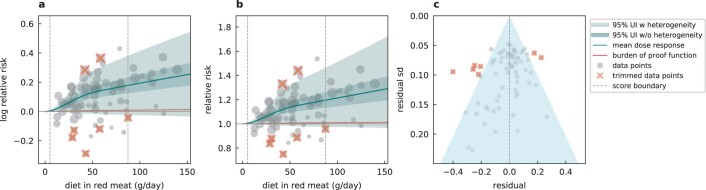
Red meat consumption and type 2 diabetes. **a**, log-relative risk function. **b**, relative risk function. **c**, A modified funnel plot showing the residuals (relative to 0) on the x-axis and the estimated standard deviation (SD) that includes reported SD and between-study heterogeneity on the y-axis.

We found evidence of weak harmful effects between unprocessed red meat consumption and risk of type 2 diabetes, with a mean RR of 1.14 (0.97–1.32) at 50 g d^−1^ relative to no intake and a mean RR of 1.23 (0.96–1.52) at 100 g d^−1^ relative to no intake (Table 2 and Extended Data Fig. 2). The BPRF value was 1.01 and the corresponding ROS was 0.01, equating to a two-star rating at the lower threshold of two-star pairs (at the boundary between weak evidence and no evidence of an association between consumption of unprocessed red meat and increased risk of type 2 diabetes).

### Unprocessed red meat consumption and ischemic stroke

The relationship between consumption of unprocessed red meat and ischemic stroke was assessed in eight prospective cohorts from Europe (*n* = 4), North America (*n* = 2) and Asia (*n* = 2)^24,46,47,56,63,68^.The number of participants across all cohorts was 1,185,969 individuals; the mean duration of follow-up was 11.4 years (range, 5.5–26 years); and the total number of events was 12,500 (11,996 nonfatal and 504 fatal cases). The RRs were adjusted for BMI (*n* = 6) in all but two cohorts and for dietary components in all cohorts (*n* = 8). Most studies reported the RR of ischemic stroke incidence (*n* = 6). No bias covariates were found to be statistically relevant and therefore none were included in the model.

**Extended Data Fig. 3:**
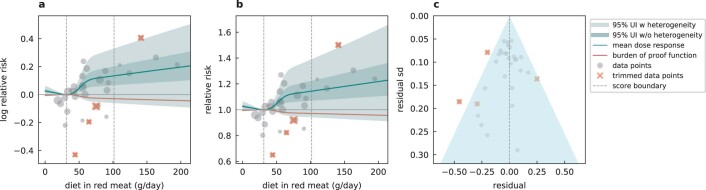
Red meat consumption and ischemic stroke. **a**, log-relative risk function. **b**, relative risk function. **c**, A modified funnel plot showing the residuals (relative to 0) on the x-axis and the estimated standard deviation (SD) that includes reported SD and between-study heterogeneity on the y-axis.

The exposure-averaged BPRF value for ischemic stroke (averaged between 15th and 85th percentiles of red meat exposure) was 0.98 (Table 2 and Extended Data Fig. 3), which put it opposite null from the mean RR of 1.15 (95% UI inclusive of between-study heterogeneity of 0.93–1.40) at 100 g d^−1^. The corresponding ROS of –0.02 resulted in a one-star rating, consistent with no evidence of an association between consumption of unprocessed red meat and increased risk of ischemic stroke.

### Unprocessed red meat consumption and hemorrhagic stroke

We evaluated the relationship between consumption of unprocessed red meat and hemorrhagic stroke using eight prospective cohorts from Europe (*n* = 4), North America (*n* = 2) and Asia (*n* = 2)^24,46,47,56,63,68^. The number of participants across all cohorts was 1,185,969 individuals the mean duration of follow-up was 11.4 years (range: 5.5–26 years); and the total number of events was 4,176 (3,646 nonfatal and 530 fatal cases). In addition to adjustments to the RRs for major confounders, all but two cohorts included adjustments for BMI (*n* = 6) and all were adjusted for dietary components (*n* = 8). More studies reported the RR of hemorrhagic stroke incidence (*n* = 6) than mortality. No bias covariates had a significant effect on RR, so none were included in the model.

**Extended Data Fig. 4:**
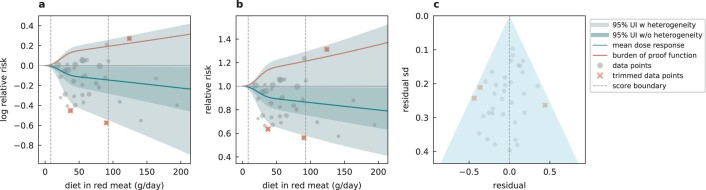
Red meat consumption and hemorrhagic stroke. **a**, log-relative risk function. **b**, relative risk function. **c**, A modified funnel plot showing the residuals (relative to 0) on the x-axis and the estimated standard deviation (SD) that includes reported SD and between-study heterogeneity on the y-axis.

The exposure-averaged BPRF value for hemorrhagic stroke was 1.14 (Table 2 and Extended Data Fig. 4), which was opposite null from the mean RR of 0.87 (0.56–1.35) at 100 g d^−1^. The corresponding ROS of −0.13 resulted in a one-star rating, consistent with no evidence of an association between consumption of unprocessed red meat and decreased risk of hemorrhagic stroke.

### Minimum risk level of unprocessed red meat intake

**Fig. 4:**
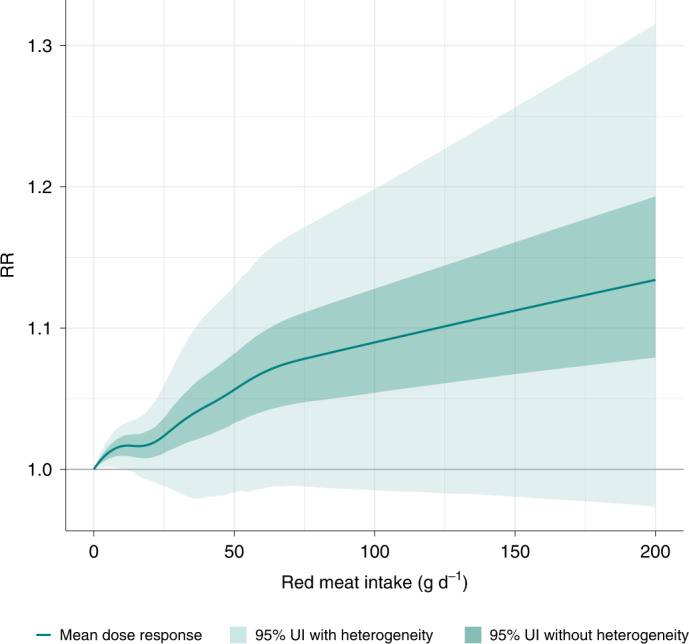
Aggregate RR curve for unprocessed red meat consumption and six health outcomes combined. The dark line indicates the combined-cause mean RR curve.

The aggregated risk curve for the six outcomes in our analysis combined was minimized at a mean unprocessed red meat consumption level of 0 g d^−1^ (95% UI 0–200) (Fig. 4). As shown in Fig. 4, the relationship between unprocessed red meat intake and combined-cause incidence and mortality was increasing across the entire exposure domain. Additional details on the results, along with sensitivity tests, are presented in Supplementary Information (section 2.3).

### Risk of bias assessment

We assessed potential publication and reporting bias using Egger’s regression and visual inspection of the funnel plots (Figs. 1c–3c and Extended Data Figs. 2c–4c). We found no evidence of publication or reporting bias in five of the six disease outcomes investigated, and mild evidence in ischemic stroke. Supplementary Information (section 3) contains detailed results for our assessment of study quality and risk of bias.

## Discussion

We evaluated the relationship between unprocessed red meat consumption and six selected disease outcomes following implementation of a meta-analytic approach^20^. We found that unprocessed red meat intake had weak evidence of an association with increased risk of colorectal cancer, breast cancer, IHD and type 2 diabetes and no evidence of an association with ischemic stroke and hemorrhagic stroke. In other words, given all the data available on red meat intake and risk of a subsequent outcome, we estimate that consuming unprocessed red meat across an average range of exposure levels increases the risk of subsequent colorectal cancer, breast cancer, IHD and type 2 diabetes at least slightly compared to eating no red meat (by at least 6%, 3%, 1% and 1%, respectively). Furthermore, the conservative interpretation of available data is consistent with no association between consuming unprocessed red meat and the risk of subsequent ischemic stroke or hemorrhagic stroke.

Based upon the star rating categories^20^, unprocessed red meat and colorectal cancer, breast cancer, IHD and type 2 diabetes are all two-star pairs, whereas the two stroke causes are one-star pairs. These star ratings reflect both the magnitude of the RR and its uncertainty and offer policy makers a simple way to conceptualize and compare the evidence for an association between risk and outcome. However, policy makers should pay attention to all potential risks, even those that only receive one-star or two-star ratings and especially when exposure prevalence is high. Further, the precautionary principle suggests that public policy should pay attention to all potential risks, as it is possible that as evidence accumulates, a stronger association may emerge for some pairs. Conversely, the results for IHD and type 2 diabetes are at the threshold between two-star and one-star ratings and further evidence may show that available data are consistent with no association between elevated unprocessed meat consumption and these outcomes. Because the current evidence for an association between unprocessed red meat and risk of these health outcomes is weak there is a critical need for better data from large-scale, high-quality studies in locations around the world. More evidence either in support of or against an association will allow policy makers to make better-informed decisions on diet recommendations.

A key finding of our analysis is that there is substantial between-study heterogeneity and uncertainty for all six risk–outcome pairs included. This may partly reflect the high degree of heterogeneity often present in data sources used for dietary analysis, which typically comprise observational studies. This heterogeneity limited the sensitivity of our analysis to identify clear—and potentially clinically important—relationships between intake and disease end points. Although visual inspection of the mean risk functions suggests a positive (harmful) relationship between unprocessed red meat intake and colorectal cancer, type 2 diabetes, IHD, ischemic stroke and breast cancer and a negative (protective) relationship with hemorrhagic stroke, the large degree of heterogeneity present, coupled with the moderate mean effects, generated wide UIs for the mean risk functions.

The level of red meat intake to optimize physical health is the subject of much interest and there is a wide range of recommendations in the literature^6–10^. By aggregating the outcome-specific risk curves computed in the present analysis, we generated a RR curve for the six outcomes combined that minimized risk at 0 g d^−1^ (95% UI 0–200) of unprocessed red meat consumption. This mean minimum risk is lower than the intake level recommended by the EAT-Lancet Commission (14 g d^−1^)^8^. Across the full range of exposures plotted on the combined-cause RR curve, we did not observe a significant relationship between unprocessed red meat consumption and combined-cause incidence and mortality (the 95% uncertainty interval is inclusive of 0). In light of these findings, we contend that consuming no unprocessed red meat likely minimizes the risk of health consequences compared to consuming any, but that the wide uncertainty and low star ratings prevent us from making a strong intake-level recommendation.

Previous meta-analyses have varied in their findings. With respect to colorectal cancer, the WCRF changed the grade of evidence for the relationship between red meat intake and colorectal cancer from possible to probable in 2017, reporting an RR of 1.12 (1.00–1.25)^76^. This estimate of RR is lower in magnitude than our result but was still found to be significant. For breast cancer, our finding of weak evidence of an association is generally consistent with previous literature, including a previous meta-analyses conducted by Farvid and colleagues (RR, 1.06; 0.99–1.14)^77^ and Anderson and colleagues (RR, 1.03; 0.99–1.08)^78^. Previous findings for IHD have been inconsistent, with Bechthold and colleagues finding an association between red meat intake and IHD^16^, but Abete and colleagues^79^ and Micha and colleagues^80^ reporting no association. Several meta-analyses examining the association between red meat consumption and type 2 diabetes identified associations of similar magnitude to our findings, though our UIs are wider; in 2017, Schwingshackl et al. reported an RR of 1.17 (1.08–1.26)^15^ and in 2011, Pan and colleagues found an RR of 1.19 (1.04–1.37)^1^. Although previous evidence on red meat and ischemic stroke has generally shown an association, which conflicts with our finding, the uncertainty intervals have been very close to the null. Yang et al.^81^ found that fresh (unprocessed) red meat consumption was associated with ischemic stroke (RR, 1.15; 1.03–1.29), as did Chen et al.^82^ (RR, 1.13; 1.01–1.25) and Kim et al.^83^ (1.24; 1.05–1.46) for total red meat. Any discrepancies between our findings and those in previous meta-analyses is primarily a reflection of our incorporation of between-study heterogeneity, not assuming a log-linear risk function and including newly available data. Our methods that included between-study heterogeneity in the uncertainty estimate revealed substantial uncertainty regarding the relationship between unprocessed red meat consumption and hemorrhagic stroke. Previous evidence regarding this relationship has been inconclusive. A 2019 study based on the EPIC Oxford cohort identified a higher rate of hemorrhagic stroke in vegetarians than in meat eaters, although this finding was not limited to red meat intake^83^. A meta-analysis by Yang and colleagues reported a pooled RR of 0.88 (0.73–1.06) for each 100 g d^−1^ increase in intake of unprocessed red meat^81^. Before that, meta-analyses by Chen and colleagues reported an RR of 0.99 (0.77–1.28)^82^ and Kaluza and colleagues reported an RR of 1.08 (0.84–1.39)^84^.

The effects of unprocessed red meat intake we observed vary over consumption levels. In particular, we observed high data inconsistency in evidence at low levels of red meat consumption, illustrating the importance of which foods serve as replacements for red meat. A systematic review of evidence from trials evaluating the effect of red meat on cardiovascular risk factors highlighted this replacement effect, showing that the harmful impact of red meat intake depends on the types of foods consumed in place of red meat^85^. At a lower level of intake, a wide range of healthy or unhealthy diet components might be substituted for red meat and could increase heterogeneity in the health effects of red meat across studies. We also observed a plateauing of the risk curves at high intake, indicating that above a certain threshold of intake, the risk levels off. Our ability to observe these non-linear relationships suggests that the associations between red meat intake and disease end points are better characterized by a flexible non-log-linear risk curve, allowing the observation of patterns that were potentially obscured in previous research by reliance on log-linear risk curves.

Our analysis has several strengths. We present an approach to dose–response risk curve estimation that uses exposure ranges from cohort studies to infer flexible risk functions without imposing log-linear assumptions. The approach quantifies between-study heterogeneity and uses this information to distinguish risk–outcome pairs with strong evidence from those with weak evidence to inform policies and guidance on unprocessed red meat intake. To explain some of the between-study heterogeneity, the approach uses bias covariates automatically selected from an expert-defined candidate set consistent with GRADE^86^, Cochrane^87^ and other evidence-grading criteria. Application of this analysis method to other diet components may yield insights into the shape of risk functions and burden of proof risk functions for a variety of dietary factors. Further, our analysis method and findings are compatible with GBD analyses of prevalence of exposure and background rates of disease outcomes. Collectively, the GBD comparative risk assessment framework allows for the evaluation of the importance of risk–outcome pairs across the full range of risk-attributable burdens and star ratings. For instance, a risk factor with high prevalence of exposure and a two-star relationship with a common and serious health outcome might warrant more policy focus than a risk factor with lower prevalence of exposure and a three-star or four-star relationship with a rare or less severe health outcome. The star rating for a risk–outcome pair is just one component to consider when making policy recommendations, but its compatibility with GBD estimates adds value to our approach.

Although our meta-analysis approach presents an analytic framework to quantify and account for a wide range of source data characteristics that may have obscured aspects of risk–outcome relationships investigated in previous studies, there were a number of limitations related to our source data, our methodological approach and our ability to interpret the data that we were unable to address. First, we did not identify any randomized controlled trials that evaluate the relationship between red meat intake and chronic diseases among adults; therefore, all of the studies included in our analysis were observational and we were unable to definitively assess causality. Second, while many of the sources we included adjusted to some degree for major confounders such as age, sex, smoking, certain other dietary components, cooking method, socioeconomic status and regional dietary patterns, the level and rigor of adjustment varied across studies, leaving the potential for residual confounding. Third, the definition of exposure and unit of exposure were not identical across studies and consumption of red meat was self-reported (and in most studies assessed only at baseline), raising the potential for recall bias and other reporting and/or measurement errors that may have resulted in exposure misclassification. Fourth, we assessed and summarized the risk of bias for each study but included all data points in our model instead of limiting to those scoring ‘low risk of bias’ on standard assessments because of the low availability of data. Fifth, the data came primarily from North America, Europe and Asia, which potentially limits the interpretability and application of our findings to locations outside of these regions. Sixth, although research suggests that red meat intake may be crucially related to infant and child growth and development^88,89^, this analysis evaluated the effect of red meat consumption on selected chronic disease end points in adults only and did not attempt to address questions of development in children and adolescents. Seventh, our trimming approach requires that the threshold for outliers be user-specified. Our sensitivity analyses demonstrate that fitting 90% of the data was most appropriate, but automation of this process would strengthen the approach. Eighth, types of publication and reporting biases other than those related to the association of reported effect sizes and s.d. are difficult to evaluate within our methodological framework. This is particularly true when studies are more consistent with each other than expected by chance^20^. Ninth, we used Gaussian priors on the bias covariates to avoid overfitting, which meant that relationships were only identified when there were sufficient studies supporting the estimate of the bias. Using alternative priors might change the biases that were detected. Finally, we chose to calculate an exposure-averaged BPRF and ROS based on a wide range of exposure levels (15th to 85th percentiles). For some pairs, particularly for the one-star pairs, there may be smaller ranges of exposure within which the magnitude of the BPRF was larger and significant, indicating a significant association between risk and outcome at a certain level of exposure. Picking an ‘average’ range of exposure was necessary for assigning each pair a single star rating and we believe a single rating is more useful from a policy perspective because of its simplicity, but this added level of complexity may be worth considering in subsequent analyses.

In conclusion, we applied the BPRF framework of Zheng et al.^20^ to present a systematic meta-analysis of red meat consumption on six important health outcomes. Our analysis found a high degree of between-study heterogeneity and uncertainty in the existing body of evidence on the health effects of red meat intake. When all forms of uncertainty including between-study heterogeneity were incorporated, we found weak evidence of associations between unprocessed red meat consumption and colorectal cancer, breast cancer, IHD and type 2 diabetes. The available data were consistent with no association between unprocessed red meat consumption and ischemic stroke and hemorrhagic stroke, which received one-star ratings. While there is some evidence that eating red meat increases risk of chronic disease, there is insufficient evidence to make stronger or more conclusive recommendations. More rigorous, well-powered research is needed to better understand and quantify the relationship between red meat consumption and chronic disease.

## Methods

### Overview

The analytical approach (described previously^20^) can be summarized in six steps: (1) search and extract data from published studies using a standardized approach; (2) estimate the shape of the exposure versus RR relationship, integrating over exposure ranges in different comparison groups and avoiding the distorting effect of outliers; (3) test and adjust for systematic biases as a function of study attributes; (4) quantify remaining between-study heterogeneity while adjusting for within-study correlation induced by computing RRs for several alternatives with the same reference, as well as the number of studies; (5) evaluate evidence for small-study effects to evaluate potential risks of publication or reporting bias; and (6) estimate the BPRF—quantifying a conservative interpretation of the change in average risk across the range of exposure supported by the evidence—using this estimate to compute the ROS and map it onto a star-rating system, stratifying risk into five levels. Zheng and colleagues^21^ previously published the technical developments required to implement this approach and disseminated them using open-source Python libraries^90,91^.

The estimates for our primary indicators from this work—RRs across a range of exposures, BPRFs, ROSs and star ratings for each risk–outcome pair—are not specific to, or disaggregated by, specific populations (we did not estimate by location, sex or age group; though this analysis evaluated the effects of unprocessed red meat consumption on selected chronic disease end points in adults 25 years and older only and breast cancer is only applicable to females). The reports we referenced included information about the self-reported sex of the participants but did not all include sex-specific RR estimates; also, studies were excluded if they did not meet our minimum threshold for confounder adjustment of adjusting for age and sex. These factors precluded us from performing any sex-based analyses. The measures of risk can be considered current until subsequent analyses are made based on newly available data.

We followed the PRISMA guidelines^22^ through all stages of this study (Extended Data Fig. 1 and Supplementary Tables 3 and 4). This study complies with the Guidelines on Accurate and Transparent Health Estimate Reporting recommendations^92^ (Supplementary Table 5). This study was approved by the University of Washington Institutional Review Board Committee (study 9060). The systematic review was not registered.

### Selecting health outcomes

We selected outcomes on the basis of the availability of epidemiological evidence on their potential relationship with red meat. As detailed by Murray et al.^5^, risk–outcome pairs were initially selected using the WCRF criteria for convincing or probable evidence. Guidance from WCRF states that probable (strong) evidence is ‘strong enough to support a judgment of a probable causal (or protective) relationship.’^7^ A probable association generally requires evidence from at least two independent cohort studies, no substantial unexplained between-study heterogeneity, good-quality studies and evidence of biological plausibility.

After evaluating published literature on the relationship between red meat and various disease end points, we found sufficient studies assessing the relationship between red meat consumption and six outcomes: incidence of and mortality due to hemorrhagic stroke, type 2 diabetes, colorectal cancer, IHD and ischemic stroke and incidence of breast cancer. Supplementary Information, section 5, provides more details on the outcomes for which data were sought.

### Conducting systematic reviews

We systematically searched PubMed for reports of cohort studies that included meat consumption, selecting reports evaluating the relationship between consumption of red meat and each of the outcomes. These literature searches were last performed on 10 May 2022. To ensure that we were capturing the most recent literature, we also searched Embase and Web of Science for reports published within the past 2 years, as well as the citation lists of recent systematic reviews on health effects of red meat^1,15,16,77–83,93–103^ for relevant original investigations. Titles and abstracts of all identified articles were manually screened by one investigator. A second investigator reviewed a random sample of 20% of excluded reports for potential discrepancies; no discrepancies were found. Full texts of potentially relevant articles were manually assessed for eligibility by two investigators. Supplementary Information, section 5.1, contains the full search string.

We defined red meat consumption as total consumption of unprocessed red meat, including beef, lamb and pork, excluding processed meat. Reports involving processed meat were excluded because we aimed to distinguish the health effects of red meat intake per se from the health effects of meat preservatives or preservation byproducts. We also excluded reports that did not report RRs or only reported RR estimates that were unadjusted for key confounders such as age and sex. When duplicate publications from the same study were identified, we only included the report that included the longest duration of follow-up in person-years.

We defined outcomes using the most highly specified diagnosis possible. For stroke outcomes, we excluded data points on total stroke from the hemorrhagic- and ischemic-specific models, as we found that including total stroke data points obscured the unique relationships between red meat and the two types of stroke for which we had sufficient data to model separately. Similarly, for IHD, we excluded RRs from incidence or mortality of unspecified cardiovascular disease outcomes and limited our model to RRs specifically associated with IHD outcomes to most accurately assess the relationship with IHD. Supplementary Information, section 5, contains a full list of inclusion and exclusion criteria. Data from 55 total reports met inclusion criteria for at least one of the six outcomes and were included in the risk–outcome pair-specific analysis^1,11,23–75^.

Data extraction was manually conducted by two investigators. For each study, we extracted data on study name, location, design, population (age, sex, race and sample size), duration of follow-up, exposure definition, exposure assessment method, exposure categories, outcome definition, outcome ascertainment method and specific confounders that were included in the adjusted effect size. For each exposure category, we also collected data on the range of exposure, number of participants, person-years, number of events and risk estimate and associated uncertainty. Supplementary Table 6 contains a full list of extracted variables. We standardized the exposure unit to grams of consumption per day. For reports describing consumption in servings of red meat with no corresponding information about serving size, we assumed a serving size of 85 g d^−1^ (ref. ^104^). For reports that gave mean consumption rather than intake range, we calculated the range by using the midpoint between means to provide a cutoff for intake intervals. For undefined lower bounds, we assumed a consumption level of 0 g d^−1^. For undefined upper bounds when mean and s.d. values were not available, we applied the range from the cohort’s most adjacent quartile or tertile to estimate the upper bound of consumption, specific to each study cohort.

### Estimating the shape of the risk–outcome relationship

Following Zheng et al.^20^, we modeled each non-linear dose–response as a quadratic spline^21^. For each risk–outcome pair, we first modeled the non-linear RR function without a monotonicity constraint to observe the unconstrained behavior. For outcomes in which the mean curve remained above one across the whole domain and was generally increasing, we then fitted a final model applying a monotonicity constraint to ensure that the mean risk curve was non-decreasing. For outcomes in which the curve decreased then increased and was minimized at a non-zero value, we did not apply a monotonicity constraint but instead implemented a linear-tail constraint (sometimes referred to as ‘natural’ splines in curve-fitting literature) on the left side of the domain to ensure more plausible risk curve behavior at low exposure levels. Linear-tail constraints ensure that the final segment on one side of the domain is linear, rather than a quadratic or cubic binomial and are a common way to regularize spline behavior. We present the sensitivity of the results based on this assumption in the Supplementary Information, section 2.1.

Because knot placement can affect the shape of the risk function when modeling with a spline, we generated a wide range of knot placements and created an ensemble across 50 component models, weighted by their fit to the data and the smoothness of fit to the observations. We also included in the final estimation 10% trimming of the data to avoid sensitivity to outliers; trimming from within the likelihood is an efficient way to identify outliers without manually selecting them^21,105^. Through this process, we generated a mean RR curve across the range of exposures (a measure of effect size) for each red meat–outcome pair. We obtained uncertainty estimates for the mean risk curves using a parametric bootstrap approach. Details on the fitting procedure, including trimming, ensemble generation and posterior uncertainty estimation, can be found in Zheng et al.^20^.

### Testing for bias across different study designs and characteristics

To capture bias within the studies and data points, we followed the approach of Zheng et al.^20^, using a Lasso^106,107^ covariate selection scheme to rank the potential bias covariates. We then converted the dose–response relationship previously estimated by non-linear meta-regression into a new ‘signal’ covariate and used linear meta-regression to systematically test for significant modification of the ‘signal’ by each bias covariate—adding them to the model one at a time based on the Lasso ranking. Significant bias covariates were then included in the spline models; the details of this procedure have been published elsewhere^20^. Supplementary Information, section 3 provides more information on our risk of bias assessment.

### Quantifying between-study heterogeneity, accounting for heterogeneity, uncertainty and small numbers of studies

The variance of between-study random effects is notoriously difficult to estimate. We quantified between-study heterogeneity (the level of disagreement in the inferred relationship between risk and outcome found in each study) by scaling the non-linear RR based on each study using a study-specific random slope with respect to the new bias covariate model. For cases with small numbers of studies, the estimated between-study heterogeneity can often be zero. To safeguard against underestimating the between-study heterogeneity when too few studies were available, we used the Fisher information matrix^108^ to estimate the variance of the between-study heterogeneity, Zheng et al.^20^ provides more details. Our main results include the effect of between-study heterogeneity in UIs, although we also present UIs that do not include it (as is standard in conventional meta-regressions) in Supplementary Table 7.

### Evaluating potential for publication or reporting bias

To assess potential publication or reporting bias, we used a data-driven approach known as Egger’s regression^109^ to test for significant correlation between the residuals of the risk function and their s.d. When Egger’s regression failed to detect significant evidence of publication bias, we terminated the process.

When significant bias was detected, we adjusted for it using an appropriate modification of the trim-and-fill algorithm^110^. The trimming process in extracting the signal covariate is robust both to outliers and to classic cases of publication bias. Given a robust estimate of the mean curve, we applied the ‘fill’ part of the trim-and-fill approach, using the rank statistics and residuals to compute the number of points that need to be ‘filled’. The correct number of the most extreme residuals was then reflected and might affect our resulting estimate of heterogeneity. When fitting for the updated between-study heterogeneity, we used a prior coming from the original fit, with s.d. obtained from the Fisher information.

In addition to this statistical test of publication or reporting bias, we generated funnel plots of the residuals of the risk function and s.d. and inspected them visually. In the presence of significant publication bias, we used the trim-and-fill method to adjust for bias^20,110^.

### Determining minimum risk consumption level

To determine the minimum risk level of red meat consumption, we aggregated the outcome-specific risk curves to generate an aggregated mortality curve for the six causes in our analysis combined, which we used to identify the exposure range that minimizes risk. Specifically, we took a weighted average of the risk curve draws for each outcome, using the GBD 2019 burden of each outcome—with deaths as the metric—as weights^5,111^. Then we calculated the exposure level that minimized combined-cause mortality for each draw and reported the median and associated 95% UI.

### Estimating the burden of proof risk function

Using estimates of the mean risk function and corresponding 95% UIs—inclusive of between-study heterogeneity and reflective of other bias adjustments—as described in the preceding sections, we calculated a BPRF for each risk–outcome pair. Specifically, for γ = between-study heterogeneity, we let γ_0.95_ represent the 95% quantile according to the asymptotic distribution obtained using Fisher information. We computed the lower envelope of the log-RR curve that included fixed effects uncertainty as well as γ_0.95_ and found the average value of this curve at specific exposures and along the specified ranges of exposure. The BPRF is defined as the fifth (if harmful) or 95th (if protective) quantile risk curve—inclusive of between-study heterogeneity—closest to the RR equal to one (the null). The BPRF reflects a conservative interpretation of the available evidence and is a measure of the lowest level of excess risk (or risk reduction, for protective risks) that is consistent with the available data; the higher the BPRF, the higher the magnitude and strength of the relationship. This value can be interpreted such that even accounting for between-study heterogeneity and its uncertainty, we are confident that the log RR across the studied unprocessed meat consumption range is at least as high as the BPRF (or at least as low as the BPRF for a protective risk).

We then calculated ROSs as the signed average log RR of the BPRF over the 15th to 85th percentiles of observed exposure^20^. For example, a mean log BPRF of 0.4 for a harmful risk (where null = 0 for log RR) and a mean log BPRF of –0.4 for a protective risk would both have an ROS of 0.4 because the magnitude of the log RR is the same. In contrast, for risk–outcome pairs with a BPRF opposite the null from the mean risk, ROS would be calculated as negative. A positive risk score indicates that across average levels of exposure, the UI bound that is closest to null is on the same side of null as the mean risk curve. Positive ROSs indicate evidence of a risk–outcome relationship. Overall, a larger positive ROS indicates more consistency in evidence and a higher average effect size across the continuum of the risk. As an estimate of strength of evidence, the ROS is also directly comparable across outcomes, so we can use it to rank disease outcomes by confidence in their relationship to red meat consumption.

Finally, to aid interpretability of our findings for policy makers and research funders, we converted the ROSs to star-rating categories from one to five, with a higher rating indicating that the evidence for and magnitude of a relationship between risk and outcome is stronger. The conservative interpretation suggests that, for one-star pairs, there may be no true association between risk exposure and health outcome. As noted, we further divided the positive ROSs into ranges of at least a 0–15% increase (for harmful risks) or 0–13% decrease (for protective risks) in risk with average exposure (two stars), >15–50% increase or >13–34% decrease (three stars), >50–85% increase or >34–46% decrease (four stars) and greater than 85% increase or greater than 46% decrease (five stars). In ROS terms, the ranges are <0.0, 0.0–0.14, >0.14–0.41, >0.41–0.62 and >0.62 for both harmful and protective risks.

### Model validation

We used detailed simulations to validate key components of the meta-regression model. The details of these model validations are available in Zheng et al.^20^. In brief, we simulated three scenarios: many studies with many data points per study, many studies with few data points per study and few studies with few data points per study. For each simulation, we compared the results from our model with results obtained using existing approaches, including a log-linear meta-analysis implemented in the metafor package^112^ as well as one-stage^113^ and two-stage^114^ competing approaches using the dosresmeta package^115^. For log-linear relationships, our approach and the metafor package showed similarly superior performance over the one- and two-stage approaches, whereas for non-log-linear relationships, our approach produced uniformly better performance across all scenarios compared to the other approaches.

### Statistical analysis

Analyses were carried out using R v.3.6.1, Python v.3.8 and Stata v.17. To validate key aspects of the meta-regression model used in this analysis, the following packages were used, as described by Zheng et al. metafor (R package available for download at https://www.jstatsoft.org/article/view/v036i03) and dosmesreta (R package available for download at https://www.jstatsoft.org/article/view/v072c01).

### Statistics and reproducibility

The study was a secondary analysis of existing data involving systematic reviews and meta-analyses. No statistical method was used to predetermine sample size. As the study did not involve primary data collection, randomization, blinding and data exclusions are not relevant to this study; and, as such no data were excluded and we performed no randomization or blinding. We have made our data and code available to foster reproducibility.

### Reporting summary

Further information on research design is available in the Nature Research Reporting Summary linked to this article.

## Online content

Any methods, additional references, Nature Research reporting summaries, source data, extended data, supplementary information, acknowledgements, peer review information; details of author contributions and competing interests; and statements of data and code availability are available at 10.1038/s41591-022-01968-z.

## Supplementary information

Supplementary Information Supplementary Sections 1–7, Supplementary Tables 1–8 and Supplementary Figs. 1–8

Reporting Summary

## Data Availability

The findings from this study were produced using data available in the published literature. Data sources and citations for each risk–outcome pair can be downloaded using the ‘download’ button on each risk curve page available at https://vizhub.healthdata.org/burden-of-proof/. Study characteristics and citations for all input data used in the analyses are also provided in Supplementary Table 1, a template of the data collection form is in Supplementary Table 6 and the results from individual studies are in Supplementary Table 8.

## Code availability

All code used for these analyses is publicly available online (https://github.com/ihmeuw-msca/burden-of-proof). This includes code for the meta-regression engine, the model specification interface, both parts of the data processing and risk-specific custom code, as appropriate.
