# Supplementary material for: Health effects associated with consumption of unprocessed red meat: a Burden of Proof study
Source: Nat Med. 2022 Oct 10;28(10):2075–82. doi: 10.1038/s41591-022-01968-z (PMC9556326; doi:10.1038/s41591-022-01968-z)
Supplement: Supplementary file 1 — Supplementary Sections 1–7, Supplementary Tables 1–8 and Supplementary Figs. 1–8 [file 41591_2022_1968_MOESM1_ESM.pdf]

nature portfolio

https://doi.org/10.1038/s41591-022-01968-z

Supplementary information

Health effects associated with consumption of unprocessed red meat: a Burden of Proof study

In the format provided by the authors and unedited

Supplementary Information: supplementary methods, data sources, and results for "Health effects associated with consumption of unprocessed red meat: a Burden of Proof study"

This appendix provides detailed information on input data sources and presents supplementary results—particularly sensitivity analysis results—for "Health effects associated with consumption of unprocessed red meat: a Burden of Proof study."

Table of Contents

SECTION 1: STUDY CHARACTERISTICS 3
Supplementary Table 1. Study characteristics 4
SECTION 2: SENSITIVITY RESULTS 12
Section 2.1: Model constraints 12
Supplementary Figure 1a-f. The relative risk of all six outcomes for different values of red meat consumption, in grams/day, no shape constraints 13
Section 2.2: Results without trimming 15
Supplementary Figure 2a-f. The relative risk of all six outcomes for different values of red meat consumption, in grams/day, without trimming any data points 15
Section 2.3: Minimum risk level 16
Supplementary Figure 3. The mean of the across-cause risk curve, relative to 0, as the sum of the additive components........ 17
Supplementary Figure 4. The distribution of minimum-risk exposure draws for the across-cause relative risk curve........ 17
Supplementary Figure 5. Aggregate across-cause relative risk curve for red meat consumption, dalys weighting........ 18
Supplementary Figure 6. Aggregate across-cause relative risk curve for red meat consumption, only two-star outcomes........ 18
Supplementary Figure 7. The mean of the across-cause relative risk curve, relative to 0, as the sum of the additive components, only 2-star outcomes 19
Supplementary Figure 8. The distribution of minimum-risk exposure draws for the across-cause risk curve, only 2-star outcomes 20
SECTION 3: STUDY QUALITY AND RISK OF BIAS ASSESSMENT 20
Supplementary Table 2. Study quality for every study used in the models 20
SECTION 4. GATHER AND PRISMA CHECKLISTS........ 23
Supplementary Table 3. PRISMA 2020 checklist........ 23
Supplementary Table 4. PRISMA 2020 abstract checklist 27
Supplementary Table 5. GATHER checklist 28
SECTION 5: DATA SOURCE IDENTIFICATION AND ASSESSMENT 31
Section 5.1: Literature identification........ 31
Section 5.2: Assessing data source eligibility 33
Supplementary Table 6. Causal criteria extraction template........ 33
SECTION 6: RISK CURVE DETAILS 39
Supplementary Table 7. Relative risks across exposure range........ 39
SECTION 7: RESULTS FROM INDIVIDUAL STUDIES 40
Supplementary Table 8. Summary results from input studies........ 40

Section 1: Study characteristics

In supplementary table 1 we provide characteristics for each of the studies identified and used in this meta-analysis. Some cohorts (ex: the EPIC cohort) were the basis of data for multiple reports spanning multiple outcomes. Here we are explicit about the reporting source for each study and which outcomes it is linked to. In total, we used data from 38 different studies that spanned 55 unique reports. Key study characteristics like population, location, study design, sex, follow up (in years), age, exposure assessment tool, disease ascertainment tool, person-years, events (incident or fatal cases), and sample size are presented. Wherever possible we used the sample size and person-year values as reported in the publications, but calculated person-years as sample size*follow-up when not otherwise reported. We sought data for fatal (mortality) and nonfatal (incidence) outcomes for each of the six health outcomes being considered (hemorrhagic stroke, type 2 diabetes, colorectal cancer, ischemic heart disease, breast cancer, and ischemic stroke), at different levels of red meat exposure. For breast cancer, we only identified input data for incidence.

Supplementary Table 1. Study characteristics for all included studies

Supplementary Table 1. Study characteristics

Outcome	Report	Study name	Population	Location	Study design	Sex	Follow-up	Age start	Age end	Exposure assessment	Endpoint	Disease ascertainment	Person-years	Events	Sample size
Breast cancer	Diallo 2018	NutriNet-Sante Study	Participants with access to the Internet are continuously recruited among the general population by means of vast multimedia campaigns	France	Prospective cohort	both	4.1	35+		FFQ	Incidence	Administrative medical records or disease registries	229835	544	61476
Breast cancer	Genkinger 2013	Black Women's Health Study	recruited from subscribers to Essence Magazine, members of professional organisations, and friends/relatives of early respondents	United States	Prospective cohort	women	12	21	69	FFQ	Incidence	Administrative medical records or disease registries	497733	1093	59027
Breast cancer	Gilsing 2016	Netherlands Cohort Study	The NLCS Meat Investigation Cohort, an analytical cohort embedded in the ongoing prospective Netherlands Cohort Study	Netherlands	Prospective cohort	women	20.3	55	69	FFQ	Incidence	Administrative medical records or disease registries	90627	312	5218
Breast cancer	Inoue-Choi 2016	NIH-AARP Diet and Health Study	AARP members from six states (California, Florida, Louisiana, New Jersey, North Carolina, and Pennsylvania) and two metropolitan areas (Atlanta, Georgia; and Detroit, Michigan)	United States	Prospective cohort	women	9.4	50	71	FFQ	Incidence	Administrative medical records or disease registries	1821175	9306	193742
Breast cancer	Kabat 2007	Canadian National Breast Screening Study	Canadian women ages 40 to 59	Canada	Prospective cohort	women	16.4	40	59	FFQ	Incidence	Administrative medical records or disease registries	798024	2491	48660
Breast cancer	Knuppel 2020	UK BIOBANK	UK volunteers	United Kingdom	Prospective cohort	women	6.9	37	73	diet questionnaire	Incidence	Administrative medical records or disease registries	1743830.1	5612	252729
Breast cancer	Mills 1989	Adventist Health Study	Seventh Day Adventist women	California	Prospective cohort	women	6	25+		Surveillance program	Incidence	Administrative medical records or disease registries	115000	134	20341
Breast cancer	Pala 2009	European Prospective Investigation into Cancer and Nutrition (EPIC)	Women from 23 centers from 10 countries. In most centers, participants came from the general population. However, the French cohort was recruited from female members of a health insurance	Denmark, France, Germany, Greece, Italy, Norway, Spain, Sweden, the Netherlands, and the UK	Prospective cohort	women	8.8	25	70	FFQ	Incidence	Administrative medical records or disease registries	2812610	5372	319826

Supplementary Table 1. Study characteristics for all included studies

			scheme for school and university employees, the Turin and Ragusa (Italy) and the Spanish centers included blood donors, participants in Utrecht were recruited from a mammographic screening program, the Florence cohort included screening program participants, and half the Oxford cohort consisted of "health conscious" persons from England, Wales, Scotland, and Northern Ireland												
Breast cancer	Pouchieu 2014	Supplementation en Vitamines et Mineraux Antioxydants study	women enrolled in the double-blind, placebo-controlled, randomised trial	France	Prospective cohort	women	11.3	mean (sd)	48(6.4)	24DR	Incidence	Self-report	52943	190	4684
Breast cancer	Taylor 2007	UK Women's Cohort Study	500 000 responders to a direct mail survey of the World Cancer Research Fund (WCRF). Seventy-five percent of the responders agreed to take part in a more detailed survey	United Kingdom	Prospective cohort	women	8	35	69	FFQ	Incidence	Administrative medical records or disease registries	269800	678	33725
Colon and rectum cancer	Al Rajabi 2022	Alberta's Tomorrow Project	Albertans aged 35-69 years, with no history of cancer except non-melanoma skin cancer	Canada	Prospective cohort	both	13.4	35	69	diet history questionnaire	Incidence	Administrative medical records or disease registries	351321.2	257	26218
Colon and rectum cancer	Egeberg 2013	Danish, Diet, Cancer and Health	People living in the greater Copenhagen and Aarhus areas	Denmark	Prospective cohort	both	13.4	50	64	FFQ	Incidence	Administrative medical records or disease registries	2153715	989	160725
Colon and rectum cancer	English 2004	Melbourne Collaborative Cohort Study	Residents from Melbourne, Australia including Italian and Greek immigrants	Australia	Prospective cohort	both	9	40	69	FFQ	Incidence	Administrative medical records or disease registries	334008	451	37112
Colon and rectum cancer	Gilsing 2015	Netherlands Cohort Study	People originating from 204 municipalities with computerised population registries	Netherlands	Prospective cohort	both	20.3	55	69	FFQ	Incidence	Administrative medical records or disease registries	141875	437	311567
Colon and rectum cancer	Jarvinen 2001	Finnish Mobile Clinic Health Examination Survey	General population	Finland	Prospective cohort	both	32	18+		FFQ	Incidence	Administrative medical records or disease registries	1998080	109	62440
Colon and rectum cancer	Jones 2019	Iowa Women's Health Study	Women randomly selected from Iowa driver's license records	Iowa, United States	Prospective cohort	women	24	55	69	FFQ	Incidence	Administrative medical records	1666080	1649	69420

Supplementary Table 1. Study characteristics for all included studies

												or disease registries			
Colon and rectum cancer	Knuppel 2020	UK BIOBANK	UK volunteers	United Kingdom	Prospective cohort	both	6.9	37	73	diet questionnaire	Incidence	Administrative medical records or disease registries	3231539.1	3201	468339
Colon and rectum cancer	Larsson 2005	Swedish Mammography Cohort	Women residing in Uppsala and Vastmanland counties in central Sweden	Sweden	Prospective cohort	women	13.9	40	75	FFQ	Incidence	Administrative medical records or disease registries	855585	733	61433
Colon and rectum cancer	Mehta 2020	Sister Study	Women with no history of breast cancer, but have had a sister diagnosed with breast cancer. United states and Puerto Rico	United States	Prospective cohort	women	4.8	35	74	FFQ	Incidence	Administrative medical records or disease registries	420574	216	48704
Colon and rectum cancer	Mejborn 2020	Danish National Survey on Diet and Physical Activity	Invited individuals were randomly drawn from the Danish Civil Registration System and comprised non-institutionalised free-living Danish citizens	Denmark	Prospective cohort	both	8.7	15	75	Food diary	Incidence	Administrative medical records or disease registries	54653	127	6282
Colon and rectum cancer	Ollberding 2012	The Multiethnic Cohort Study	Adults residing in California and Hawaii comprised of African Americans, Japanese Americans, Latinos, Native Hawaiians, and whites	United States	Prospective cohort	both	13.6	45	75	FFQ	Incidence	Death certificates	165717	3404	165717
Colon and rectum cancer	Parr 2013	Norwegian Women and Cancer (NOWAC) cohort study	national, population-based cohort	Norway	Prospective cohort	women	11.1	41	70	FFQ	Incidence	Administrative medical records or disease registries	820419	666	95906
Colon and rectum cancer	Pietinen 1999	Alpha-Tocopherol, Beta-Carotene Cancer Prevention study	Male smokers from southwestern Finland	Finland	Prospective cohort	men	8	50	69	diet questionnaire	Incidence	Administrative medical records or disease registries	216888	185	27111
Colon and rectum cancer	Singh 1998	Adventist Health Study	non-Hispanic white California Seventh-day Adventists and others living in Adventist households	California, United States	Prospective cohort	both	6	25+		diet questionnaire	Incidence	Administrative medical records or disease registries	163858	127	32051
Colon and rectum cancer	Takata 2013	Shanghai Men's Health study	Men residing in urban areas of Shanghai	Shanghai	Prospective cohort	men	5.5	40	74	FFQ	Mortality	Administrative medical records or disease registries	334281	261	61128
Colon and rectum cancer	Takata 2013	Shanghai Women's Health Study	Women residing in urban areas of Shanghai	Shanghai	Prospective cohort	women	11.2	40	70	FFQ	Mortality	Administrative medical records or disease registries	803265	261	73162

Supplementary Table 1. Study characteristics for all included studies

Colon and rectum cancer	Tiermersma 2002	Monitoring Project on Cardiovascular Disease Risk Factors	People from 3 Dutch towns: Amsterdam, Maastricht, and Doetinchem	Netherlands	Nested case-control	both	8.5	20	59	FFQ	Incidence	Administrative medical records or disease registries	5431.5	102	639
Colon and rectum cancer	Ward 2016	European Prospective Investigation into Cancer and Nutrition (EPIC)	volunteers from 23 centres in ten countries (Sweden, Denmark, Norway, The Netherlands, UK, France, Germany, Spain, Italy and Greece)	Sweden, Denmark, Norway, The Netherlands, UK, France, Germany, Spain, Italy and Greece	Prospective cohort	both	4.1	25	70	FFQ	Mortality	Administrative medical records or disease registries	2131910	1008	519978
Colon and rectum cancer	Wei 2004	Health Professionals Follow-Up Study	US male health professionals	United States	Prospective cohort	men	14	40	75	FFQ	Incidence	Administrative medical records or disease registries	652848	582	46632
Colon and rectum cancer	Wei 2004	Nurses' Health Study	US female registered nurses	United States	Prospective cohort	women	20	30	55	FFQ	Incidence	Administrative medical records or disease registries	1754660	873	87733
Colon and rectum cancer	Yiannakou 2022	Black Women's Health Study	US Black women volunteers, most of whom were subscibers to Essence magazine	United States	Prospective cohort	women	22	21	69	FFQ	Incidence	Administrative medical records or disease registries	1116176	564	50735.2727
Diabetes mellitus type 2	Ericson 2015	MDC study	living in the city of Malmo¨ were invited to participate	Sweden	Prospective cohort	both	14	45	74	diet history interview	Incidence	Administrative medical records or disease registries	24070	2860	26930
Diabetes mellitus type 2	Etemadi 2017	NIH-AARP Diet and Health Study	AARP members from six states (California, Florida, Louisiana, New Jersey, North Carolina, and Pennsylvania) and two metropolitan areas (Atlanta, Georgia; and Detroit, Michigan)	United States	Prospective cohort	both	16	50	71	diet history questionnaire	Mortality	Administrative medical records or disease registries	7540835	3717	536969
Diabetes mellitus type 2	Fretts 2012	Strong Heart Family study	13 American Indian communities in Arizona, North Dakota, South Dakota, and Oklahoma	United States	Prospective cohort	both	8	35	74	FFQ	Incidence	Biomarker	16008	243	2001
Diabetes mellitus type 2	InterAct Consortium 2013	European Prospective Investigation into Cancer and Nutrition (EPIC)	collaboration between European countries (Denmark, France, Germany, Italy, the Netherlands, Spain, Sweden, UK).	Denmark, France, Germany, Italy, the Netherlands, Spain, Sweden, UK	Case-cohort	both	11.7	20	80	FFQ	Incidence	Administrative medical records or disease registries	305229.6	11559	26088
Diabetes mellitus type 2	Kurotani 2013	Japan Public Health Center-based Prospective study	five Japanese public health centre areas (Iwate, Akita, Nagano, Okinawa and Tokyo) and public health centre	Japan	Prospective cohort	both	5	40	69	FFQ	Incidence	Self-report	319245	1178	63849

Supplementary Table 1. Study characteristics for all included studies

			areas (Ibaraki, Niigata, Kochi, Nagasaki, Okinawa and Osaka)												
Diabetes mellitus type 2	Lajous 2012	French E3N cohort	middle-aged French women responded to a mailed reproductive, lifestyle, and medical questionnaire	France	Prospective cohort	women	13.8	40	65	FFQ	Incidence	Self-report	838097	1369	66118
Diabetes mellitus type 2	Mannisto 2010	Alpha-Tocopherol, Beta-Carotene Cancer Prevention study	Male smokers living in Southwestern Finland	Finland	Prospective cohort	men	12	50	69	FFQ	Incidence	Administrative medical records or disease registries	311316	1098	25943
Diabetes mellitus type 2	Montonen 2005	Finnish Mobile Clinic Health Examination Survey	general population	Finland	Prospective cohort	both	23	40	69	FFQ	Incidence	Administrative medical records or disease registries	98992	52	4304
Diabetes mellitus type 2	Pan 2011	Health Professionals Follow-Up Study	US male health professionals	United States	Prospective cohort	men	20	40	75	FFQ	Incidence	Self-report	652974	2438	37083
Diabetes mellitus type 2	Pan 2011	Nurses' Health Study	US female registered nurses	United States	Prospective cohort	women	28	30	55	FFQ	Incidence	Self-report	2014172	8253	79570
Diabetes mellitus type 2	Pan 2011	Nurses' Health Study II	US female registered nurses	United States	Prospective cohort	women	16	25	42	FFQ	Incidence	Self-report	1366175	3068	87504
Diabetes mellitus type 2	Papier 2021	UK BIOBANK	UK volunteers, with no history of cancer except non-melanoma skin cancer	United Kingdom	Prospective cohort	both	8	37	73	24DR	Incidence	Administrative medical records or disease registries	3546040	9595	443255
Diabetes mellitus type 2	Steinbrecher 2011	The Multiethnic Cohort Study	Caucasians, Japanese-Americans, and Native Hawaiians in Hawaii	Hawaii	Prospective cohort	both	13.5	45	75	FFQ	Incidence	Administrative medical records or disease registries	913005	8587	75512
Diabetes mellitus type 2	Talaei 2017	Singapore Chinese health study	descendants from southern China, either from the Fujian province, where the Hokkien dialect is spoken, or from the Guangdong province, where the Cantonese dialect is spoken; both are major dialects common among Chinese in Singapore	Singapore	Prospective cohort	both	7	45	74	FFQ	Incidence	Self-report	494741	5207	63257
Diabetes mellitus type 2	Villegas 2006	Shanghai Women's Health Study	Women residing in urban areas of Shanghai	Shanghai	Prospective cohort	women	4.6	40	70	FFQ	Incidence	Self-report	326581	1969	74493
Diabetes mellitus type 2	Virtanen 2017	Kuopio Ischaemic Heart Disease Risk Factor Study	Men from eastern Finland	Finland	Prospective cohort	men	19.3	42	60	guided food record	Incidence	Physician diagnosis	44906.984	432	2332

Supplementary Table 1. Study characteristics for all included studies

Diabetes mellitus type 2	van Woudenbergh 2012	Rottersdam study	study among inhabitants of Ommoord, a district of the city of Rotterdam, the Netherlands	Netherlands	Prospective cohort	both	12.4	55+		FFQ	Incidence	Physician diagnosis	47941	456	4366
Hemorrhagic stroke	Bernstein 2012	Health Professionals Follow-Up Study	US male health professionals	United States	Prospective cohort	men	22	40	75	FFQ	Incidence	Administrative medical records or disease registries	833660	217	43150
Hemorrhagic stroke	Bernstein 2012	Nurses' Health Study	US female registered nurses	United States	Prospective cohort	women	26	30	55	FFQ	Incidence	Administrative medical records or disease registries	2041679	475	84010
Hemorrhagic stroke	Larsson 2011	Cohort of Swedish Men	men from 2 counties in Sweden	Sweden	Prospective cohort	men	10.1	45	79	FFQ	Incidence	Administrative medical records or disease registries	407641	350	40291
Hemorrhagic stroke	Larsson 2011	Swedish Mammography Cohort	Women residing in Vastmanland County and in 1988–1990 in Uppsala County in central Sweden	Sweden	Prospective cohort	women	10.4	39	70	FFQ	Incidence	Administrative medical records or disease registries	359013	233	34670
Hemorrhagic stroke	Papier 2021	UK BIOBANK	UK volunteers, with no history of cancer except non-melanoma skin cancer	United Kingdom	Prospective cohort	both	8	37	73	24DR	Incidence	Administrative medical records or disease registries	3449832	941	431229
Hemorrhagic stroke	Takata 2013	Shanghai Men's Health study	Men residing in urban areas of Shanghai	Shanghai	Prospective cohort	men	5.5	40	74	FFQ	Mortality	Administrative medical records or disease registries	334281	212	61128
Hemorrhagic stroke	Takata 2013	Shanghai Women's Health Study	Women residing in urban areas of Shanghai	Shanghai	Prospective cohort	women	11.2	40	70	FFQ	Mortality	Administrative medical records or disease registries	803265	318	73162
Hemorrhagic stroke	Tong 2020	European Prospective Investigation into Cancer and Nutrition (EPIC)	volunteers from 22 centres in nine European countries	Denmark, Germany, Greece, Italy, the Netherlands, Norway, Spain, Sweden, and the UK	Prospective cohort	both	12.7	35	70	diet questionnaires, primarily FFQ	Incidence	Administrative medical records or disease registries	5312778.3	1430	418329
Ischemic heart disease	Al-Shaar 2020	Health Professionals Follow-Up Study	US male health professionals	United States	Prospective cohort	men	30	40	75	FFQ	Incidence	Administrative medical records or disease registries	1023872	4456	43272
Ischemic heart disease	Bernstein 2010	Nurses' Health Study	US female registered nurses	United States	Prospective cohort	women	26	30	55	FFQ	Incidence & mortality	Administrative medical records or disease registries	2050071	3162	84136
Ischemic heart disease	Fraser* 1999	Adventist Health Study	non-Hispanic white California Seventh-day Adventists and others living in Adventist households	California, United States	Prospective cohort	both	6	25+		surveillance program	Mortality	Administrative medical records or disease registries	180000	2716	34198

Supplementary Table 1. Study characteristics for all included studies

Ischemic heart disease	Haring 2014	Atherosclerosis Risk in Communities Study (ARIC)	community-based prospective cohort study of middle-aged adults from four US communities (Washington County, Md; Forsyth County, NC; Jackson, Miss; and suburbs of Minneapolis, Minn.)	United States	Prospective cohort	both	22	45	64	FFQ	Incidence & mortality	Death certificates	233688	1147	12066
Ischemic heart disease	Key 2019	European Prospective Investigation into Cancer and Nutrition (EPIC)	volunteers (mostly ages 25–70 years) from 23 centres in ten countries (Sweden, Denmark, Norway, The Netherlands, UK, France, Germany, Spain, Italy and Greece)	Sweden, Denmark, Norway, The Netherlands, UK, France, Germany, Spain, Italy and Greece	Prospective cohort	both	12.6	mean(sd)	52(10)	FFQ	Incidence	Administrative medical records or disease registries	5164551	7193	409885
Ischemic heart disease	Möller 2021	Danish National Survey on Diet and Physical Activity	Non-institutionalised Danish citizens without IHD at baseline	Denmark	Prospective cohort	both	9.8	15	75	Food diary	Incidence	Administrative medical records or disease registries	77214.51	439	8007
Ischemic heart disease	Nagao 2012	Japan Collaborative Cohort Study	People enrolled from 45 communities across Japan	Japan	Prospective cohort	both	18.4	40	79	FFQ	Mortality	Death certificates	820075	537	51683
Ischemic heart disease	Papier 2021	UK BIOBANK	UK volunteers, with no history of cancer except non-melanoma skin cancer	United Kingdom	Prospective cohort	both	8	37	73	24DR	Incidence	Administrative medical records or disease registries	3449832	13134	431229
Ischemic heart disease	Takata 2013	Shanghai Men's Health study	Men residing in urban areas of Shanghai	Shanghai	Prospective cohort	men	5.5	40	74	FFQ	Mortality	Administrative medical records or disease registries	334281	306	61128
Ischemic heart disease	Takata 2013	Shanghai Women's Health Study	Women residing in urban areas of Shanghai	Shanghai	Prospective cohort	women	11.2	40	70	FFQ	Mortality	Administrative medical records or disease registries	803265	306	73162
Ischemic heart disease	Whiteman 1999	OXCHECK Study	Patients registered with five urban practices around Luton and Dunstable (Bedfordshire, UK)	United Kingdom	Prospective cohort	both	9	35	64	FFQ	Mortality	Administrative medical records or disease registries	5586	94	10522
Ischemic stroke	Bernstein 2012	Health Professionals Follow-Up Study	US male health professionals	United States	Prospective cohort	men	22	40	75	FFQ	Incidence	Administrative medical records or disease registries	833660	829	43150
Ischemic stroke	Bernstein 2012	Nurses' Health Study	US female registered nurses	United States	Prospective cohort	women	26	30	55	FFQ	Incidence	Administrative medical records or disease registries	2041679	1383	84010
Ischemic stroke	Larsson 2011	Cohort of Swedish Men	men from 2 counties in Sweden	Sweden	Prospective cohort	men	10.1	45	79	FFQ	Incidence	Administrative medical records or disease registries	407641	1849	40291

Supplementary Table 1. Study characteristics for all included studies

Ischemic stroke	Larsson 2011	Swedish Mammography Cohort	Women residing in Vastmanland County and in 1988–1990 in Uppsala County in central Sweden	Sweden	Prospective cohort	women	10.4	39	70	FFQ	Incidence	Administrative medical records or disease registries	359013	1310	34670
Ischemic stroke	Papier 2021	UK BIOBANK	UK volunteers, with no history of cancer except non-melanoma skin cancer	United Kingdom	Prospective cohort	both	8	37	73	24DR	Incidence	Administrative medical records or disease registries	3449832	2344	431229
Ischemic stroke	Takata 2013	Shanghai Men's Health study	Men residing in urban areas of Shanghai	Shanghai	Prospective cohort	men	5.5	40	74	FFQ	Mortality	Administrative medical records or disease registries	334281	184	61128
Ischemic stroke	Takata 2013	Shanghai Women's Health Study	Women residing in urban areas of Shanghai	Shanghai	Prospective cohort	women	11.2	40	70	FFQ	Mortality	Administrative medical records or disease registries	803265	320	73162
Ischemic stroke	Tong 2020	European Prospective Investigation into Cancer and Nutrition (EPIC)	volunteers from 22 centres in nine European countries (Denmark, Germany, Greece, Italy, the Netherlands, Norway, Spain, Sweden, and the UK)	Global	Prospective cohort	both	12.7	35	70	diet questionnaires, primarily FFQ	Incidence	Administrative medical records or disease registries	5312778.3	4281	418329

Section 2: Sensitivity results

Section 2.1: Model constraints

For each risk–outcome pair, we first modelled the non-linear curve without a monotonicity constraint to observe the behavior without any shape constraints. For outcomes in which the mean curve remained above 1 across the whole domain and was generally increasing, we then fit a final model applying a monotonicity constraint to ensure that the mean risk curve was non-decreasing. For outcomes in which the curve decreased then increased and was minimized at a non-zero value (j-shape like), we did not apply a monotonicity constraint but instead implemented a linear-tail constraint on the left side of the domain to ensure more plausible risk curve behavior at low exposure levels.

Specifically, the monotonic outcomes are modelled with quadratic splines, 2 interior knots, right linear tails, and a prior on the maximum derivative of the right linear tail (mean = 0, sd = 0.001). The non-monotonic outcomes are modelled with quadratic splines, 3 interior knots, right and left linear tails, and priors on the maximum derivative of both tails (mean = 0, sd = 0.001).

Below we present results of the model without any shape constraints from the first step. These models are fit with quadratic splines, 3 interior knots, left and right linear tails, and a prior on the maximum derivative of the right linear tail (mean = 0, sd = 0.001). We use a left linear tail (sometimes called a `natural spline') to improve stability, ensuring that the risk curve at low exposure levels is informed by multiple low exposure data points, rather than potentially driven only by the lowest exposure data point.

Supplementary Figure 1a-f. The relative risk of all six outcomes for different values of red meat consumption, in grams/day, no shape constraints

These risk curves are modeled with quadratic splines, 3 interior knots, a left linear tail and a right linear tail (max derivative prior of mean = 0, sd = 0.001). There is no monotonicity constraint. Following the decision criteria outlined above, hemorrhagic stroke, colorectal cancer, breast cancer, and diabetes were modeled as monotonic outcomes. IHD and ischemic stroke were modeled as J shape risks for the main result risk curves.

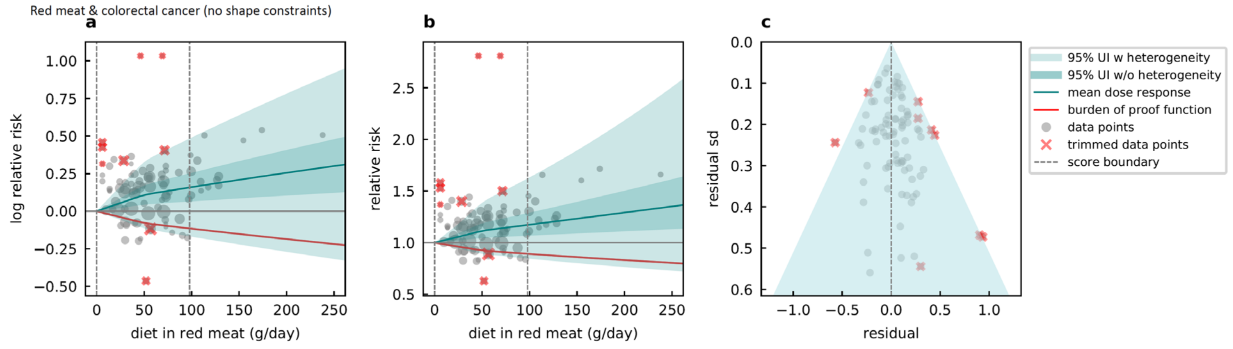

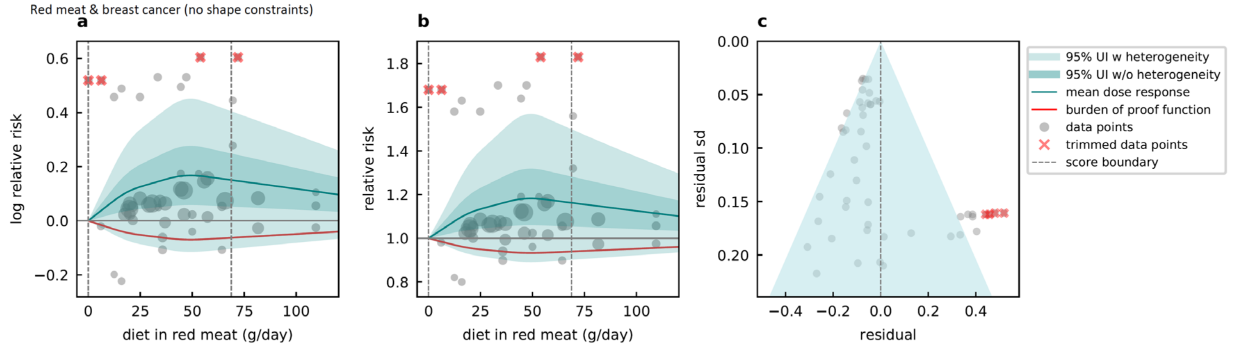

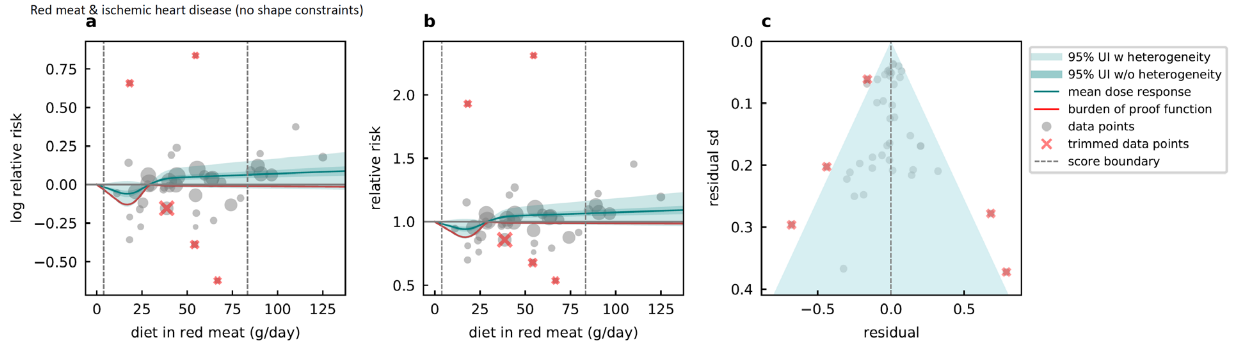

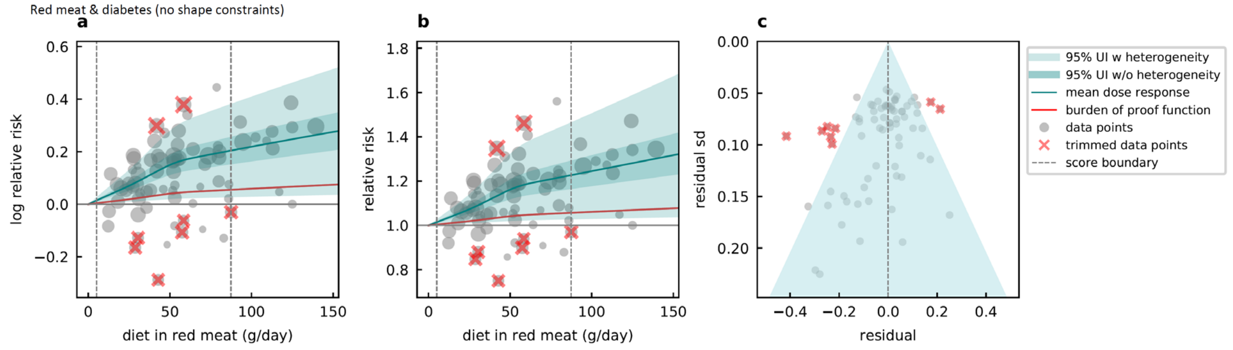

Red meat & ischemic stroke (no shape constraints)

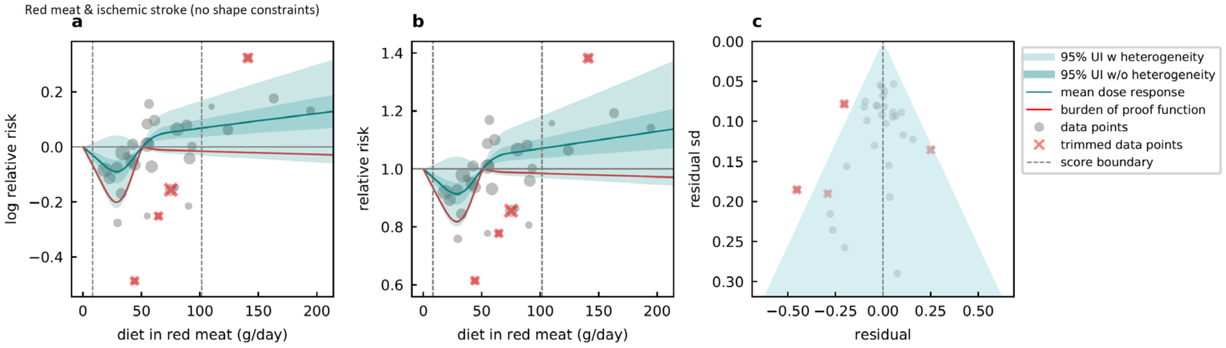

Red meat & hemorrhagic stroke (no shape constraints)

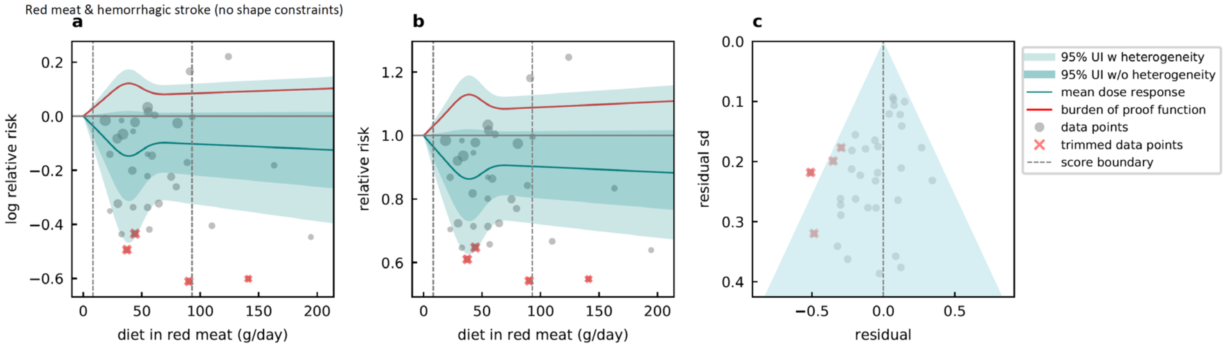

Section 2.2: Results without trimming

Supplementary Figure 2a-f. The relative risk of all six outcomes for different values of red meat consumption, in grams/day, without trimming any data points

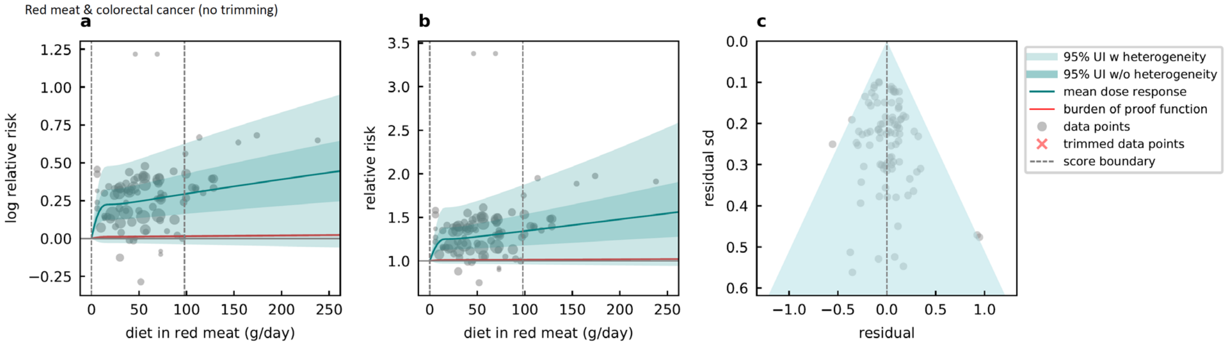

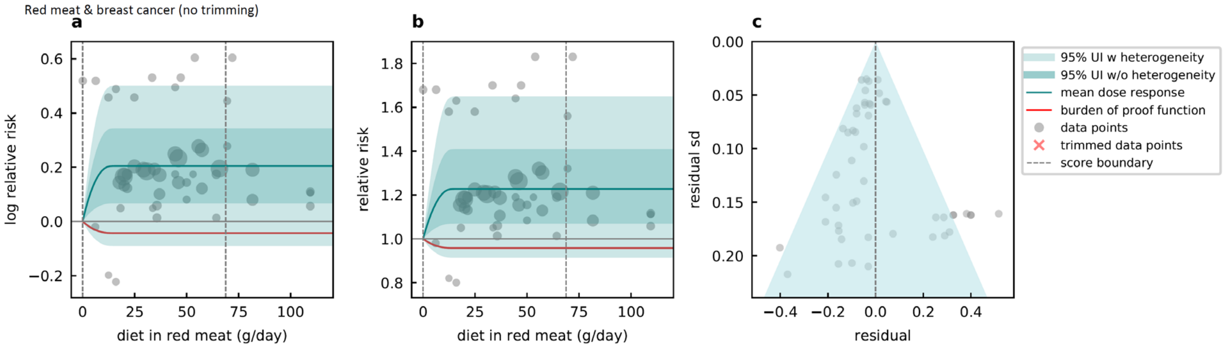

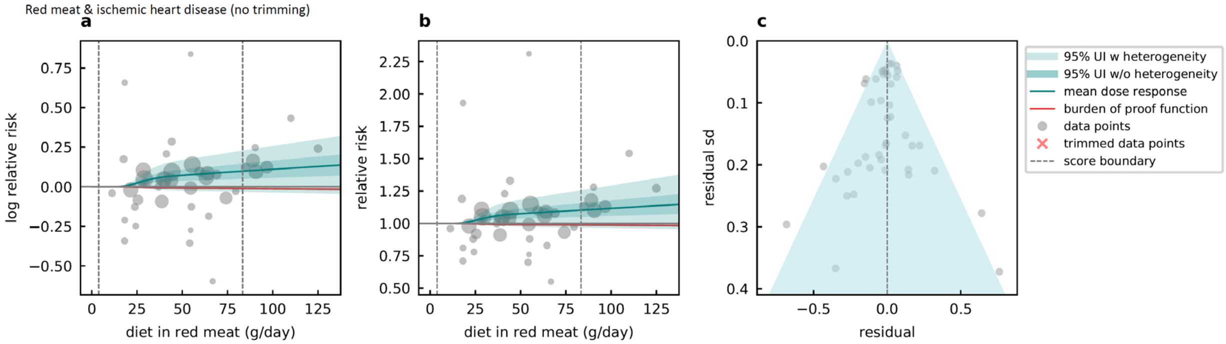

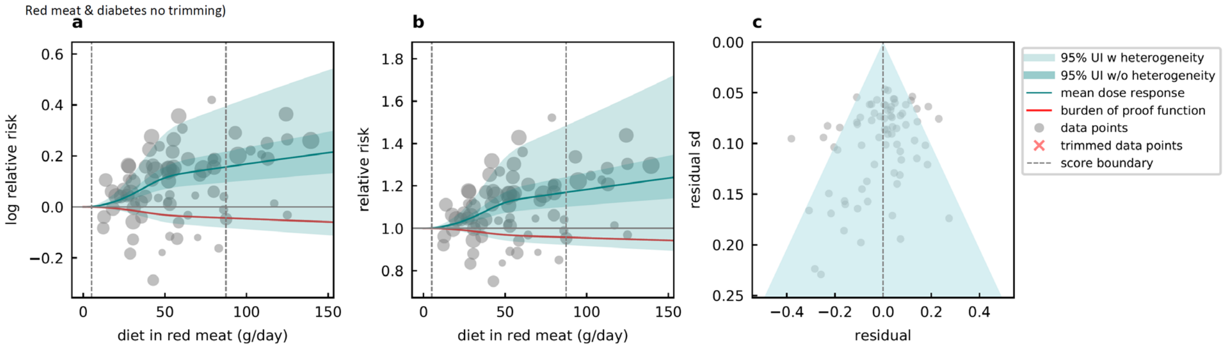

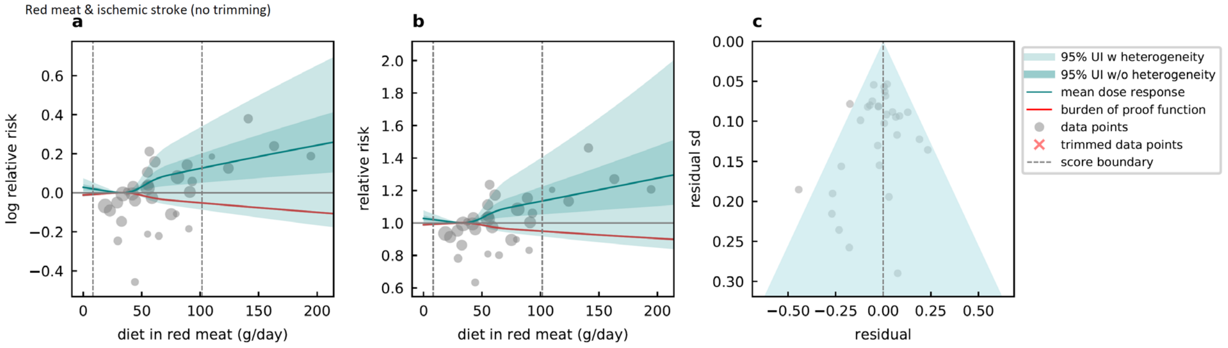

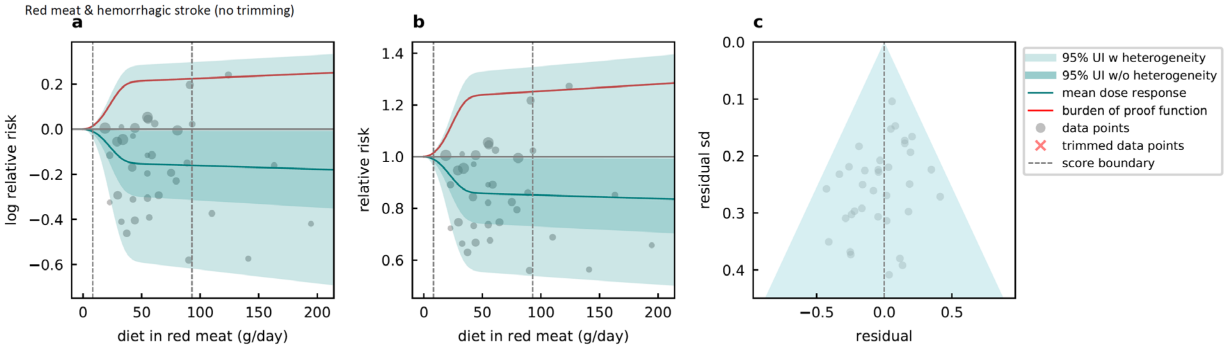

Section 2.3: Minimum risk level

Section 2.3.1: Main analysis method

To determine the minimum-risk exposure level, we first took a weighted average of the outcome-specific risk curves, using the GBD 2019 global mortality burden as weights. Then we used the resulting aggregate across-cause-risk curve to identify the exposure values that minimize the risk of disease incidence and mortality posed by the 6 outcomes in this analysis. Supplementary Figure 3, below, illustrates how the across-cause risk curve is influenced by the relative contribution of each outcome-specific curve.

Figure 4 in the main text visualizes the aggregate across-cause risk curve with and without between-study-heterogeneity. To get the minimum-risk exposure level, we take the consumption level that minimizes each draw of the relative risk and report the median and 95% confidence interval of the distribution. Supplementary Figure 4 shows the distribution of draws of the minimum-risk exposure level. We note that draws are not normally distributed; specifically 4% of the aggregate risk curve draws are monotonically decreasing and are therefore are minimized at the highest consumption level evaluated (200 g/day in this scenario). Further, since the distribution is skewed, the mean across-cause risk curve was minimized at the median instead of the mean. Therefore, we chose to summarize the results with the median and uncertainty interval, rather than the mean.

Supplementary Figure 3. The mean of the across-cause risk curve, relative to 0, as the sum of the additive components

Specifically, the across-cause curve line (red) is represented with mean relative risk on the Y and exposure (consumption in g/day) on the X axis. It is calculated as the direct sum of each of the 6 outcome-specific risk curves, which are presented with the Y-axis as relative risk * weight (breast cancer 4%, colorectal cancer 6%, IHD 48%, hemorrhagic stroke 17%, ischemic stroke 17%, diabetes 8%).

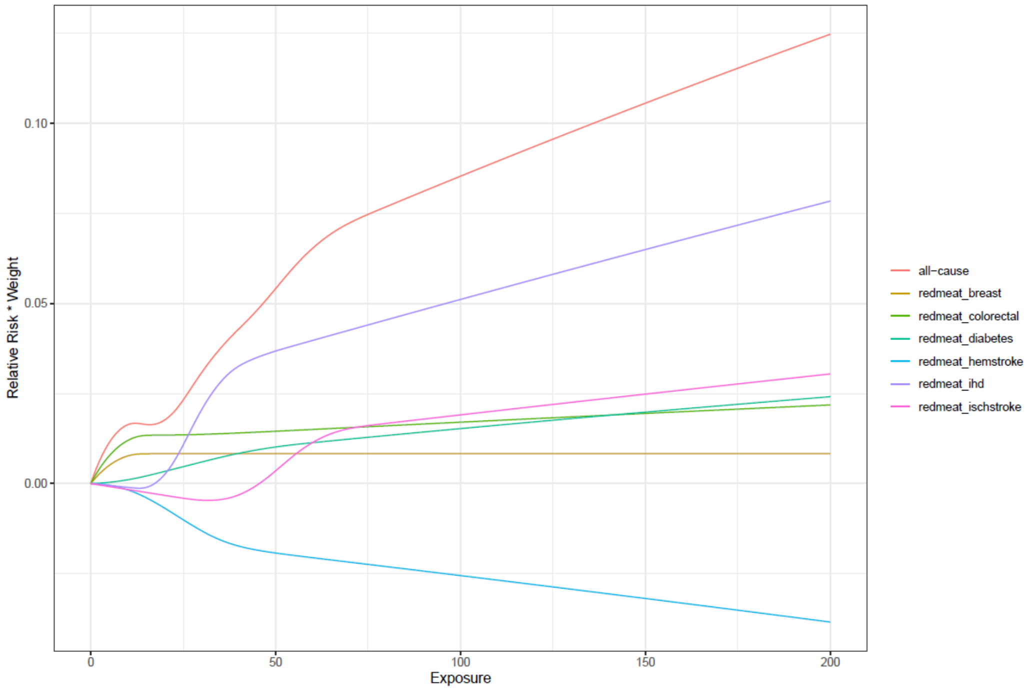

Supplementary Figure 4. The distribution of minimum-risk exposure draws for the across-cause relative risk curve

For each draw of relative risk across a continuum of intake (0-200 g/day), we determined the intake value that minimized the risk, plotting them here. When summarizing this distribution we get that 0 g/day minimizes the mean risk curve with an uncertainty of 0-200 g/day.

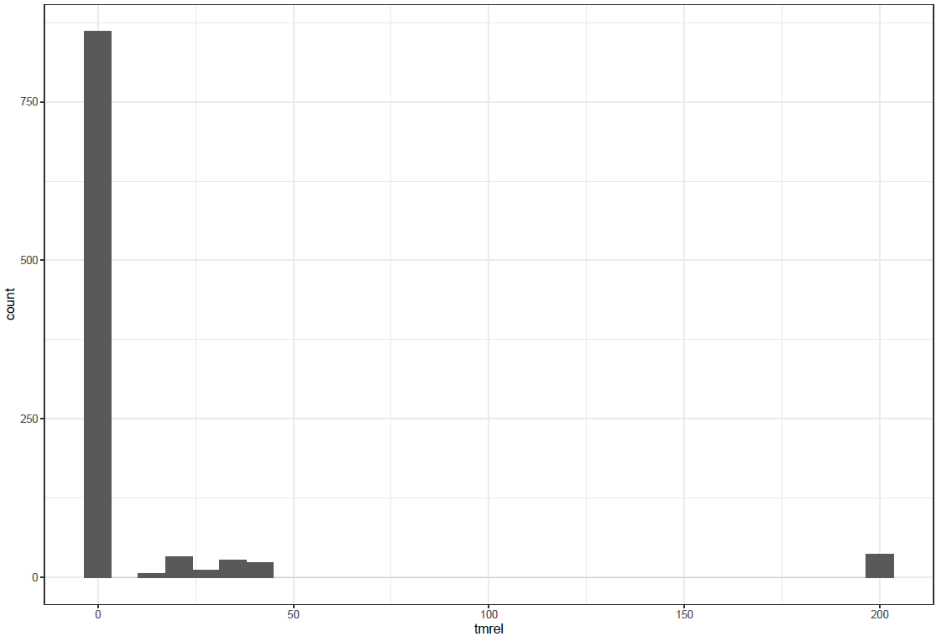

Section 2.3.2: Sensitivity result, DALYs weighting

For a sensitivity analysis, we repeated the across-cause risk curve, but instead of using mortality weights from the GBD, we used Disability Adjusted Life Years (DALY) weights to perform the across-cause aggregation. DALYs are a GBD metric that capture the burden associated with both mortality and morbidity. Thus outcomes that have lower mortality rates but higher prevalence, like diabetes, will have higher weights when using DALYs.

The weights varied marginally compared to the mortality weights: breast cancer 5%, colorectal cancer 5.5%, IHD 42%, hemorrhagic stroke 18%, ischemic stroke 14.5%, and diabetes 15%, though the aggregate curve is nearly identical.

Supplementary Figure 5. Aggregate across-cause relative risk curve for red meat consumption, DALYs weighting

The dark line indicates the across-cause mean relative risk curve. The light shading reflects the conservative uncertainty interpretation (inclusive of between-study heterogeneity), while the dark shading indicates the conventional uncertainty interpretation (exclusive of between-study heterogeneity).

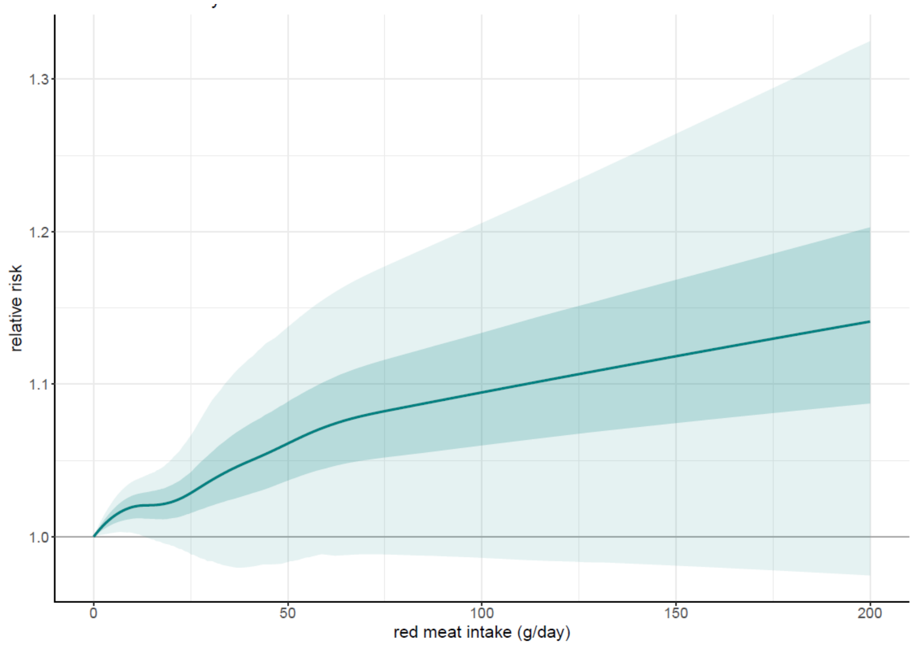

Section 2.3.2: Sensitivity result, only two-star outcomes

For a sensitivity analysis, we repeated the across-cause mortality risk curve analysis with only those outcomes that received 2-stars: colorectal cancer, breast cancer, diabetes, and ischemic heart disease. The resulting across-cause risk curve was minimized at 0 (95% 0-0) g/day.

Supplementary Figure 6. Aggregate across-cause relative risk curve for red meat consumption, only two-star outcomes

The dark line indicates the across-cause mean relative risk curve. The light shading reflects the conservative uncertainty interpretation (inclusive of between-study heterogeneity), while the dark shading indicates the conventional uncertainty interpretation (exclusive of between-study heterogeneity).

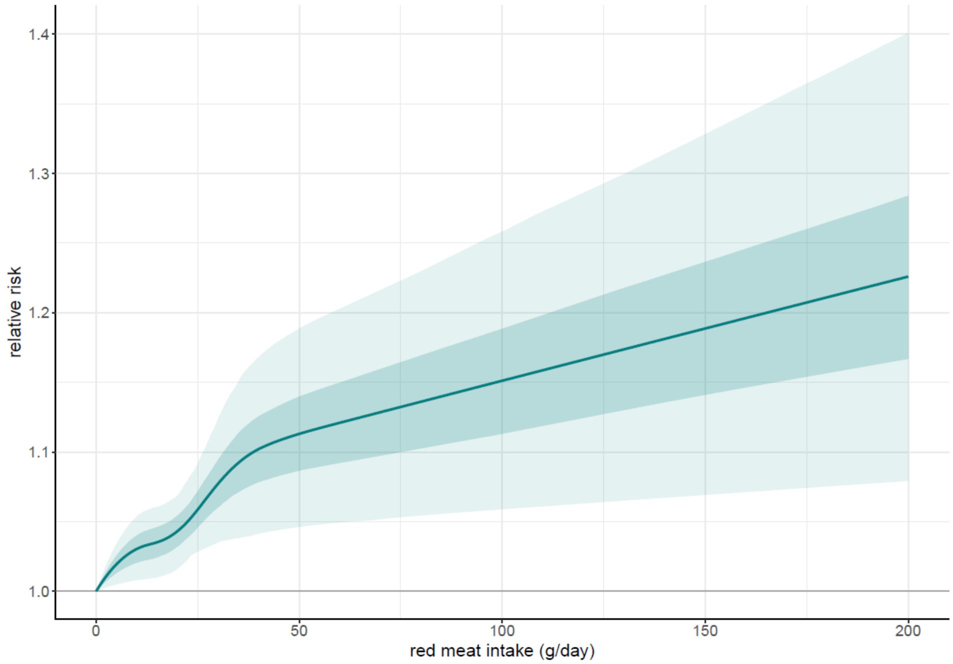

Supplementary Figure 7. The mean of the across-cause relative risk curve, relative to 0, as the sum of the additive components, only 2-star outcomes

Specifically, the across-cause risk curve line (red) is represented with mean relative risk on the y-axis and exposure (consumption in g/day) on the x-axis. It was calculated as the direct sum of each of the four 2-star outcome-specific risk curves (excluding ischemic stroke and hemorrhagic stroke), which are presented with the y-axis as relative risk*weight (breast cancer 5.7%, colorectal cancer 8.8%, IHD 73.7%, type 2 diabetes 12%).

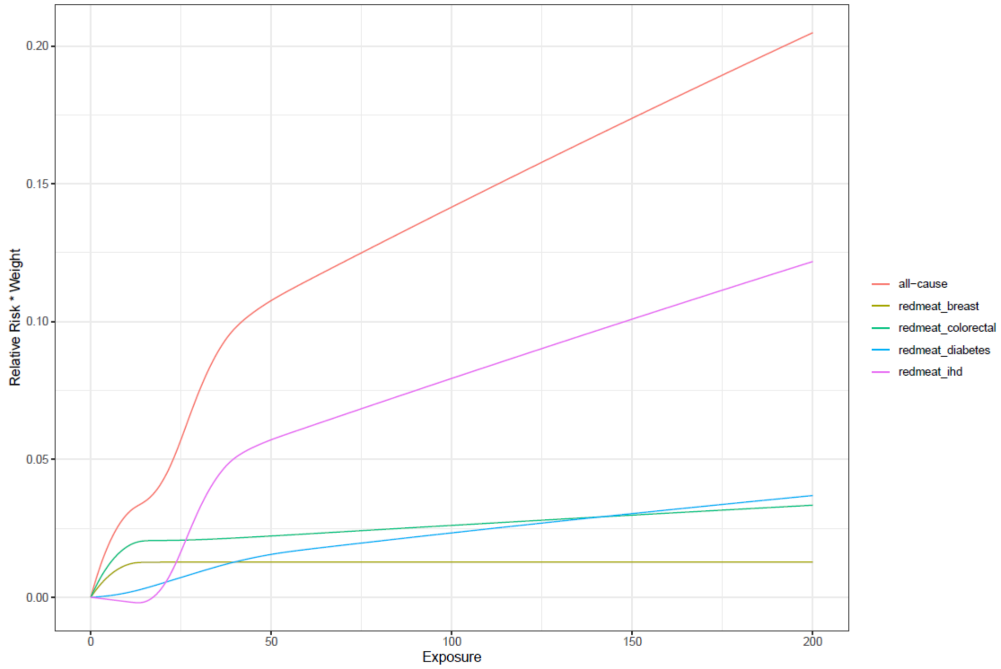

Supplementary Figure 8. The distribution of minimum-risk exposure draws for the across-cause risk curve, only 2-star outcomes

For each draw of relative risk across a continuum of intake (0-200 g/day), we determined the intake value that minimized the risk, plotting them here. When summarizing this distribution we get that 0 g/day minimizes the mean risk curve with an uncertainty of 0-0 g/day.

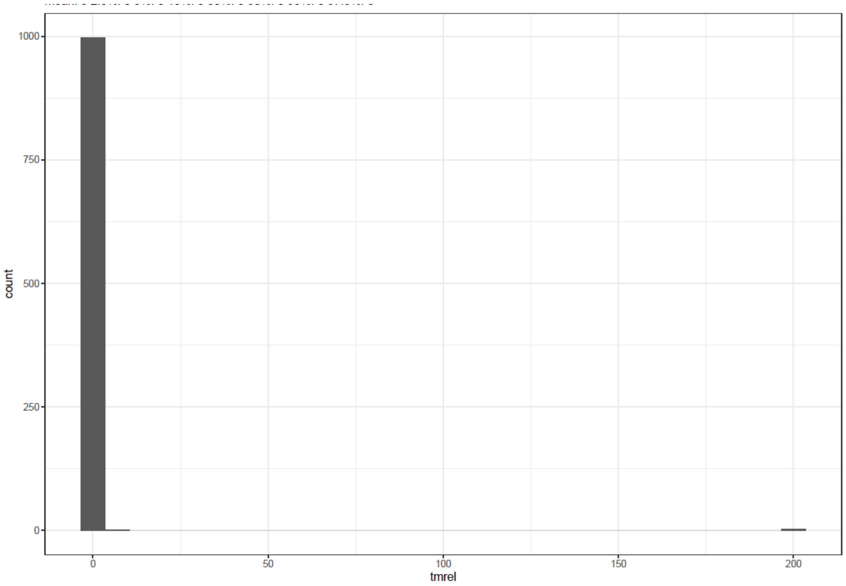

Section 3: Study quality and risk of bias assessment

For each study that met the inclusion criteria, two reviewers assessed several indicators of bias during the extraction process. The full list of bias covariates assessed across all studies can be found in the extraction template (Supplementary Table 6).

To assist with a broad sense of data quality, we calculated a quality score for each study used in this analysis based on four study characteristics that were most applicable and likely to introduce bias (see Supplementary Table 2). The overall score assessment was measured from 0 to 5, where 0 indicated the least bias and 5 indicated the most bias. All studies received a score of at least 1, since red meat consumption was always self-reported from a food frequency questionnaire or a diet history interview. Most studies (n = 55) also provided objective measurements of the reported outcome (component score = 0) (49 of 55) and provided moderate adjustment for confounders (component score = 1) (45 of 55). The mean score across all cohorts and outcomes was 2.8, with 29 studies receiving a score of 3, 17 receiving a score of 2, and 9 receiving a score of 4. The bias covariates that contributed to each of these scores were considered for inclusion in the spline models.

Supplementary Table 2. Study quality for every study used in the models

In this presentation of the data, there is one row per study/outcome pair

**Report**	**Cohort name**	**Outcome**	**Exposure Measurement Score (multiple prospective measurements-0, single baseline prospective measurement-1)**	**Exposure Assessment Score (objective-0 vs self-report-1)**	**Outcome Assessment Score (objective-0 vs self-report-1)**	**Confounders Score (age,sex,smoking, income or education-0; age,sex,smoking-1; age,sex-2)**	**Quality Score (best-0, worst-5)**
Al Rajabi 2022	Alberta's Tomorrow Project	Colon and rectum cancer	1	1	0	0	2

Al-Shaar 2020	Health Professionals Follow-Up Study	Ischemic heart disease	0	1	0	1	2
Bernstein 2010	Nurses' Health Study	Ischemic heart disease	1	1	0	1	3
Bernstein 2012	Health Professionals Follow-Up Study	Hemorrhagic stroke	0	1	0	1	2
Bernstein 2012	Health Professionals Follow-Up Study	Ischemic stroke	0	1	0	1	2
Bernstein 2012	Nurses' Health Study	Hemorrhagic stroke	0	1	0	1	2
Bernstein 2012	Nurses' Health Study	Ischemic stroke	0	1	0	1	2
Diallo 2018	NutriNet-Sante Study	Breast cancer	1	1	0	1	3
Egeberg 2013	Danish, Diet, Cancer and Health	Colon and rectum cancer	1	1	0	1	3
English 2004	Melbourne Collaborative Cohort Study	Colon and rectum cancer	1	1	0	2	4
Ericson 2015	MDC study	Diabetes mellitus type 2	1	1	0	1	3
Etemadi 2017	NIH-AARP Diet and Health Study	Diabetes mellitus type 2	1	1	0	1	3
Fraser* 1999	Adventist Health Study	Ischemic heart disease	1	1	0	1	3
Fretts 2012	Strong Heart Family study	Diabetes mellitus type 2	1	1	0	1	3
Genkinger 2013	Black Women's Health Study	Breast cancer	0	1	0	1	2
Gilsing 2015	Netherlands Cohort Study	Colon and rectum cancer	1	1	0	1	3
Gilsing 2016	Netherlands Cohort Study	Breast cancer	1	1	0	0	2
Haring 2014	Atherosclerosis Risk in Communities Study (ARIC)	Ischemic heart disease	0	1	0	1	2
Inoue-Choi 2016	NIH-AARP Diet and Health Study	Breast cancer	0	1	0	1	2
InterAct Consortium 2013	European Prospective Investigation into Cancer and Nutrition (EPIC)	Diabetes mellitus type 2	1	1	0	1	3
Jarvinen 2001	Finnish Mobile Clinic Health Examination Survey	Colon and rectum cancer	1	1	0	1	3
Jones 2019	Iowa Women's Health Study	Colon and rectum cancer	1	1	0	2	4
Kabat 2007	Canadian National Breast Screening Study	Breast cancer	1	1	0	1	3
Key 2019	European Prospective Investigation into Cancer and Nutrition (EPIC)	Ischemic heart disease	1	1	0	1	3
Knuppel 2020	UK BIOBANK	Breast cancer	1	1	0	0	2
Knuppel 2020	UK BIOBANK	Colon and rectum cancer	1	1	0	0	2
Kurotani 2013	Japan Public Health Center-based Prospective study	Diabetes mellitus type 2	1	1	1	1	4
Lajous 2012	French E3N cohort	Diabetes mellitus type 2	1	1	1	1	4
Larsson 2005	Swedish Mammography Cohort	Colon and rectum cancer	1	1	0	2	4
Larsson 2011	Cohort of Swedish Men	Hemorrhagic stroke	1	1	0	1	3
Larsson 2011	Cohort of Swedish Men	Ischemic stroke	1	1	0	1	3
Larsson 2011	Swedish Mammography Cohort	Hemorrhagic stroke	1	1	0	1	3
Larsson 2011	Swedish Mammography Cohort	Ischemic stroke	1	1	0	1	3

Mannisto 2010	Alpha-Tocopherol, Beta-Carotene Cancer Prevention study	Diabetes mellitus type 2	1	1	0	1	3
Mehta 2020	Sister Study	Colon and rectum cancer	0	1	0	2	3
Mejborn 2020	Danish National Survey on Diet and Physical Activity	Colon and rectum cancer	0	1	0	1	2
Mills 1989	Adventist Health Study	Breast cancer	0	1	0	1	2
Möller 2021	Danish National Survey on Diet and Physical Activity	Ischemic heart disease	1	1	0	0	2
Montonen 2005	Finnish Mobile Clinic Health Examination Survey	Diabetes mellitus type 2	1	1	0	1	3
Nagao 2012	Japan Collaborative Cohort Study	Ischemic heart disease	1	1	0	2	4
Ollberding 2012	The Multiethnic Cohort Study	Colon and rectum cancer	1	1	0	1	3
Pala 2009	European Prospective Investigation into Cancer and Nutrition (EPIC)	Breast cancer	1	1	0	1	3
Pan 2011	Health Professionals Follow-Up Study	Diabetes mellitus type 2	0	1	1	1	3
Pan 2011	Nurses’ Health Study	Diabetes mellitus type 2	0	1	1	1	3
Pan 2011	Nurses’ Health Study II	Diabetes mellitus type 2	0	1	1	1	3
Papier 2021	UK BIOBANK	Diabetes mellitus type 2	1	1	0	0	2
Papier 2021	UK BIOBANK	Hemorrhagic stroke	1	1	0	0	2
Papier 2021	UK BIOBANK	Ischemic heart disease	1	1	0	0	2
Papier 2021	UK BIOBANK	Ischemic stroke	1	1	0	0	2
Parr 2013	Norwegian Women and Cancer (NOWAC) cohort study	Colon and rectum cancer	1	1	0	1	3
Pietinen 1999	Alpha-Tocopherol, Beta-Carotene Cancer Prevention study	Colon and rectum cancer	1	1	0	1	3
Pouchieu 2014	Supplementation en Vitamines et Mineraux Antioxydants study	Breast cancer	0	1	1	1	3
Singh 1998	Adventist Health Study	Colon and rectum cancer	1	1	0	1	3
Steinbrecher 2011	The Multiethnic Cohort Study	Diabetes mellitus type 2	1	1	0	2	4
Takata 2013	Shanghai Men's Health study	Colon and rectum cancer	1	1	0	0	2
Takata 2013	Shanghai Men's Health study	Hemorrhagic stroke	1	1	0	0	2
Takata 2013	Shanghai Men's Health study	Ischemic heart disease	1	1	0	0	2
Takata 2013	Shanghai Men's Health study	Ischemic stroke	1	1	0	0	2
Takata 2013	Shanghai Women's Health Study	Colon and rectum cancer	1	1	0	0	2
Takata 2013	Shanghai Women's Health Study	Hemorrhagic stroke	1	1	0	0	2
Takata 2013	Shanghai Women's Health Study	Ischemic heart disease	1	1	0	0	2
Takata 2013	Shanghai Women's Health Study	Ischemic stroke	1	1	0	0	2
Talaei 2017	Singapore Chinese health study	Diabetes mellitus type 2	1	1	1	1	4
Taylor 2007	UK Women's Cohort Study	Breast cancer	1	1	0	1	3

Tiermersma 2002	Monitoring Project on Cardiovascular Disease Risk Factors	Colon and rectum cancer	1	1	0	2	4
Tong 2020	European Prospective Investigation into Cancer and Nutrition (EPIC)	Hemorrhagic stroke	1	1	0	0	2
Tong 2020	European Prospective Investigation into Cancer and Nutrition (EPIC)	Ischemic stroke	1	1	0	0	2
van Woudenbergh 2012	Rottersdam study	Diabetes mellitus type 2	1	1	0	1	3
Villegas 2006	Shanghai Women's Health Study	Diabetes mellitus type 2	1	1	1	0	3
Virtanen 2017	Kuopio Ischaemic Heart Disease Risk Factor Study	Diabetes mellitus type 2	1	1	0	0	2
Ward 2016	European Prospective Investigation into Cancer and Nutrition (EPIC)	Colon and rectum cancer	1	1	0	1	3
Wei 2004	Health Professionals Follow-Up Study	Colon and rectum cancer	0	1	0	1	2
Wei 2004	Nurses' Health Study	Colon and rectum cancer	0	1	0	1	2
Whiteman 1999	OXCHECK Study	Ischemic heart disease	1	1	0	1	3
Yiannakou 2022	Black Women's Health Study	Colon and rectum cancer	1	1	0	0	2

Section 4. GATHER and PRISMA checklists

Supplementary Table 3. PRISMA 2020 checklist

Section and Topic	Item #	Checklist item	Location where item is reported
**TITLE**			
Title	1	Identify the report as a systematic review.	Per the journal's request, the title does not include "systematic review". It is, however, in the title of the methods section: "Conducting systematic reviews" and mentioned in the first paragraph of the Results.
**ABSTRACT**			
Abstract	2	See the PRISMA 2020 for Abstracts checklist.	See PRISMA 2020 for Abstracts Checklist below (Supplementary Table 4)
**INTRODUCTION**			
Rationale	3	Describe the rationale for the review in the context of existing knowledge.	"Main" (intro) paragraphs 1 & 2
Objectives	4	Provide an explicit statement of the objective(s) or question(s) the review addresses.	"Main" (intro) paragraph 3
**METHODS**			

Eligibility criteria	5	Specify the inclusion and exclusion criteria for the review and how studies were grouped for the syntheses.	Exclusion criteria summarized in Methods section "conducting systematic reviews" paragraphs 2 & 3; full inclusion and exclusion criteria listed in SI section 5.2; reasons for exclusion and number of studies excluded also provided in PRISMA flow diagram (Extended Data Figure 1)
Information sources	6	Specify all databases, registers, websites, organisations, reference lists and other sources searched or consulted to identify studies. Specify the date when each source was last searched or consulted.	Methods section "conducting systematic reviews": paragraph 1; SI section 5.1
Search strategy	7	Present the full search strategies for all databases, registers and websites, including any filters and limits used.	SI section 5.1.1 (reference given at the end of paragraph 1 of "conducting systematic reviews" methods section)
Selection process	8	Specify the methods used to decide whether a study met the inclusion criteria of the review, including how many reviewers screened each record and each report retrieved, whether they worked independently, and if applicable, details of automation tools used in the process.	Methods section "conducting systematic reviews" paragraph 1
Data collection process	9	Specify the methods used to collect data from reports, including how many reviewers collected data from each report, whether they worked independently, any processes for obtaining or confirming data from study investigators, and if applicable, details of automation tools used in the process.	Methods section "conducting systematic reviews" paragraph 4
Data items	10a	List and define all outcomes for which data were sought. Specify whether all results that were compatible with each outcome domain in each study were sought (e.g. for all measures, time points, analyses), and if not, the methods used to decide which results to collect.	Methods sections "selecting health outcomes" and "conducting systematic reviews" paragraph 3; SI sections 1 & 3
	10b	List and define all other variables for which data were sought (e.g. participant and intervention characteristics, funding sources). Describe any assumptions made about any missing or unclear information.	Methods section "conducting systematic reviews" paragraph 4; full list and definitions of all variables are in Supplementary Table 6; study characteristics for each included study are also listed in Supplemementary Table 1
Study risk of bias assessment	11	Specify the methods used to assess risk of bias in the included studies, including details of the tool(s) used, how many reviewers assessed each study and whether they worked independently, and if applicable, details of automation tools used in the process.	Overview of methods for testing for bias in main text methods section "testing for bias across different study designs and characteristics;" information on study quality and risk of bias assessment found in SI section 3
Effect measures	12	Specify for each outcome the effect measure(s) (e.g. risk ratio, mean difference) used in the synthesis or presentation of results.	"Main", paragraphs 3 & 4; methods "overview" and "estimating the burden of proof risk

			function" sections; effect size results from each input study are listed in Supplementary Table 8
Synthesis methods	13a	Describe the processes used to decide which studies were eligible for each synthesis (e.g. tabulating the study intervention characteristics and comparing against the planned groups for each synthesis (item #5)).	Description of processes available in SI section 5
	13b	Describe any methods required to prepare the data for presentation or synthesis, such as handling of missing summary statistics, or data conversions.	Methods section "conducting systematic reviews" paragraph 4
	13c	Describe any methods used to tabulate or visually display results of individual studies and syntheses.	Methods sections "conducting systematic reviews" paragraph 4, "estimating the shape of the risk-outcome relationship"; Section 2 & Supplementary Figure
	13d	Describe any methods used to synthesize results and provide a rationale for the choice(s). If meta-analysis was performed, describe the model(s), method(s) to identify the presence and extent of statistical heterogeneity, and software package(s) used.	Methods sections "Estimating the shape of the risk-outcome relationship," "quantifying between-study heterogeneity...," "determining minimum risk consumption level," and "estimating the burden of proof risk function". Software packages described in "code availability" section of the manuscript
	13e	Describe any methods used to explore possible causes of heterogeneity among study results (e.g. subgroup analysis, meta-regression).	Methods section "quantifying between-study heterogeneity, accounting for heterogeneity, uncertainty, and small numbers of studies"
	13f	Describe any sensitivity analyses conducted to assess robustness of the synthesized results.	SI section 2: sensitivity results (reference to these results found in paragraph 2 of the main text results "overview")
Reporting bias assessment	14	Describe any methods used to assess risk of bias due to missing results in a synthesis (arising from reporting biases).	Methods for detecting publication or reporting bias found in methods section "evaluating potential for publication or reporting bias" and SI section 3
Certainty assessment	15	Describe any methods used to assess certainty (or confidence) in the body of evidence for an outcome.	Methods section "quantifying between-study heterogeneity, accounting for heterogeneity, uncertainty, and small numbers of studies"
**RESULTS**			
Study	16a	Describe the results of the search and selection process, from the number of records	PRISMA flow diagram

selection		identified in the search to the number of studies included in the review, ideally using a flow diagram.	(Extended Data Figure 1)
	16b	Cite studies that might appear to meet the inclusion criteria, but which were excluded, and explain why they were excluded.	N/A
Study characteristics	17	Cite each included study and present its characteristics.	Supplementary Table 1 ("study characteristics"); citations also available for download from the online viz tools (https://vizhub.healthdata.org/burden-of-proof/).
Risk of bias in studies	18	Present assessments of risk of bias for each included study.	Results section "risk of bias assessment;" details in SI section 3 and specifically, Supplementary Table 2 ("study quality for every study used in the models")
Results of individual studies	19	For all outcomes, present, for each study: (a) summary statistics for each group (where appropriate) and (b) an effect estimate and its precision (e.g. confidence/credible interval), ideally using structured tables or plots.	SI section 7 (Supplemental Table 8)
Results of syntheses	20a	For each synthesis, briefly summarise the characteristics and risk of bias among contributing studies.	First paragraph of each results section, excluding those titled "minimum-risk level of red meat intake" and "risk of bias assessment"
	20b	Present results of all statistical syntheses conducted. If meta-analysis was done, present for each the summary estimate and its precision (e.g. confidence/credible interval) and measures of statistical heterogeneity. If comparing groups, describe the direction of the effect.	Second paragraph of each results section + section titled "minimum-risk level of red meat intake;" Figures 1-4; Table 1; Extended Data Figures 2-4; Supplementary Table 7 ("relative risks across exposure range")
	20c	Present results of all investigations of possible causes of heterogeneity among study results.	All uncertainty intervals presented everywhere in the manuscript and supplementary information reflect between-study heterogeneity (unless specified otherwise); BPRFs, ROSs, and star-ratings for each risk-outcome pair also reflect between-study heterogeneity
	20d	Present results of all sensitivity analyses conducted to assess the robustness of the synthesized results.	SI section 2 (reference to these results given in paragraph 2 of the Results "overview" section)
Reporting biases	21	Present assessments of risk of bias due to missing results (arising from reporting biases) for each synthesis assessed.	Results section "risk of bias assessment;" funnel plots (Figures 1C–3C); Extended Data Figures

			2C-4C) SI section 2.2 for results without trimming
Certainty of evidence	22	Present assessments of certainty (or confidence) in the body of evidence for each outcome assessed.	All estimates are presented with 95% uncertainty intervals. UI values are given alongside all mean estimates in the Results and Discussion sections as well as Table 1; all risk curve figures (Figures 1–4; Extended Data Figures 2-4; Supplementary Figures 1a-f, 2a-f, 5, 6) include shading to depict UI curves (both with and without between-study heterogeneity)
**DISCUSSION**			
Discussion	23a	Provide a general interpretation of the results in the context of other evidence.	Discussion paragraphs 4 & 5
	23b	Discuss any limitations of the evidence included in the review.	Discussion paragraph 8
	23c	Discuss any limitations of the review processes used.	Discussion paragraph 8
	23d	Discuss implications of the results for practice, policy, and future research.	Discussion paragraph 2, 3, & 9
**OTHER INFORMATION**			
Registration and protocol	24a	Provide registration information for the review, including register name and registration number, or state that the review was not registered.	This systematic review was not registered (as noted in paragraph 3 of the Methods overview)
	24b	Indicate where the review protocol can be accessed, or state that a protocol was not prepared.	This systematic review was not registered
	24c	Describe and explain any amendments to information provided at registration or in the protocol.	This systematic review was not registered
Support	25	Describe sources of financial or non-financial support for the review, and the role of the funders or sponsors in the review.	"Acknowledgments" section of the manuscript
Competing interests	26	Declare any competing interests of review authors.	"Competing interests" section of the manuscript
Availability of data, code and other materials	27	Report which of the following are publicly available and where they can be found: template data collection forms; data extracted from included studies; data used for all analyses; analytic code; any other materials used in the review.	"Data availability" and "code availability" sections in the manuscript; data collection form template: Supplementary Table 6

Supplementary Table 4. PRISMA 2020 abstract checklist

Section and Topic	Item #	Checklist item	Reported (Yes/No)
**TITLE**			
Title	1	Identify the report as a systematic review.	Per the journal's request, the title does not include

			"systematic review". It is however in the abstract and in the title of the methods section, "Conducting systematic reviews"
**BACKGROUND**			
Objectives	2	Provide an explicit statement of the main objective(s) or question(s) the review addresses.	Yes
**METHODS**			
Eligibility criteria	3	Specify the inclusion and exclusion criteria for the review.	Not in abstract, just main text + supplementary information (given word count limitations by the journal)
Information sources	4	Specify the information sources (e.g. databases, registers) used to identify studies and the date when each was last searched.	Not in abstract, just main text + supplementary information (given word count limitations by the journal)
Risk of bias	5	Specify the methods used to assess risk of bias in the included studies.	Not in abstract, just main text + supplementary information (given word count limitations by the journal)
Synthesis of results	6	Specify the methods used to present and synthesise results.	Yes
**RESULTS**			
Included studies	7	Give the total number of included studies and participants and summarise relevant characteristics of studies.	Not in abstract, just main text + supplementary information (given word count limitations by the journal)
Synthesis of results	8	Present results for main outcomes, preferably indicating the number of included studies and participants for each. If meta-analysis was done, report the summary estimate and confidence/credible interval. If comparing groups, indicate the direction of the effect (i.e. which group is favoured).	Yes, though number of included studies and participants only reported in the main text
**DISCUSSION**			
Limitations of evidence	9	Provide a brief summary of the limitations of the evidence included in the review (e.g. study risk of bias, inconsistency and imprecision).	Not in abstract, just main text + supplementary information (given word count limitations by the journal)
Interpretation	10	Provide a general interpretation of the results and important implications.	Yes
**OTHER**			
Funding	11	Specify the primary source of funding for the review.	Not in abstract, just main text (given abstract standards by the journal)
Registration	12	Provide the register name and registration number.	No

Supplementary Table 5. GATHER checklist

Item #	Checklist item	Reported on page #
Objectives and funding		
1	Define the indicator(s), populations (including age, sex, and geographic entities), and time period(s) for which estimates were made.	Main text methods overview, paragraph 2

2	List the funding sources for the work.	Main text acknowledgement section
Data Inputs		
For all data inputs from multiple sources that are synthesized as part of the study:		
3	Describe how the data were identified and how the data were accessed.	Main text methods section "conducting systematic reviews": paragraphs 1 & 4; SI section 5
4	Specify the inclusion and exclusion criteria. Identify all ad-hoc exclusions.	Exclusion criteria summarized in Methods section "conducting systematic reviews" paragraphs 2 & 3; full inclusion and exclusion criteria listed in SI section 5.2; reasons for exclusion and number of studies excluded also provided in PRISMA flow diagram (Extended Data Figure 1)
5	Provide information on all included data sources and their main characteristics. For each data source used, report reference information or contact name/institution, population represented, data collection method, year(s) of data collection, sex and age range, diagnostic criteria or measurement method, and sample size, as relevant.	Supplementary Table 1 ("study characteristics"); citations also available for download from the online viz tools (https://vizhub.healthdata.org/burden-of-proof/).
6	Identify and describe any categories of input data that have potentially important biases (e.g., based on characteristics listed in item 5).	Results section "risk of bias assessment;" details in SI section 3 and specifically, Supplementary Table 2 ("study quality for every study used in the models")
For data inputs that contribute to the analysis but were not synthesized as part of the study:		
7	Describe and give sources for any other data inputs.	N/A
For all data inputs:		
8	Provide all data inputs in a file format from which data can be efficiently extracted (e.g., a spreadsheet rather than a PDF), including all relevant meta-data listed in item 5. For any data inputs that cannot be shared because of ethical or legal reasons, such as third-party ownership, provide a contact name or the name of the institution that retains the right to the data.	As stated in the Data Availability Statement, data inputs in excel format available for download from the online viz tools (https://vizhub.healthdata.org/burden-of-proof/).
Data analysis		
9	Provide a conceptual overview of the data analysis method. A diagram may be helpful.	Main text methods overview; PRISMA flow diagram (Extended Data Figure 1)
10	Provide a detailed description of all steps of the analysis, including mathematical formulae. This description should cover, as relevant, data cleaning, data pre-processing, data adjustments and weighting of data sources, and mathematical or statistical model(s).	Main text methods; See Zheng et al. for additional detail
11	Describe how candidate models were evaluated and how the final model(s) were selected.	Main text methods "model validation" section
12	Provide the results of an evaluation of model performance, if done, as well as the results of any relevant sensitivity analysis.	Main text methods "model validation" section; SI section 2
13	Describe methods for calculating uncertainty of the estimates. State which sources of uncertainty were, and were not, accounted for in the uncertainty analysis.	Main text methods "quantifying between-study heterogeneity, accounting for heterogeneity, uncertainty, and small numbers of studies" and "estimating the shape of the risk-outcome relationship" (paragraph 2) sections
14	State how analytic or statistical source code used to generate estimates can be accessed.	Code availability statement in the main text
Results and Discussion		

15	Provide published estimates in a file format from which data can be efficiently extracted.	Estimates can be downloaded from the online viz tools (https://vizhub.healthdata.org/burden-of-proof/).
16	Report a quantitative measure of the uncertainty of the estimates (e.g. uncertainty intervals).	UIs given for all findings, including in the text, figures, and tables in the main text and SI sections 2, 6, & 7; online viz tools (see information above)
17	Interpret results in light of existing evidence. If updating a previous set of estimates, describe the reasons for changes in estimates.	Main text discussion paragraphs 4 & 5
18	Discuss limitations of the estimates. Include a discussion of any modelling assumptions or data limitations that affect interpretation of the estimates.	Main text discussion paragraph 8

Section 5: Data source identification and assessment

The data used for this study can be categorized into the following types: prospective cohort, case-cohort, and nested case-control. Underlying data and citations are available for download using the "download" button on each risk curve page at https://vizhub.healthdata.org/burden-of-proof/.

Section 5.1: Literature identification

We conducted literature searches to obtain input data from prospective studies evaluating the relationship between consumption of unprocessed red meat and each of the six outcomes in our analysis. We also searched citation lists of systematic reviews and meta-analyses of prospective observational studies.

Section 5.1.1: Identification of studies via databases

Literature searches were performed on PubMed on May 10, 2022, using the following search string, and extracted reports published through the search date.

PubMed search string: "meat"[tiab] AND "cohort"[tiab]

To ensure we were capturing the most recent literature, searches were also performed on EMBASE and Web of Science from January 1, 2020 to May 10, 2022 using the same search string as above. Of note, there were no additional reports included in our final set that originated from the EMBASE or Web of Science searches that were not captured by our PubMed search.

Section 5.1.2: Identification of studies via other methods

In addition to searching databases, we identified records by searching the citations of reports included in systematic reviews and meta-analyses and by including reviewer-suggested reports. Ten of the systematic reviews and meta-analyses were from existing GBD collection, while eleven of the systematic reviews and meta-analyses were obtained through a systematic search.

Searches for systematic reviews and meta-analyses were performed on PubMed for reports examining the relationship between red meat consumption and each of the six outcomes of interest. The searches for the systematic reviews and meta-analyses were conducted by risk-outcome pair on May 10, 2022, in the PubMed database.

PubMed search strings to identify systematic reviews and meta-analyses for citation searching:

Search string for colorectal cancer:

("Red Meat" [Mesh] OR "red meat" [Title/Abstract])

AND ("Systematic Review" [Publication Type] OR "systematic review" [Title/Abstract] OR "Meta-Analysis" [Publication Type] OR "meta-analysis" [Title/Abstract])

AND ("Colorectal Neoplasms" [Mesh] OR (("colorectal" [Title/Abstract]OR "colon" [Title/Abstract] OR "colonic" [Title/Abstract] OR "rectum" [Title/Abstract] OR "rectal" [Title/Abstract]) AND ("cancer" [Title/Abstract] OR "neoplasm" [Title/Abstract])))

Search string for breast cancer:

("Red Meat" [Mesh] OR "red meat" [Title/Abstract])

AND ("Systematic Review" [Publication Type] OR "systematic review" [Title/Abstract] OR "Meta-Analysis" [Publication Type] OR "meta-analysis" [Title/Abstract])

AND ("Breast Neoplasms" [Mesh] OR ("breast" [Title/Abstract] AND ("cancer" [Title/Abstract] OR "neoplasm" [Title/Abstract])))

Search string for ischemic heart disease:

("Red Meat" [Mesh] OR "red meat" [Title/Abstract])

AND ("Systematic Review" [Publication Type] OR "systematic review" [Title/Abstract] OR "Meta-Analysis" [Publication Type] OR "meta-analysis" [Title/Abstract])

AND ("Myocardial Ischemia" [Mesh] OR "Coronary Artery Disease" [Mesh] OR "Angina, Stable" [Mesh] OR "Acute Coronary Syndrome" [Mesh] OR "ischemic heart disease" [tiab] OR "ischaemic heart disease" [tiab] OR "coronary artery disease" [tiab] OR "coronary heart disease" [tiab] OR "myocardial ischemia" [tiab] OR "myocardial ischaemia" [tiab] OR "myocardial infarction" [tiab] OR "angina" [tiab] OR "acute coronary syndrome")

Search string for type 2 diabetes:

("Red Meat"[Mesh] OR "red meat" [Title/Abstract])

AND ("Systematic Review" [Publication Type] OR "systematic review" [Title/Abstract] OR "Meta-Analysis" [Publication Type] OR "meta-analysis" [Title/Abstract])

AND ("Diabetes Mellitus, Type 2" [Mesh] OR "diabetes mellitus type 2" [Title/Abstract] OR "diabetes type 2" [Title/Abstract] OR "type 2 diabetes mellitus" [Title/Abstract] OR "type 2 diabetes" [Title/Abstract] OR "non-insulin dependent diabetes" [Title/Abstract] OR "adult-onset diabetes" [Title/Abstract])

Search string for ischemic stroke:

("Red Meat"[Mesh] OR "red meat" [Title/Abstract])

AND ("Systematic Review" [Publication Type] OR "systematic review" [Title/Abstract] OR "Meta-Analysis" [Publication Type] OR "meta-analysis" [Title/Abstract])

AND ("Ischemic Stroke" [Mesh] OR "ischemic stroke" [Title/Abstract] OR "ischaemic stroke" [Title/Abstract] OR "cerebral infarction" [Title/Abstract] OR "unspecified stroke" [Title/Abstract] OR "stroke" [Title/Abstract])

Search string for hemorrhagic stroke:

("Red Meat" [Mesh] OR "red meat" [Title/Abstract])

AND ("Systematic Review" [Publication Type] OR "systematic review" [Title/Abstract] OR "Meta-Analysis" [Publication Type] OR "meta-analysis" [Title/Abstract])

AND ("Hemorrhagic Stroke" [Mesh] OR ("subarachnoid" [Title/Abstract] AND ("hemorrhage" [Title/Abstract] OR "haemorrhage" [Title/Abstract])) OR "stroke" [Title/Abstract])

Reviews were screened for methodological quality, as well as compatible exposure, outcomes, included study type, and study design. Reviews were also deduplicated versus the ten reviews from the existing GBD collection.

Systematic reviews and meta-analyses were included for identification through citation-searching if they:

- Report type: were systematic review or meta-analysis (not umbrella review) AND
- Type of studies included by systematic review or meta-analysis: included cohort studies AND
- Exposure: analyzed unprocessed red meat with grams or servings equivalent AND
- Outcome: reported relative risk for outcomes of interest: incidence of type 2 diabetes, ischemic heart disease, breast cancer, colorectal cancer, ischemic stroke, or hemorrhagic stroke AND
- Methodological Quality: followed PRISMA, MOOSE, or similar guidelines AND
- Language: were in English

We gathered citations from the list of included reports from each suitable review. These citations were deduplicated based on citation information among themselves and versus records already captured in our identification.

See Extended Data Figure 1 for details on the systematic review and Supplementary Table 1 for details on study characteristics.

Section 5.2: Assessing data source eligibility

See Extended Data Figure 1 below for details on identifying, screening, and assessing eligibility for records identified through our search.

Reports were included if they:

- Reported a relative risk for unprocessed red meat and one of the six outcomes AND
- Included a measure of uncertainty for the relative risk AND
- Specified the quantity of red meat consumption in the reference and alternate group AND
- Were a cohort study

Reports were excluded if they:

- Were an aggregate study: meta-analysis or pooled cohort
- Had the wrong study type: not a cohort study
- Were a duplicate study: cohort in the report was already included through another report with more person-years of data
- Had unmeasurable exposure: reported red meat consumption without grams or servings equivalent
- Had no measure of interest: reported RR for change in red meat consumption or doesn't report RR
- Did not have exposure of interest: reported on total meat or total red meat instead of unprocessed (fresh) red meat
- Did not have outcome of interest: reported on all-cause-mortality or an outcome outside of the six studied in this paper. This includes outcomes lacking specificity such as total stroke or cardiovascular disease
- Were not in English
- Were a non-general population: study population defined by comorbidity or other traits that could interact with exposure and affect outcome

For reports that met the inclusion criteria, data were extracted for the variables listed in Supplementary Table 6.

Supplementary Table 6. Causal criteria extraction template

Category	Variable	Definition
Source	seq	
	underlying_nid	Underlying NID: Enter the underlying NID of the study (if applicable). Always talk to a data indexer if you don't know if an underlying NID is needed. They may be used for meta-analyses, certain database sources, and in some other specific cases.
	nid	Found in GHDx, created through the epi form, or created by Data Indexer
	field_citation_value	IHME Zotero format or if source has NID, citation info from GHDx
	file_path	optional; full file path of article; Only needed if source doesn't have NID, to facilitate NID creation.
R-O pair	risk	Risk: Select the risk factor, if not listed here, contact the causal criteria team
	risk_mapping	the relationship between study definition of risk and GBD definition of risk for a particular effect size
	outcome	Outcome: Select the outcome.
	outcome_mapping	the relationship between study definition of outcome and GBD definition of outcomefor a particular effect size
Location	location_name	location name (from locations tab). Do a fast double-click in this field to get the drop-down menu, then start typing the location_name. For location_names with special characters, you may need to use the scroll bar.
	location_id	autopopulated from location_name
	rep_geography	Were the study partcipants representative of the geography? 1=yes, 0=no
	rep_selection_criteria	If rep_geography is 0, please specify the selection criteria of the study that is used in the analysis

	rep_prevalent_disease	Is the study aiming to evaluate the risk or mortality of people who have already developed the outcome? 1=yes 0=no (i.e. yes if for SBP-IHD paper, all partcipants have IHD at baseline and the paper is looking at mortality due to SBP, no if for SBP-IHD paper the participants have other prevalent diseases)
Study Population	year_start_study	year the study was started. If not specified, leave blank
	year_end_study	year the study was finished (including most recent follow up). If not specified, leave blank
	age_start	ages from 1 and above must be entered as an integer. Ages <1 can be entered as decimal values, e.g., 3 days = 3/365.
	age_end	ages from 1 and above must be entered as an integer. Ages <1 can be entered as decimal values, e.g., 3 days = 3/365.
	age_mean	Mean age
	age_sd	SD of age
	age_issue	0 = no issue flagged; 1 = issue flagged for modeler; always include explanatory notes the note_SR column
	percent_male	what percent of the population is male (0-1), if pop is all female then it would be 0
	sex_issue	sex_issue
Study Design	design	Study design: Specify the design of the study
	study_name	Study Name: Enter the name of the study (e.g., Nurses' Health Study), if provided. Do not enter the title of the article.
Exposure	exp_assess_level	Level of exposure assessment: The exposure was assessed…
	exp_instrument	Exposure assessment instrument: Specify the name of the exposure assessment instrument. For self-reported exposures, please specify the name of the questionnaire e.g., International Physical Activity Questionnaire (IPAQ). If more than one instrument specify all
	exp_assess_period	What was the frequency of exposure assessment?
	exp_assess_num	if multiple, specify the number of times that exposure was assessed (excluding baseline)
	exp_method_1	Please specify the method of exposure assessment. If there are more than 1, please add in the next columns labeled "exp_method_2".
	exp_method_2	Please specify the method of exposure assessment. If there are more than 2, please add in the next columns labeled "exp_method_3".
	exp_method_3	Please specify the method of exposure assessment.
	exp_recall_period	This field describes the unit of exposure recall used in data collection ONLY for self-report. Select the correct option from the drop-down menu. If the unit is days, weeks, months, or years, please enter the number in exp_recall_period_value (next column). If the unit is 'lifetime', nothing needs to be entered in exp_recall_period_value. For example, if the study said the recall period was 4 weeks, enter 4 in exp_recall_period_value, and 'weeks' in the field exp_recall_period. If 'other' is selected, please describe in exp_recall_period_other
	exp_recall_period_value	If you entered days, weeks, months, or years in the field 'exp_recall_period', please enter the corresponding integer in this field. For example, if the study said the recall period was 4 weeks, enter 4 in exp_recall_period_value, and 'weeks' in the field exp_recall_period.
	exp_recall_period_other	If 'other' was selected in exp_recall_period, please describe the exposure recall period that the study specified (e.g., recall of exposure from 12 to 18 years).
	exp_type	Which form of the exposure was included in relative risk estimation analysis?
Outcome	outcome_def	Outcome definition: Provide a brief description of the outcome as reported in the study.
	outcome_type	Outcome type: please specify if the outcome definition included incidence of or mortality from a disease endpoint
	outcome_assess_1	Method of outcome assessment: Specify the method of assessment of the study outcome. If more than 1 are appropriate, enter additional methods in the next column labeled "outcome_assess_2"
	outcome_assess_2	Method of outcome assessment: Specify the method of assessment of the study outcome. If more than 2 are appropriate, enter additional methods in the next column labeled "outcome_assess_3"
	outcome_assess_3	Method of outcome assessment: Specify the method of assessment of the study outcome.
Follow up	duration_fup_measure	Type of follow up measure (i.e. mean, median, max, min)
	duration_fup_units	Units of follow up duration

	value_of_duration_fup	Enter the length of participant follow-up.
Confounders	confounders_age	if controlled for in the relative risk estimation analysis, mark 1 for yes. Mark 0 for no
	confounders_sex	if controlled for in the relative risk estimation analysis, mark 1 for yes. Mark 0 for no
	confounders_education	if controlled for in the relative risk estimation analysis, mark 1 for yes. Mark 0 for no
	confounders_income	if controlled for in the relative risk estimation analysis, mark 1 for yes. Mark 0 for no
	confounders_smoking	if controlled for in the relative risk estimation analysis, mark 1 for yes. Mark 0 for no
	confounders_alcohol_use	if controlled for in the relative risk estimation analysis, mark 1 for yes. Mark 0 for no
	confounders_physical_activity	if controlled for in the relative risk estimation analysis, mark 1 for yes. Mark 0 for no
	confounders_dietary_components	if controlled for in the relative risk estimation analysis, mark 1 for yes. Mark 0 for no
	confounders_bmi	if controlled for in the relative risk estimation analysis, mark 1 for yes. Mark 0 for no
	confounders_hypertension	if controlled for in the relative risk estimation analysis, mark 1 for yes. Mark 0 for no
	confounders_diabetes	if controlled for in the relative risk estimation analysis, mark 1 for yes. Mark 0 for no
	confounders_hypercholesterolemia	if controlled for in the relative risk estimation analysis, mark 1 for yes. Mark 0 for no
	confounders_other	For other confounders that not listed, list here
Effect Size	page_num_effect_size	Page number (where you found effect_size) from literature, or survey question where you found effect size; Use page number(s) of article, not page # of pdf
	effect_size_measure	Effect size measure: Specify the measure of effect size
	effect_size	Effect size estimate: Provide the effect size estimate
	lower	Provide the lower limit of the confidence interval. Enter on a "per 1" basis. (If the CI is reported as a percent, you must convert to a decimal.) These 3 fields must all be filled in if any of them are filled in: lower, upper, uncertainty_type_value.
	upper	Provide the upper limit of the confidence interval. Enter on a "per 1" basis. (If the CI is reported as a percent, you must convert to a decimal.) These 3 fields must all be filled in if any of them are filled in: lower, upper, uncertainty_type_value.
	CI_uncertainty_type_value	This field is required if 'lower' & 'upper' are entered. This column represents the confidence level which is reported at (Eg. 95, 90, 99). These 3 fields must all be filled in if any of them are filled in: lower, upper, uncertainty_type_value.
	nonCI_uncertainty_value	Numerical value of the nonCI_uncertainty_type entered in that column. For example, if SD=5.3, you'd put 5.3 in this column, and choose SD from the drop down menu in nonCI_uncertainty_type.
	nonCI_uncertainty_type	Enter SE or SD if appropriate. For example, if SD=5.3, you'd put 5.3 in nonCI_uncertainty_value, and choose SD from the drop down menu in this column (nonCI_uncertainty_type).
	uncertainty_issue	Mark with a 1 if no uncertainty is reported, if some sort of uncertainty is reported, mark 0
	subgroup_analysis	1 if RR is from main analysis (all participants), 0 if sub-analysis (only males, or among a specific age group, etc.)
	subgroup_analysis_free_text	if a sub-analysis, describe it (i.e. age, sex, etc.)
	effect_size_multi_location	1 if the reported effect size is from a multi-country study and only one effect size has been reported for all locations, otherwise 0
	effect_size_multi_location_specify	which geography level is the RR for
	pooled_cohort	1 if the reported effect size is from a pooled analysis and only pooled effect size has been reported, otherwise 0
	dose_response	Does the study support a dose-response relationship between the exposure and the outcome? (1= yes, 0=no)
	dose_response_detail	If "1" was specified in the dose_response field, please specify in this field the type of evidence supporting the dose-response relationship. For example, "statistically significant p value for linear trend".
Cohorts	cohort_person_years_exp	Please specify the person years of follow up in the exposed group
	cohort_person_years_unexp	Please specify the person years of follow up in the unexposed group

	cohort_person_years_total	Enter the total person-years of follow-up if person-years of follow up in exposed and unexposed not reported
	cohort_number_events_exp	Please specify the number of events in the exposed group
	cohort_number_events_unexp	Please specify the number of events in the unexposed group
	cohort_number_events_total	Enter the total number of events/cases if number of events in exposed and unexpoxed not reported
	cohort_sample_size_exp	Please specify the number of people in the exposed group if person-years of follow up in exposed not reported
	cohort_sample_size_unexp	Please specify the number of people in the unexposed group if person-years of follow up in unexposed not reported
	cohort_sample_size_total	Please specify the number of people included in the analysis if total person-years of follow up in not reported
	cohort_dropout_rate	Dropout rate: Specify the dropout rate (%) at the end of the study. Enter on a "per 1" basis. For example: 23% is entered as .23.
	cohort_dropout_assess	Specify how dropout rate was defined in the study.
	cohort_exposed_def	exposed group definition: Provide a brief description of the exposed group (i.e., the comparison group) as used in estimation of the relative risk (e.g., never smokers)
	cohort_exp_unit_rr	Exposure unit (for continuous risks): Specify the unit of exposure (e.g., grams/day).
	cohort_exp_level_rr	Exposure level in the exposed group (for continuous risks): Specify the mean/median level of exposure in the exposed group.
	cohort_unexp_def	unexposed group definition: Provide a brief description of the unexposed group (i.e., the comparison group) as used in estimation of the relative risk (e.g., never smokers)
	cohort_unexp_unit_rr	Exposure unit (for continuous risks): Specify the unit of exposure (e.g., grams/day) for the unexposed group
	cohort_unexp_level_rr	Exposure level in the unexposed group (for continuous risks): Specify the mean/median level of exposure in the unexposed group.
	cohort_exp_level_dr	Exposure level in for dose-repose RRs (for continuous risks): If the study reports dose-repose RR, please specify the level of exposure for the reported RR
Case-control	cc_community	Were the controls selected from the community? 1 = yes, 0=no
	cc_cases	Number of cases
	cc_control	Number of controls
	cc_exposed_def	Exposed group definition: Provide a brief description of the exposed group for which the the relative risk is reported (e.g., current smokers)
	cc_exp_unit_rr	Exposure unit (for continuous risks): Specify the unit of exposure (e.g., grams/day).
	cc_exp_level_rr	Exposure level in the exposed group (for continuous risks): Specify the mean/median level of exposure in the exposed group.
	cc_unexposed_def	Unexposed group definition: Provide a brief description of the unexposed group (i.e., the comparison group) as used in estimation of the relative risk (e.g., never smokers)
	cc_unexp_unit_rr	
	cc_unexp_level_rr	Exposure level in the unexposed group (for continuous risks): Specify the mean/median level of exposure in the unexposed group.
	cc_exp_level_dr	Exposure level in for dose-repose RRs (for continuous risks): If the study reports dose-repose RR, please specify the level of exposure for the reported RR
Trials	int_intervention_description	Intervention definition: Provide a brief description of the intervention as reported in the study.
	int_control_description	control definition: Provide a brief description of the control as reported in the study.
	int_intervention_multi_rf	Does this intervention simultaneously target more than one risk? (1=yes, 0=no)
	int_intervention_multi_rf_specify	Specify the risks that are targted by the interevention
	int_intervention_level	Level of intervention: The intervention was implemented …
	int_adhere_assess	Specify how adherence was defined in the study.
	int_adhere_rate_intervention	adherence rate in the intervention group; Enter on a "per 1" basis. For example: 23% is entered as .23.
	int_adhere_rate_control	adherence rate in the control group; Enter on a "per 1" basis. For example: 23% is entered as .23.
	int_dropout_rate_intervention	Dropout rate in the intervention group: Specify the dropout rate (%) at the end of the study. Enter on a "per 1" basis. For example: 23% is entered as .23.

	int_dropout_rate_control	Dropout rate in the control group: Specify the dropout rate (%) at the end of the study. Enter on a "per 1" basis. For example: 23% is entered as .23.
	int_dropout_assess	Specify how dropout rate was defined in the study.
	int_blinding	For interventional studies. Blinding: The trial was … (select 1)
	int_exp_unit	For trials, specify the unit of exposure (e.g., mmol/l)
	int_baseline_exp_int	For trials, specify the exposure level in the intervention group at baseline
	int_baseline_exp_comp	For trials, specify the exposure level in the comparison group at baseline
	int_fup_exp_int	For trials, specify the exposure level in the intervention group at the end of the follow-up time
	int_fup_exp_comp	For trials, specify the exposure level in the comparison group at the end of follow up time
	int_fup_exp_int_difference	For trials, please specify the difference of exposure level between baseline and follow up time for the intervention group
	int_fup_exp_comp_difference	For trials, please specify the difference of exposure level between baseline and follow up time for the comparison grouo
	int_person_years_int	Please specify the number of person years of follow up for the intrevention group
	int_person_years_comp	Please specify the number of person years of follow up in the comparison group
	int_number_events_int	For trials, specify the number of cases in the intervention group at the end of follow up
	int_number_events_comp	For trials, specify the number of cases in the control group at the end of follow up
	int_sample_size_int_group_baseline	For trials, specify the sample size in the intervention group at baseline
	int_sample_size_comparison_group_baseline	For trials, specify the sample size in the comparison group at baseline
	int_sample_size_int_group_follow_up	For trials, specify the sample size in the intervention group at the end of the follow-up time
	int_sample_size_comparison_group_follow_up	For trials, specify the sample size in the comparison group at the end of follow up time
Other	note_modeler	for modelers only, audience is modeler, not for correspondence
	note_sr	notes related to extraction, including assumptions, data adjustment, problems with source, any other notes that may be relevant, etc.
	extractor	uwnet id of person who extracted the data
Custom	custom_exp_meas_num	If the exposure level was assessed multiple times at a given time point (e.g., systolic blood pressure), specify the number of measurements at each time point.
	custom_exp_biomarker	If the exposure level was assessed via a biomarker, specify the full name of the biomarker.
	custom_exp_kilometer	Specify the geographical unit of measurement in kilometer (if applicable, e.g., satellite data).
	custom_exp_level_lower	if don't have a mean/midpoint exposure level can use this column in conjecture with the custom_exp_level_upper to enter in a range
	custom_exp_level_upper	if don't have a mean/midpoint exposure level can use this column in conjecture with the custom_exp_level_lower to enter in a range
	custom_unexp_level_lower	if don't have a mean/midpoint exposure level can use this column in conjecture with the custom_outcome_level_upper to enter in a range
	custom_unexp_level_upper	if don't have a mean/midpoint exposure level can use this column in conjecture with the custom_outcome_level_lower to enter in a range
	custom_prospective_lag	specify lag time between exposure assessment and outcome
	custom_age_demographer	A binary flag to identify if ages are presented in demographer notation or not in the source. This value is currently not used to adjust any age_start or age_end values, but in the future, that is the intention; 0 = article does not use demographer notation (4 = 4.00 not 4.99); 1 = article uses demographer notation (4=4.99 not 4.00)
	custom_bmi_menopause_free_text	free text field for bmi team
	custom_cvd_outcome	used for mapping cvd outcomes, free text field
	custom_dm_type	used for documenting diabeties type
	custom_dm_case_defn	used for documenting diabeties definitions, free text
	custom_pmid	to document Pubmed id
	custom_cvd_rep_high_risk	cvd specific, binary, if the study only includes people at high risk for CVD (1 for example if it is only among diabetics)
	custom_drug_class	class of drug being used in intervention, free text

	custom_outcome_primary	outcome is the primary outcome of RCT (1=yes, 0=no)
	custom_outcome_prespecified	outcome is the prespecified outcome of RCT (1=yes, 0=no)
	custom_multipollutant	Are any other pollutants controlled for in the model? 0=no, 1=yes
	custom_pollutants_controlled	if custom_multipollutant=1, list the pollutants controlled for
	custom_PM2.5_model_type	Describe the model used for exposure
	custom_assign_method	How do researchers assign participants to exp? (ex: by home address, by city, nearest zipcode centroid, etc.)
	custom_PM2.5_def	What metric are they using to measure PM2.5 (ex: mean of annual PM2.5 averages for 35-1 year prior to study)
	custom_lag	Do the authors take into account lag? If so, how?
	custom_PM2.5_min	All of these have to do with the spread of the PM2.5 exposure covered by the study. Minimum
	custom_PM2.5_5th	5th percentile
	custom_PM2.5_25th	25th percentile
	custom_PM2.5_50th	Median/50^{th}percentile
	custom_PM2.5_75th	75th percentile
	custom_PM2.5_95th	95th percentile
	custom_PM2.5_max	maximum
	custom_PM2.5_mean	Mean
	custom_PM2.5_stddev	Standard Deviation
	custom_PM2.5_other_measure	Any other measures of the distribution of PM2.5 amongst participants?
	custom_PM2.5_other_measure_description	If so, what are they? (ex: 10th, 90th, IQR)

Section 6: Risk curve details

Supplementary Table 7. Relative risks across exposure range

The relative risk at every 25 grams of red meat consumption from 0 grams to 200 grams for the risk curves presented in the main text (Figures 1-3; Extended Data Figures 2-4). The relative risk values with a conservative 95% UI, as reported in the main text, are consistent with the BPRF approach to include between-study heterogeneity in the uncertainty analysis. Since conventional meta-analyses do not include between-study heterogeneity and report the 95% UI, we have provided those estimates of the relative risk for comparison.

Outcome	*Intake (g/day)*	*Relative risk (conservative 95%UI)*	*Relative risk (conventional 95%UI)*
Ischemic Heart Disease	0	1 (1,1.01)	1 (1,1)
	25	1.03 (1,1.05)	1.03 (1.02,1.04)
	50	1.09 (0.99,1.18)	1.09 (1.05,1.12)
	75	1.1 (0.99,1.21)	1.1 (1.06,1.14)
	100	1.12 (0.99,1.25)	1.12 (1.07,1.16)
	125	1.14 (0.99,1.29)	1.14 (1.08,1.19)
	150	1.16 (0.99,1.33)	1.16 (1.09,1.21)
	175	1.17 (0.99,1.37)	1.17 (1.1,1.23)
	200	1.19 (0.99,1.41)	1.19 (1.11,1.26)
Ischemic Stroke	0	1.03 (0.98,1.07)	1.03 (1.01,1.04)
	25	1 (1,1.01)	1 (1,1)
	50	1.05 (0.97,1.12)	1.05 (1.02,1.07)
	75	1.13 (0.93,1.34)	1.13 (1.06,1.19)
	100	1.15 (0.93,1.4)	1.15 (1.07,1.22)
	125	1.17 (0.92,1.45)	1.17 (1.07,1.25)
	150	1.19 (0.91,1.51)	1.19 (1.08,1.28)
	175	1.21 (0.9,1.58)	1.21 (1.09,1.31)
	200	1.24 (0.89,1.64)	1.24 (1.1,1.34)
Hemorrhagic Stroke	0	1 (1,1)	1 (1,1)
	25	0.94 (0.8,1.13)	0.94 (0.89,1)
	50	0.9 (0.64,1.26)	0.9 (0.8,1)
	75	0.89 (0.6,1.31)	0.89 (0.78,1)
	100	0.87 (0.56,1.35)	0.87 (0.75,1)
	125	0.86 (0.52,1.41)	0.86 (0.72,1)
	150	0.85 (0.48,1.46)	0.85 (0.7,1)
	175	0.84 (0.45,1.52)	0.84 (0.67,1)
	200	0.83 (0.42,1.58)	0.83 (0.65,1)
Colorectal Cancer	0	1 (1,1)	1 (1,1)
	25	1.28 (1.01,1.58)	1.28 (1.15,1.39)
	50	1.3 (1.01,1.64)	1.3 (1.16,1.43)
	75	1.34 (1.01,1.71)	1.34 (1.17,1.47)
	100	1.37 (1.01,1.78)	1.37 (1.19,1.52)
	125	1.4 (1.02,1.86)	1.4 (1.2,1.57)
	150	1.43 (1.02,1.94)	1.43 (1.22,1.61)
	175	1.47 (1.02,2.02)	1.47 (1.23,1.66)
	200	1.5 (1.02,2.1)	1.5 (1.25,1.71)
Breast Cancer	0	1 (1,1)	1 (1,1)
	25	1.26 (0.98,1.56)	1.26 (1.1,1.4)

	50	1.26 (0.98,1.56)	1.26 (1.1,1.4)
	75	1.26 (0.98,1.56)	1.26 (1.1,1.4)
	100	1.26 (0.98,1.56)	1.26 (1.1,1.4)
	125	1.26 (0.98,1.56)	1.26 (1.1,1.4)
	150	1.26 (0.98,1.56)	1.26 (1.1,1.4)
	175	1.26 (0.98,1.56)	1.26 (1.1,1.4)
	200	1.26 (0.98,1.56)	1.26 (1.1,1.4)
Type 2 Diabetes	0	1 (1,1)	1 (1,1)
	25	1.06 (0.99,1.14)	1.06 (1.04,1.08)
	50	1.14 (0.97,1.32)	1.14 (1.09,1.18)
	75	1.19 (0.96,1.42)	1.19 (1.12,1.24)
	100	1.23 (0.96,1.52)	1.23 (1.14,1.29)
	125	1.27 (0.95,1.62)	1.27 (1.16,1.34)
	150	1.31 (0.94,1.72)	1.31 (1.19,1.39)
	175	1.35 (0.94,1.83)	1.35 (1.21,1.44)
	200	1.39 (0.93,1.94)	1.39 (1.23,1.49)

Section 7: Results from individual studies

The health outcome, reference group exposure, alternative group exposure, log effect size, and log effect size SE identified from each report are provided in Supplementary Table 8.

Supplementary Table 8. Summary results from input studies

Outcome	Report	Reference group exposure	Alternative group exposure	Log effect size	Log effect size standard error
Breast cancer	Diallo 2018	0-0 grams	0-0 grams	0.52	0.16
Breast cancer	Diallo 2018	0-0 grams	0-25 grams	0.46	0.16
Breast cancer	Diallo 2018	0-0 grams	25-42 grams	0.53	0.16
Breast cancer	Diallo 2018	0-0 grams	42-66 grams	0.6	0.16
Breast cancer	Diallo 2018	0-0 grams	0-13 grams	0.52	0.16
Breast cancer	Diallo 2018	0-0 grams	13-37 grams	0.46	0.16
Breast cancer	Diallo 2018	0-0 grams	37-57 grams	0.53	0.16
Breast cancer	Diallo 2018	0-0 grams	57-87 grams	0.6	0.16
Breast cancer	Genkinger 2013	0-14 grams	14-29 grams	0.01	0.1
Breast cancer	Genkinger 2013	0-14 grams	29-43 grams	-0.11	0.12
Breast cancer	Genkinger 2013	0-14 grams	43-57 grams	-0.02	0.15
Breast cancer	Genkinger 2013	0-14 grams	57-71 grams	0.01	0.13
Breast cancer	Genkinger 2013	0-14 grams	14-29 grams	-0.04	0.11
Breast cancer	Genkinger 2013	0-14 grams	29-43 grams	-0.15	0.15
Breast cancer	Genkinger 2013	0-14 grams	43-57 grams	-0.08	0.18
Breast cancer	Genkinger 2013	0-14 grams	57-71 grams	-0.15	0.17
Breast cancer	Gilsing 2016	89-130 grams	0-0 grams	-0.11	0.21

Breast cancer	Gilsing 2016	89-130 grams	0-48 grams	0.15	0.16
Breast cancer	Gilsing 2016	89-130 grams	48-89 grams	0.1	0.15
Breast cancer	Inoue-Choi 2016	0-15 grams	15-25 grams	0	0.04
Breast cancer	Inoue-Choi 2016	0-15 grams	25-37 grams	0.02	0.04
Breast cancer	Inoue-Choi 2016	0-15 grams	37-56 grams	0.07	0.04
Breast cancer	Inoue-Choi 2016	0-15 grams	56-76 grams	0.03	0.04
Breast cancer	Kabat 2007	0-14 grams	14-21 grams	-0.02	0.06
Breast cancer	Kabat 2007	0-14 grams	21-29 grams	0.04	0.06
Breast cancer	Kabat 2007	0-14 grams	29-40 grams	0.03	0.07
Breast cancer	Kabat 2007	0-14 grams	40-52 grams	-0.02	0.07
Breast cancer	Knuppel 2020	0-20 grams	20-39 grams	0	0.05
Breast cancer	Knuppel 2020	0-20 grams	39-50 grams	0.06	0.05
Breast cancer	Knuppel 2020	0-20 grams	50-61 grams	0.09	0.06
Breast cancer	Mills 1989	0-0 grams	0-12 grams	-0.02	0.18
Breast cancer	Mills 1989	0-0 grams	12-24 grams	0.05	0.17
Breast cancer	Pala 2009	0-11 grams	11-29 grams	0.03	0.06
Breast cancer	Pala 2009	0-11 grams	29-45 grams	0.03	0.06
Breast cancer	Pala 2009	0-11 grams	45-70 grams	0.12	0.06
Breast cancer	Pala 2009	0-11 grams	70-94 grams	0.05	0.06
Breast cancer	Pala 2009	0-11 grams	11-29 grams	-0.01	0.08
Breast cancer	Pala 2009	0-11 grams	29-45 grams	-0.04	0.08
Breast cancer	Pala 2009	0-11 grams	45-70 grams	-0.02	0.08
Breast cancer	Pala 2009	0-11 grams	70-94 grams	-0.06	0.08
Breast cancer	Pouchieu 2014	0-0 grams	0-25 grams	-0.2	0.22
Breast cancer	Pouchieu 2014	0-0 grams	25-42 grams	0.05	0.21
Breast cancer	Pouchieu 2014	0-0 grams	42-64 grams	0.17	0.21
Breast cancer	Taylor 2007	0-0 grams	0-32 grams	-0.22	0.19
Breast cancer	Taylor 2007	0-0 grams	32-57 grams	0.17	0.18
Breast cancer	Taylor 2007	0-0 grams	57-82 grams	0.28	0.18
Breast cancer	Taylor 2007	0-0 grams	0-32 grams	0.49	0.18
Breast cancer	Taylor 2007	0-0 grams	32-57 grams	0.49	0.18
Breast cancer	Taylor 2007	0-0 grams	57-82 grams	0.44	0.18
Colon and rectum cancer	Al Rajabi 2022	0-33 grams	33-54 grams	0.11	0.28
Colon and rectum cancer	Al Rajabi 2022	0-33 grams	54-84 grams	0.04	0.29
Colon and rectum cancer	Al Rajabi 2022	0-33 grams	84-114 grams	0.34	0.32
Colon and rectum cancer	Al Rajabi 2022	0-19 grams	19-32 grams	-0.12	0.24
Colon and rectum cancer	Al Rajabi 2022	0-19 grams	32-49 grams	0.11	0.24
Colon and rectum cancer	Al Rajabi 2022	0-19 grams	49-66 grams	-0.15	0.29
Colon and rectum cancer	Egeberg 2013	0-61 grams	61-85 grams	0.03	0.11
Colon and rectum cancer	Egeberg 2013	0-61 grams	85-114 grams	0.2	0.12
Colon and rectum cancer	Egeberg 2013	0-61 grams	114-143 grams	0.17	0.14
Colon and rectum cancer	Egeberg 2013	0-61 grams	61-85 grams	0.04	0.16
Colon and rectum cancer	Egeberg 2013	0-61 grams	85-114 grams	0.04	0.17

Colon and rectum cancer	Egeberg 2013	0-61 grams	114-143 grams	0.1	0.19
Colon and rectum cancer	English 2004	0-0 grams	0-57 grams	0.34	0.14
Colon and rectum cancer	English 2004	0-0 grams	57-86 grams	0.41	0.16
Colon and rectum cancer	English 2004	0-0 grams	86-126 grams	0.34	0.16
Colon and rectum cancer	Gilsing 2015	0-24 grams	24-66 grams	0.17	0.18
Colon and rectum cancer	Gilsing 2015	0-24 grams	66-107 grams	0.23	0.19
Colon and rectum cancer	Gilsing 2015	0-24 grams	107-147 grams	0.18	0.19
Colon and rectum cancer	Gilsing 2015	0-12 grams	12-48 grams	0.17	0.18
Colon and rectum cancer	Gilsing 2015	0-12 grams	48-89 grams	0.23	0.19
Colon and rectum cancer	Gilsing 2015	0-12 grams	89-130 grams	0.18	0.19
Colon and rectum cancer	Jarvinen 2001	0-94 grams	94-141 grams	0.06	0.28
Colon and rectum cancer	Jarvinen 2001	0-94 grams	142-206 grams	0.44	0.29
Colon and rectum cancer	Jarvinen 2001	0-94 grams	206-270 grams	0.41	0.34
Colon and rectum cancer	Jarvinen 2001	0-61 grams	61-92 grams	0.06	0.28
Colon and rectum cancer	Jarvinen 2001	0-61 grams	93-134 grams	0.44	0.29
Colon and rectum cancer	Jarvinen 2001	0-61 grams	134-175 grams	0.41	0.34
Colon and rectum cancer	Jones 2019	0-30 grams	30-49 grams	0.09	0.08
Colon and rectum cancer	Jones 2019	0-30 grams	49-63 grams	0.13	0.08
Colon and rectum cancer	Jones 2019	0-30 grams	63-78 grams	0.17	0.08
Colon and rectum cancer	Jones 2019	0-30 grams	30-49 grams	0.22	0.16
Colon and rectum cancer	Jones 2019	0-30 grams	49-63 grams	0.25	0.16
Colon and rectum cancer	Jones 2019	0-30 grams	63-78 grams	0.14	0.17
Colon and rectum cancer	Knuppel 2020	0-20 grams	20-39 grams	0.1	0.07
Colon and rectum cancer	Knuppel 2020	0-20 grams	39-50 grams	0.14	0.08
Colon and rectum cancer	Knuppel 2020	0-20 grams	50-61 grams	0.21	0.08
Colon and rectum cancer	Larsson 2005	0-24 grams	24-36 grams	0.12	0.09
Colon and rectum cancer	Larsson 2005	0-24 grams	36-49 grams	-0.11	0.13
Colon and rectum cancer	Larsson 2005	0-24 grams	49-61 grams	0.2	0.11
Colon and rectum cancer	Mehta 2020	0-12 grams	12-22 grams	0	0.2
Colon and rectum cancer	Mehta 2020	0-12 grams	22-41 grams	-0.11	0.21
Colon and rectum cancer	Mehta 2020	0-12 grams	41-59 grams	0.04	0.22
Colon and rectum cancer	Mejborn 2020	0-64 grams	65-129 grams	0.01	0.19
Colon and rectum cancer	Ollberding 2012	0-15 grams	15-29 grams	-0.01	0.06
Colon and rectum cancer	Ollberding 2012	0-15 grams	29-44 grams	0	0.06
Colon and rectum cancer	Ollberding 2012	0-15 grams	44-65 grams	-0.03	0.06
Colon and rectum cancer	Ollberding 2012	0-15 grams	65-76 grams	-0.02	0.06
Colon and rectum cancer	Parr 2013	0-5 grams	5-15 grams	0	0.12
Colon and rectum cancer	Parr 2013	0-5 grams	15-25 grams	0.06	0.13
Colon and rectum cancer	Parr 2013	0-5 grams	25-35 grams	-0.2	0.17
Colon and rectum cancer	Parr 2013	0-5 grams	35-45 grams	-0.08	0.21
Colon and rectum cancer	Pietinen 1999	0-44 grams	44-60 grams	-0.51	0.23
Colon and rectum cancer	Pietinen 1999	0-44 grams	60-84 grams	-0.11	0.2
Colon and rectum cancer	Pietinen 1999	0-44 grams	84-108 grams	-0.22	0.22

Colon and rectum cancer	Singh 1998	0-0 grams	0-12 grams	0.46	0.23
Colon and rectum cancer	Singh 1998	0-0 grams	12-24 grams	0.34	0.23
Colon and rectum cancer	Takata 2013	0-22 grams	22-37 grams	-0.01	0.18
Colon and rectum cancer	Takata 2013	0-22 grams	37-52 grams	-0.22	0.2
Colon and rectum cancer	Takata 2013	0-22 grams	52-77 grams	0	0.19
Colon and rectum cancer	Takata 2013	0-22 grams	77-103 grams	-0.19	0.23
Colon and rectum cancer	Takata 2013	0-29 grams	29-46 grams	0.1	0.27
Colon and rectum cancer	Takata 2013	0-29 grams	46-64 grams	0.19	0.28
Colon and rectum cancer	Takata 2013	0-29 grams	64-95 grams	-0.17	0.33
Colon and rectum cancer	Takata 2013	0-29 grams	95-125 grams	0.1	0.34
Colon and rectum cancer	Tiermersma 2002	0-36 grams	38-55 grams	0.99	0.47
Colon and rectum cancer	Tiermersma 2002	0-36 grams	61-78 grams	0.99	0.46
Colon and rectum cancer	Tiermersma 2002	0-36 grams	38-55 grams	-0.22	0.38
Colon and rectum cancer	Tiermersma 2002	0-36 grams	61-78 grams	0.18	0.44
Colon and rectum cancer	Ward 2016	0-20 grams	20-41 grams	-0.09	0.09
Colon and rectum cancer	Ward 2016	0-20 grams	41-72 grams	-0.14	0.1
Colon and rectum cancer	Ward 2016	0-20 grams	72-103 grams	-0.07	0.11
Colon and rectum cancer	Wei 2004	0-0 grams	3-9 grams	0.2	0.31
Colon and rectum cancer	Wei 2004	0-0 grams	9-18 grams	0.15	0.29
Colon and rectum cancer	Wei 2004	0-0 grams	24-49 grams	0.18	0.29
Colon and rectum cancer	Wei 2004	0-0 grams	61-85 grams	0.27	0.3
Colon and rectum cancer	Wei 2004	0-0 grams	3-9 grams	0.31	0.54
Colon and rectum cancer	Wei 2004	0-0 grams	9-18 grams	0.13	0.52
Colon and rectum cancer	Wei 2004	0-0 grams	24-49 grams	0.31	0.52
Colon and rectum cancer	Wei 2004	0-0 grams	61-85 grams	-0.08	0.55
Colon and rectum cancer	Wei 2004	0-0 grams	3-9 grams	0.43	0.21
Colon and rectum cancer	Wei 2004	0-0 grams	9-18 grams	0.3	0.21
Colon and rectum cancer	Wei 2004	0-0 grams	24-49 grams	0.36	0.21
Colon and rectum cancer	Wei 2004	0-0 grams	61-85 grams	0.3	0.27
Colon and rectum cancer	Wei 2004	0-0 grams	3-9 grams	0.24	0.38
Colon and rectum cancer	Wei 2004	0-0 grams	9-18 grams	0.14	0.37
Colon and rectum cancer	Wei 2004	0-0 grams	24-49 grams	0.24	0.37
Colon and rectum cancer	Wei 2004	0-0 grams	61-85 grams	-0.11	0.5
Colon and rectum cancer	Yiannakou 2022	0-12 grams	12-36 grams	0.14	0.13
Colon and rectum cancer	Yiannakou 2022	0-12 grams	36-61 grams	0.25	0.17
Colon and rectum cancer	Yiannakou 2022	0-12 grams	12-36 grams	0.18	0.24
Colon and rectum cancer	Yiannakou 2022	0-12 grams	36-61 grams	0.16	0.29
Diabetes mellitus type 2	Ericson 2015	0-8 grams	8-20 grams	0.1	0.06
Diabetes mellitus type 2	Ericson 2015	0-8 grams	20-30 grams	0.07	0.06
Diabetes mellitus type 2	Ericson 2015	0-8 grams	30-46 grams	0.16	0.06
Diabetes mellitus type 2	Ericson 2015	0-8 grams	46-63 grams	0.22	0.06
Diabetes mellitus type 2	Ericson 2015	0-10 grams	10-16 grams	-0.04	0.06
Diabetes mellitus type 2	Ericson 2015	0-10 grams	16-30 grams	0.04	0.06

Diabetes mellitus type 2	Ericson 2015	0-10 grams	30-46 grams	0.04	0.06
Diabetes mellitus type 2	Ericson 2015	0-10 grams	46-60 grams	0.01	0.06
Diabetes mellitus type 2	Etemadi 2017	0-20 grams	20-35 grams	0.04	0.06
Diabetes mellitus type 2	Etemadi 2017	0-20 grams	35-51 grams	0.16	0.06
Diabetes mellitus type 2	Etemadi 2017	0-20 grams	51-78 grams	0.18	0.06
Diabetes mellitus type 2	Etemadi 2017	0-20 grams	78-108 grams	0.29	0.06
Diabetes mellitus type 2	Fretts 2012	0-23 grams	23-37 grams	0.11	0.19
Diabetes mellitus type 2	Fretts 2012	0-23 grams	37-59 grams	-0.19	0.22
Diabetes mellitus type 2	Fretts 2012	0-23 grams	59-81 grams	-0.13	0.22
Diabetes mellitus type 2	InterAct Consortium 2013	0-21 grams	21-41 grams	-0.01	0.06
Diabetes mellitus type 2	InterAct Consortium 2013	0-21 grams	41-64 grams	0.1	0.05
Diabetes mellitus type 2	InterAct Consortium 2013	0-21 grams	64-97 grams	0.15	0.05
Diabetes mellitus type 2	InterAct Consortium 2013	0-21 grams	97-129 grams	0.18	0.06
Diabetes mellitus type 2	InterAct Consortium 2013	0-14 grams	14-23 grams	-0.01	0.06
Diabetes mellitus type 2	InterAct Consortium 2013	0-14 grams	23-38 grams	0.1	0.05
Diabetes mellitus type 2	InterAct Consortium 2013	0-14 grams	38-66 grams	0.15	0.05
Diabetes mellitus type 2	InterAct Consortium 2013	0-14 grams	66-94 grams	0.18	0.06
Diabetes mellitus type 2	Kurotani 2013	0-23 grams	23-40 grams	0.01	0.12
Diabetes mellitus type 2	Kurotani 2013	0-23 grams	40-66 grams	0.04	0.13
Diabetes mellitus type 2	Kurotani 2013	0-23 grams	66-92 grams	0.41	0.16
Diabetes mellitus type 2	Kurotani 2013	0-20 grams	20-35 grams	0.1	0.13
Diabetes mellitus type 2	Kurotani 2013	0-20 grams	35-57 grams	0.03	0.15
Diabetes mellitus type 2	Kurotani 2013	0-20 grams	57-80 grams	0.04	0.19
Diabetes mellitus type 2	Lajous 2012	0-15 grams	15-42 grams	-0.19	0.08
Diabetes mellitus type 2	Lajous 2012	0-15 grams	42-72 grams	-0.13	0.08
Diabetes mellitus type 2	Lajous 2012	0-15 grams	72-102 grams	-0.05	0.07
Diabetes mellitus type 2	Mannisto 2010	0-40 grams	40-54 grams	0.22	0.1
Diabetes mellitus type 2	Mannisto 2010	0-40 grams	54-68 grams	0.29	0.11
Diabetes mellitus type 2	Mannisto 2010	0-40 grams	68-91 grams	0.17	0.11
Diabetes mellitus type 2	Mannisto 2010	0-40 grams	91-114 grams	0.2	0.12
Diabetes mellitus type 2	Montonen 2005	0-41 grams	41-65 grams	-0.14	0.14
Diabetes mellitus type 2	Montonen 2005	0-41 grams	66-100 grams	-0.19	0.15
Diabetes mellitus type 2	Montonen 2005	0-41 grams	100-134 grams	-0.01	0.17
Diabetes mellitus type 2	Pan 2011	7-20 grams	31-40 grams	-0.05	0.07
Diabetes mellitus type 2	Pan 2011	7-20 grams	48-62 grams	0.13	0.07
Diabetes mellitus type 2	Pan 2011	7-20 grams	73-87 grams	0.05	0.08
Diabetes mellitus type 2	Pan 2011	7-20 grams	110-140 grams	0.25	0.08
Diabetes mellitus type 2	Pan 2011	24-38 grams	48-57 grams	0.1	0.04
Diabetes mellitus type 2	Pan 2011	24-38 grams	64-83 grams	0.1	0.04
Diabetes mellitus type 2	Pan 2011	24-38 grams	82-107 grams	0.15	0.04
Diabetes mellitus type 2	Pan 2011	24-38 grams	113-166 grams	0.21	0.04
Diabetes mellitus type 2	Pan 2011	6-21 grams	31-40 grams	0.01	0.07
Diabetes mellitus type 2	Pan 2011	6-21 grams	48-56 grams	0.12	0.07

Diabetes mellitus type 2	Pan 2011	6-21 grams	65-78 grams	0.11	0.07
Diabetes mellitus type 2	Pan 2011	6-21 grams	95-130 grams	0.24	0.07
Diabetes mellitus type 2	Papier 2021	0-19 grams	19-39 grams	0.05	0.04
Diabetes mellitus type 2	Papier 2021	0-19 grams	39-50 grams	0.14	0.04
Diabetes mellitus type 2	Papier 2021	0-19 grams	50-61 grams	0.15	0.05
Diabetes mellitus type 2	Steinbrecher 2011	0-20 grams	20-35 grams	0.16	0.05
Diabetes mellitus type 2	Steinbrecher 2011	0-20 grams	35-49 grams	0.27	0.05
Diabetes mellitus type 2	Steinbrecher 2011	0-20 grams	49-68 grams	0.35	0.05
Diabetes mellitus type 2	Steinbrecher 2011	0-20 grams	68-180 grams	0.36	0.05
Diabetes mellitus type 2	Steinbrecher 2011	0-12 grams	12-23 grams	0.06	0.06
Diabetes mellitus type 2	Steinbrecher 2011	0-12 grams	23-33 grams	0.16	0.05
Diabetes mellitus type 2	Steinbrecher 2011	0-12 grams	33-48 grams	0.22	0.05
Diabetes mellitus type 2	Steinbrecher 2011	0-12 grams	48-63 grams	0.26	0.05
Diabetes mellitus type 2	Talaei 2017	0-16 grams	16-25 grams	0.04	0.05
Diabetes mellitus type 2	Talaei 2017	0-16 grams	25-36 grams	-0.06	0.05
Diabetes mellitus type 2	Talaei 2017	0-16 grams	36-48 grams	0.1	0.05
Diabetes mellitus type 2	Villegas 2006	0-0 grams	0-24 grams	-0.08	0.07
Diabetes mellitus type 2	Villegas 2006	0-0 grams	24-36 grams	-0.13	0.08
Diabetes mellitus type 2	Villegas 2006	0-0 grams	36-49 grams	-0.29	0.09
Diabetes mellitus type 2	Villegas 2006	0-0 grams	49-68 grams	-0.06	0.09
Diabetes mellitus type 2	Virtanen 2017	0-37 grams	37-66 grams	0	0.14
Diabetes mellitus type 2	Virtanen 2017	0-37 grams	66-106 grams	-0.03	0.14
Diabetes mellitus type 2	Virtanen 2017	0-37 grams	106-144 grams	-0.05	0.14
Diabetes mellitus type 2	van Woudenbergh 2012	0-54 grams	54-75 grams	-0.07	0.15
Diabetes mellitus type 2	van Woudenbergh 2012	0-54 grams	75-98 grams	0	0.15
Diabetes mellitus type 2	van Woudenbergh 2012	0-54 grams	98-121 grams	0.17	0.15
Hemorrhagic stroke	Bernstein 2012	0-41 grams	41-72 grams	-0.33	0.27
Hemorrhagic stroke	Bernstein 2012	0-41 grams	72-114 grams	0.09	0.26
Hemorrhagic stroke	Bernstein 2012	0-41 grams	114-168 grams	-0.51	0.32
Hemorrhagic stroke	Bernstein 2012	0-41 grams	168-221 grams	-0.36	0.34
Hemorrhagic stroke	Bernstein 2012	0-49 grams	49-73 grams	0.11	0.21
Hemorrhagic stroke	Bernstein 2012	0-49 grams	73-105 grams	-0.06	0.23
Hemorrhagic stroke	Bernstein 2012	0-49 grams	105-144 grams	0.33	0.23
Hemorrhagic stroke	Bernstein 2012	0-49 grams	144-183 grams	-0.07	0.28
Hemorrhagic stroke	Larsson 2011	0-34 grams	34-50 grams	-0.13	0.16
Hemorrhagic stroke	Larsson 2011	0-34 grams	50-67 grams	-0.07	0.17
Hemorrhagic stroke	Larsson 2011	0-34 grams	67-83 grams	-0.15	0.18
Hemorrhagic stroke	Larsson 2011	0-34 grams	83-99 grams	0.24	0.18
Hemorrhagic stroke	Larsson 2011	0-16 grams	16-29 grams	-0.11	0.22
Hemorrhagic stroke	Larsson 2011	0-16 grams	29-36 grams	-0.4	0.29
Hemorrhagic stroke	Larsson 2011	0-16 grams	36-49 grams	-0.3	0.26
Hemorrhagic stroke	Larsson 2011	0-16 grams	49-61 grams	-0.19	0.28
Hemorrhagic stroke	Larsson 2011	0-16 grams	16-29 grams	-0.31	0.36

Hemorrhagic stroke	Larsson 2011	0-16 grams	29-36 grams	0.02	0.38
Hemorrhagic stroke	Larsson 2011	0-16 grams	36-49 grams	-0.02	0.36
Hemorrhagic stroke	Larsson 2011	0-16 grams	49-61 grams	-0.11	0.39
Hemorrhagic stroke	Papier 2021	0-19 grams	19-39 grams	-0.04	0.12
Hemorrhagic stroke	Papier 2021	0-19 grams	39-50 grams	0.02	0.12
Hemorrhagic stroke	Papier 2021	0-19 grams	50-61 grams	0.06	0.14
Hemorrhagic stroke	Takata 2013	0-22 grams	22-37 grams	-0.27	0.16
Hemorrhagic stroke	Takata 2013	0-22 grams	37-52 grams	-0.39	0.18
Hemorrhagic stroke	Takata 2013	0-22 grams	52-77 grams	-0.27	0.18
Hemorrhagic stroke	Takata 2013	0-22 grams	77-103 grams	-0.56	0.22
Hemorrhagic stroke	Takata 2013	0-29 grams	29-46 grams	-0.43	0.2
Hemorrhagic stroke	Takata 2013	0-29 grams	46-64 grams	-0.27	0.21
Hemorrhagic stroke	Takata 2013	0-29 grams	64-95 grams	-0.2	0.22
Hemorrhagic stroke	Takata 2013	0-29 grams	95-125 grams	-0.34	0.26
Hemorrhagic stroke	Tong 2020	0-12 grams	12-26 grams	0.01	0.09
Hemorrhagic stroke	Tong 2020	0-12 grams	26-42 grams	-0.04	0.1
Hemorrhagic stroke	Tong 2020	0-12 grams	42-68 grams	0.06	0.1
Hemorrhagic stroke	Tong 2020	0-12 grams	68-93 grams	0	0.11
Ischemic heart disease	Al-Shaar 2020	0-21 grams	21-36 grams	0.1	0.05
Ischemic heart disease	Al-Shaar 2020	0-21 grams	36-51 grams	0.04	0.05
Ischemic heart disease	Al-Shaar 2020	0-21 grams	51-76 grams	0.09	0.06
Ischemic heart disease	Al-Shaar 2020	0-21 grams	76-102 grams	0.17	0.06
Ischemic heart disease	Bernstein 2010	0-31 grams	31-46 grams	-0.09	0.06
Ischemic heart disease	Bernstein 2010	0-31 grams	46-63 grams	-0.01	0.06
Ischemic heart disease	Bernstein 2010	0-31 grams	63-85 grams	-0.07	0.07
Ischemic heart disease	Bernstein 2010	0-31 grams	85-108 grams	0.12	0.07
Ischemic heart disease	Fraser* 1999	0-0 grams	0-36 grams	0.66	0.28
Ischemic heart disease	Fraser* 1999	0-0 grams	36-73 grams	0.84	0.37
Ischemic heart disease	Fraser* 1999	0-0 grams	0-36 grams	-0.21	0.25
Ischemic heart disease	Fraser* 1999	0-0 grams	36-73 grams	-0.27	0.37
Ischemic heart disease	Haring 2014	0-17 grams	17-34 grams	-0.08	0.1
Ischemic heart disease	Haring 2014	0-17 grams	34-47 grams	0.02	0.1
Ischemic heart disease	Haring 2014	0-17 grams	47-72 grams	0.1	0.1
Ischemic heart disease	Haring 2014	0-17 grams	72-98 grams	0.12	0.12
Ischemic heart disease	Key 2019	0-12 grams	12-30 grams	-0.02	0.05
Ischemic heart disease	Key 2019	0-12 grams	30-50 grams	0.05	0.05
Ischemic heart disease	Key 2019	0-12 grams	50-77 grams	0.06	0.05
Ischemic heart disease	Key 2019	0-12 grams	77-104 grams	0.1	0.05
Ischemic heart disease	Möller 2021	0-41 grams	41-97 grams	0.07	0.12
Ischemic heart disease	Möller 2021	0-41 grams	97-153 grams	0.23	0.15
Ischemic heart disease	Nagao 2012	0-16 grams	16-19 grams	0.17	0.17
Ischemic heart disease	Nagao 2012	0-16 grams	19-28 grams	-0.13	0.19
Ischemic heart disease	Nagao 2012	0-16 grams	28-46 grams	0	0.18

Ischemic heart disease	Nagao 2012	0-16 grams	46-63 grams	-0.36	0.2
Ischemic heart disease	Nagao 2012	0-8 grams	8-15 grams	-0.04	0.19
Ischemic heart disease	Nagao 2012	0-8 grams	15-22 grams	-0.34	0.22
Ischemic heart disease	Nagao 2012	0-8 grams	22-35 grams	0.02	0.21
Ischemic heart disease	Nagao 2012	0-8 grams	35-48 grams	0.21	0.21
Ischemic heart disease	Papier 2021	0-19 grams	19-39 grams	0.05	0.04
Ischemic heart disease	Papier 2021	0-19 grams	39-50 grams	0.1	0.04
Ischemic heart disease	Papier 2021	0-19 grams	50-61 grams	0.14	0.04
Ischemic heart disease	Takata 2013	0-22 grams	22-37 grams	0.03	0.16
Ischemic heart disease	Takata 2013	0-22 grams	37-52 grams	0.29	0.17
Ischemic heart disease	Takata 2013	0-22 grams	52-77 grams	-0.19	0.21
Ischemic heart disease	Takata 2013	0-22 grams	77-103 grams	0.25	0.22
Ischemic heart disease	Takata 2013	0-29 grams	29-46 grams	0.04	0.17
Ischemic heart disease	Takata 2013	0-29 grams	46-64 grams	-0.13	0.2
Ischemic heart disease	Takata 2013	0-29 grams	64-95 grams	-0.03	0.2
Ischemic heart disease	Takata 2013	0-29 grams	95-125 grams	0.43	0.21
Ischemic heart disease	Whiteman 1999	0-12 grams	12-36 grams	-0.25	0.25
Ischemic heart disease	Whiteman 1999	0-12 grams	49-85 grams	-0.6	0.3
Ischemic stroke	Bernstein 2012	0-41 grams	41-72 grams	0.23	0.12
Ischemic stroke	Bernstein 2012	0-41 grams	72-114 grams	0.08	0.13
Ischemic stroke	Bernstein 2012	0-41 grams	114-168 grams	0.4	0.14
Ischemic stroke	Bernstein 2012	0-41 grams	168-221 grams	0.21	0.15
Ischemic stroke	Bernstein 2012	0-49 grams	49-73 grams	0.18	0.09
Ischemic stroke	Bernstein 2012	0-49 grams	73-105 grams	0.17	0.09
Ischemic stroke	Bernstein 2012	0-49 grams	105-144 grams	0.15	0.1
Ischemic stroke	Bernstein 2012	0-49 grams	144-183 grams	0.26	0.12
Ischemic stroke	Larsson 2011	0-34 grams	34-50 grams	0.01	0.07
Ischemic stroke	Larsson 2011	0-34 grams	50-67 grams	-0.01	0.07
Ischemic stroke	Larsson 2011	0-34 grams	67-83 grams	-0.09	0.08
Ischemic stroke	Larsson 2011	0-34 grams	83-99 grams	0.02	0.08
Ischemic stroke	Larsson 2011	0-16 grams	16-29 grams	-0.08	0.08
Ischemic stroke	Larsson 2011	0-16 grams	29-36 grams	-0.14	0.1
Ischemic stroke	Larsson 2011	0-16 grams	36-49 grams	0.04	0.09
Ischemic stroke	Larsson 2011	0-16 grams	49-61 grams	0.11	0.09
Ischemic stroke	Papier 2021	0-19 grams	19-39 grams	-0.04	0.08
Ischemic stroke	Papier 2021	0-19 grams	39-50 grams	-0.03	0.08
Ischemic stroke	Papier 2021	0-19 grams	50-61 grams	0.05	0.09
Ischemic stroke	Takata 2013	0-22 grams	22-37 grams	-0.24	0.16
Ischemic stroke	Takata 2013	0-22 grams	37-52 grams	-0.45	0.19
Ischemic stroke	Takata 2013	0-22 grams	52-77 grams	-0.21	0.19
Ischemic stroke	Takata 2013	0-22 grams	77-103 grams	-0.17	0.22
Ischemic stroke	Takata 2013	0-29 grams	29-46 grams	0.02	0.19
Ischemic stroke	Takata 2013	0-29 grams	46-64 grams	-0.2	0.24

Ischemic stroke	Takata 2013	0-29 grams	64-95 grams	-0.09	0.26
Ischemic stroke	Takata 2013	0-29 grams	95-125 grams	0.2	0.29
Ischemic stroke	Tong 2020	0-12 grams	12-26 grams	-0.06	0.05
Ischemic stroke	Tong 2020	0-12 grams	26-42 grams	0	0.05
Ischemic stroke	Tong 2020	0-12 grams	42-68 grams	0.03	0.06
Ischemic stroke	Tong 2020	0-12 grams	68-93 grams	0.09	0.06
