# Supplementary material for: Health effects associated with consumption of unprocessed red meat: a Burden of Proof study
Source: Nat Med. 2022 Oct 10;28(10):2075–82. doi: 10.1038/s41591-022-01968-z (PMC9556326; doi:10.1038/s41591-022-01968-z)
Supplement: Supplementary file 2 — Reporting Summary [file 41591_2022_1968_MOESM2_ESM.pdf]

nature portfolio

Corresponding author(s): Christopher JL Murray

Last updated by author(s): Jul 22, 2022

Reporting Summary

Nature Portfolio wishes to improve the reproducibility of the work that we publish. This form provides structure for consistency and transparency in reporting. For further information on Nature Portfolio policies, see our Editorial Policies and the Editorial Policy Checklist.

Statistics

For all statistical analyses, confirm that the following items are present in the figure legend, table legend, main text, or Methods section.

n/a	Confirmed	
☐	☒	The exact sample size (*n*) for each experimental group/condition, given as a discrete number and unit of measurement
☐	☒	A statement on whether measurements were taken from distinct samples or whether the same sample was measured repeatedly
☐	☒	The statistical test(s) used AND whether they are one- or two-sided<br>*Only common tests should be described solely by name; describe more complex techniques in the Methods section.*
☐	☒	A description of all covariates tested
☐	☒	A description of any assumptions or corrections, such as tests of normality and adjustment for multiple comparisons
☐	☒	A full description of the statistical parameters including central tendency (e.g. means) or other basic estimates (e.g. regression coefficient) AND variation (e.g. standard deviation) or associated estimates of uncertainty (e.g. confidence intervals)
☐	☒	For null hypothesis testing, the test statistic (e.g. *F*, *t*, *r*) with confidence intervals, effect sizes, degrees of freedom and *P* value noted<br>*Give P values as exact values whenever suitable.*
☐	☒	For Bayesian analysis, information on the choice of priors and Markov chain Monte Carlo settings
☒	☐	For hierarchical and complex designs, identification of the appropriate level for tests and full reporting of outcomes
☐	☒	Estimates of effect sizes (e.g. Cohen's *d*, Pearson's *r*), indicating how they were calculated

Our web collection on statistics for biologists contains articles on many of the points above.

Software and code

Policy information about availability of computer code

Data collection	No primary data collection was carried out for this analysis.
Data analysis	All code used for these analyses is publicly available online (https://github.com/ihmeuw-msca/burden-of-proof). This includes code for the meta-regression engine, the model specification interface, both parts of the data processing, and risk-specific custom code, as appropriate. Analyses were carried out using R version 3.6.1, Python version 3.8, and Stata version 17.<br><br>To validate key aspects of the meta-regression model used in this analysis, the following packages were used, as described in Zheng et al: metafor (R package available for download at https://www.jstatsoft.org/article/view/v036i03) and dosmesreta (R package available for download at https://www.jstatsoft.org/article/view/v072c01).

For manuscripts utilizing custom algorithms or software that are central to the research but not yet described in published literature, software must be made available to editors and reviewers. We strongly encourage code deposition in a community repository (e.g. GitHub). See the Nature Portfolio guidelines for submitting code & software for further information.

Data

Policy information about availability of data

All manuscripts must include a data availability statement. This statement should provide the following information, where applicable:

- Accession codes, unique identifiers, or web links for publicly available datasets
- A description of any restrictions on data availability
- For clinical datasets or third party data, please ensure that the statement adheres to our policy

The findings from this study were produced using data available in the published literature. Study sources and citations for each risk-outcome pair can be downloaded using the “download” button on each risk curve page at https://vizhub.healthdata.org/burden-of-proof/. Citations for all input studies are found in the main reference list as reference numbers 24-78. Study characteristics for all input data used in the analyses are also provided in Supplementary Table 1. See Supplementary Table 6 for a template of the data collection form.

Human research participants

Policy information about studies involving human research participants and Sex and Gender in Research.

Reporting on sex and gender	No primary data collection was carried out for this analysis, so the study does not involve human research participants. As stated in the methods overview, our estimates are not specific to or disaggregated by specific populations, including by sex. Because of this, we included all available data regardless of how or if the input study collected and reported data by sex or gender. We did not perform sex- or gender-based analyses due to limitations in and scarcity of the underlying data.
Population characteristics	No primary data collection was carried out for this analysis, so the study does not involve human research participants. The analysis evaluated the effect of unprocessed red meat consumption on selected chronic disease endpoints in adults 25 years through the oldest age group (95+ years).
Recruitment	No primary data collection was carried out for this analysis, so we did not recruit participants.
Ethics oversight	This study was approved by the University of Washington IRB Committee (study #9060).

Note that full information on the approval of the study protocol must also be provided in the manuscript.

Field-specific reporting

Please select the one below that is the best fit for your research. If you are not sure, read the appropriate sections before making your selection.

☒ Life sciences ☐ Behavioural & social sciences ☐ Ecological, evolutionary & environmental sciences

For a reference copy of the document with all sections, see nature.com/documents/nr-reporting-summary-flat.pdf

Life sciences study design

All studies must disclose on these points even when the disclosure is negative.

Sample size	No sample size calculation was performed for this meta-analysis; all available datasets meeting inclusion criteria or included. As reported in the main text results sections for each risk-outcome pair, the number of data points for each of the risk-outcome pairs is as follows: red meat and colorectal cancer: 2,413,032 individuals (14,672 incident cases, 1,530 deaths); red meat and breast cancer: 999,428 individuals (25,732 incident cases); red meat and ischemic heart disease: 1,219,288 individuals {33,490 outcomes); red meat and type 2 diabetes: 1,619,574 individuals (58,364 new cases and 3,717 deaths); red meat and ischemic stroke: 1,185,969 individuals (11,996 incident cases, 504 deaths); and red meat and hemorrhagic stroke: 1,185,969 individuals (3,646 incident cases, 530 deaths).
Data exclusions	As described in Supplementary Information Section 5, reports were excluded based on the following exclusion criteria: were an aggregate study: meta-analysis or pooled cohort; had the wrong study type: not a cohort study; were a duplicate study: cohort reported in paper was also reported elsewhere; had unmeasurable exposure: reported red meat consumption without grams or servings equivalent; had no measure of interest: reported RR for change in red meat consumption or doesn't report RR; did not have exposure of interest: reported on total meat or total red meat instead of unprocessed (fresh) red meat; did not have outcome of interest: reported on all-cause-mortality or an outcome outside of the six studied in this paper. This includes outcomes lacking specificity such as total stroke or cardiovascular disease; were not in English.
Replication	This is a meta-analysis of existing studies with many years of cohort and other data. When re-applying the method to the same data, we get the same results.
Randomization	This analysis is a meta-analysis of existing studies and thus, there were no experimental groups.
Blinding	N/A. Blinding was not relevant to this study, as we did not collect primary data.

Reporting for specific materials, systems and methods

We require information from authors about some types of materials, experimental systems and methods used in many studies. Here, indicate whether each material, system or method listed is relevant to your study. If you are not sure if a list item applies to your research, read the appropriate section before selecting a response.

Materials & experimental systems

n/a	Involved in the study
☒	☐ Antibodies
☒	☐ Eukaryotic cell lines
☒	☐ Palaeontology and archaeology
☒	☐ Animals and other organisms
☒	☐ Clinical data
☒	☐ Dual use research of concern

Methods

n/a	Involved in the study
☒	☐ ChIP-seq
☒	☐ Flow cytometry
☒	☐ MRI-based neuroimaging
